# MLKL and other necroptosis-related genes promote the tumor immune cell infiltration, guiding for the administration of immunotherapy in bladder urothelial carcinoma

**DOI:** 10.1007/s10495-023-01830-8

**Published:** 2023-03-31

**Authors:** Boqiang Zhong, Youzhi Wang, Yihao Liao, Jiaming Liang, Keke Wang, Diansheng Zhou, Yang Zhao, Ning Jiang

**Affiliations:** 1 Tianjin Institute of Urology, The Second Hospital of Tianjin Medical University, Tianjin, 300211 China; 2 Department of Urology, Tangdu Hospital, The Air Force Military Medical University, Xi’an, Shaanxi China; 3 Department of Radiology, The Second Hospital of Tianjin Medical University, Tianjin, 300211 China

**Keywords:** Necroptosis, BUC, Immunotherapy, Chemotherapy, Tumor immune microenvironment, Pan-cancer, Prognosis, Diagnosis, ICIs

## Abstract

The involvement of necroptosis in the immunosuppressive tumor microenvironment has been established and has been shown to contribute to the growth of pancreatic ductal adenocarcinoma, indicating its role in promoting tumor development. However, the relationship between necroptosis and bladder urothelial carcinoma (BUC) has yet to be fully understood. To shed light on this issue, our study aimed to uncover the impact of necroptosis on immune cell infiltration and immunotherapy response in BUC patients. We conducted an analysis of 67 necroptosis genes to assess their expression and genomic changes across pan-cancer and identified 12 necroptosis genes that are prognostically relevant and associated with immune subtypes and tumor stemness in BUC. Using a public database of 1841 BUC samples, we then performed Unsupervised Cluster Analysis and discovered two distinct necroptotic phenotypes in BUC. These phenotypes showed significant differences in molecular subtypes, immune infiltration patterns, and gene mutation profiles. We confirmed this discovery in BUC through qPCR and WB experiments. To evaluate the impact of necroptosis on prognosis, chemotherapy sensitivity, and immunotherapy response (such as anti-PD-L1), we developed a principal component analysis model called NecroScore. Finally, we validated the effects of RIPK3 and MLKL through a nude mouse transplantation model for BUC. Our study has uncovered that necroptosis plays a role in shaping the tumor immune microenvironment in BUC. The high necroptosis phenotype (Cluster B) was characterized by a higher abundance of tumor immunosuppressive cells and more key biological processes driving tumor progression, while the low necroptosis group (Cluster A) had higher FGFR3 mutations. We found that the infiltration levels of immune cells, including CD8+ T cells, were significantly different between FGFR3 mutated and wild-type (WT) samples. Our results confirmed the reliability of NecroScore as a comprehensive assessment tool for evaluating the immunotherapeutic effect and prognosis of BUC patients, with high NecroScore values favoring basal-like differentiation and lower FGFR3 alterations. We also observed that high expression of MLKL had a significant inhibitory effect on tumor growth and increased neutrophil infiltration in vivo. In our study, we uncovered the regulation pattern of necroptosis in the tumor immune microenvironment of BUC. Additionally, we developed a scoring tool called NecroScore that can be utilized to predict the most suitable chemotherapy and immunotherapy strategy for bladder urothelial carcinoma patients. This tool can effectively guide the chemotherapy and immunotherapy regimens for patients with advanced BUC.

**Supplementary Information:**

The online version contains supplementary material available at 10.1007/s10495-023-01830-8.

## Introduction

Bladder cancer (BCa) is a major health concern, ranking as the eleventh most common cancer globally, with an estimated 573,000 new cases and 212,000 deaths annually [1]. The majority of newly diagnosed BCas are non-muscle invasive (NMIBC) and are typically treated with transurethral resection and intravesical chemotherapy or Bacillus Calmette-Guerin (BCG). Muscle-invasive BCa (MIBC) accounts for the remaining cases and can also occur in 10 to 20 percent of NMIBC patients that progress over time [2]. Platinum-based chemotherapy is widely accepted as a first-line treatment strategy for advanced urothelial carcinoma, with the combination of gemcitabine and cisplatin being the most common treatment regimen for BUC [3–5]. For patients with advanced/metastatic urothelial carcinoma that is either platinum-refractory or platinum-ineligible, the FDA has approved a range of treatment options, including PD-1/PD-L1-based immune checkpoint inhibitors (ICIs), FGFR3 inhibitors, and antibody drug conjugates. Pembrolizumab and atezolizumab, two checkpoint inhibitors, have been approved by the FDA and European Medicines Agency (EMA) for first-line treatment in cisplatin-unsuitable patients with a positive PD-L1 status [6].

Necroptosis is a form of immunogenic cell death in which death receptors, such as FAS and tumor necrosis factor receptor 1 (TNFR1), or pattern recognition receptors, such as toll-like receptor 3 (TLR3), trigger the death process in response to adverse signals from the microenvironment [7]. This type of necrosis is heavily dependent on the presence of RIPK3 and MLKL, with MLKL activation being a defining characteristic [8]. RIPK3 activates MLKL by phosphorylating it, causing it to oligomerize and translocate to the cell membrane, where it forms pores, leading to calcium influx and the release of danger-associated molecular patterns (DAMPs). These DAMPs can activate both innate and adaptive immune responses and trigger phagocytosis of dying cells [9]. Necroptosis has been extensively studied in pancreatic ductal adenocarcinoma (PDAC) and has been shown to enhance the tumor microenvironment in a manner dependent on CXCR2- and SAP130-macrophage-induced calcium (Mincle)-dependent paracrine [10]. In PDAC, upregulation of RIPK1 in tumor-associated macrophages contributes to immune tolerance and resistance to immunotherapy [11]. The mechanism by which necroptosis occurs in BUC is not well understood, though previous studies have shown that shikonin, a PKM2 inhibitor, can induce necroptosis in BUC and overcome cisplatin resistance [12].

In our study, we investigated the impact of necroptosis on tumor immune infiltration and immunotherapy efficacy. We started by examining the expression and genetic changes of 67 necroptosis genes across 33 different cancer types and then identified two necroptosis modules in 1841 BUC cases, based on 12 necroptosis genes related to prognosis. We observed strong differentiation between the two modules in terms of differences in tumor immune cell infiltration and gene mutation patterns. Lastly, we developed a scoring system, named NecroScore, to assess the level of necroptosis, which can be used to predict the sensitivity to chemotherapy and the efficacy of immunotherapy. A recent study has demonstrated that providing first-line maintenance Avelumab after initial platinum-containing chemotherapy (in the absence of progressive disease) can significantly enhance the survival rate of patients diagnosed with advanced BUC [13]. Our scoring tool exhibits high accuracy in evaluating the sensitivity of chemotherapy and immunotherapy. Therefore, this tool is of great significance for patients with advanced bladder urothelial carcinoma.

## Materials and methods

### Data sources and process

The research design and methodology are briefly illustrated in Supplementary Fig. 1. Further information on data sources can be found in the Supplementary Methods section [14].

### Construction of necroptosis regulator phenotypes

We analyzed the expression levels of 12 necroptosis-related molecules and summarized the data through univariate Cox analysis and unsupervised cluster analysis [15, 16]. The procedure used to carry out this analysis is detailed in the Supplementary Methods section.

### PCA scoring calculation

For gene expression score analysis, we conducted principal component analysis to extract the first principal component as the gene feature score. Further details on the procedure can be found in the Supplementary Methods section [17].

### Statistical analysis

All statistical analysis was conducted using R software (https://www.r-project.org/). A complete rundown of the procedures can be found in the Supplementary Methods section.

Additional information on bioinformatics methods and experimental methods is also available in the Supplementary Methods.

## Results

### The genetic characteristics and transcriptional variations of 67 necroptosis molecules

This study presents the research ideas and processes through Fig. S1. The study analyzed 67 molecules associated with necroptosis from previous studies [18]. A pan-cancer analysis of necroptosis-related genes was carried out, and the mRNA expression levels of necroptosis molecules in 18 cancer types were evaluated. The analysis was based on the comparison of expression levels between tumor and normal samples obtained from the TCGA database. The results showed that CDKN2A and PLK1 were significantly overexpressed in tumor samples compared to normal samples across the 18 cancer types. Conversely, KLF9 was significantly overexpressed in normal samples compared to tumor samples. The expression levels of MLKL and CASP8 in tumor samples were higher than those in normal tissues in KIRC (Renal Clear Cell Carcinoma), KIRP (Papillary Renal Cell Carcinoma), and BLCA (Bladder Urothelial Carcinoma) (Fig. S2A, S3B, Table S1). Moreover, the study revealed that high expression of PLK1 had a poor prognosis in 11 tumor types, including KIRC, KIRP, etc., while high expression of KLF9 was associated with a better prognosis in KIRC (Fig. S3E). The study also conducted a correlation analysis of the expression of the 67 necroptosis-related molecules among 33 cancer types in the TCGA database. The results showed that MLKL had a strong positive correlation with CASP8, ZBP1, FASLG, RIPK1, and RIPK3 in all 33 cancer types, and RIPK3 was also strongly correlated with CASP8, MLKL, and ZBP1 (Fig. S2C). Additionally, the average expression of TNF-related genes, including TNFRSF1A, TNFRSF1B, TNFSF10, and TNFRSF21, was higher than that of most other necroptosis genes in pan-cancer (Fig. S3A).

Figure S2B illustrates the genetic variation of 67 necroptosis-related molecules in 3907 samples from 32 cancer types in the TCGA pan-cancer database. The 10 most frequently mutated necroptosis molecules are shown through a pan-cancer single nucleotide variant (SNV) analysis using a waterfall diagram. Out of the 3907 cases, 2679 samples (68.57%) had a necroptosis mutation. The mutation with the highest frequency was observed in BRAF (19%), followed by ATRX (15%), IDH1 (13%), CDKN2A (10%), and EGFR (9%), with missense mutation being the most common type. The highest mutation frequency of BRAF, ATRX, IDH1, CDKN2A, and EGFR was seen in melanoma (SKCM), thyroid carcinoma (THCA), brain lower-grade glioma (LGG), head and neck squamous cell carcinoma (HNSC), and glioblastoma multiforme (GBM), respectively (Figs. S2B, S4C). Our analysis of DNA methylation in tumor and normal tissues revealed that KLF9 and GATA3 were significantly higher in tumor tissues compared to normal tissues [Lung squamous cell carcinoma (LUSC), Lung adenocarcinoma (LUAD), Colon adenocarcinoma (COAD), etc.]. Conversely, MLKL and CASP8 were significantly lower in tumor tissues compared to normal tissues (KIRC BLCA LUSC LUAD, etc.) (Fig. S2D). Hypermethylation of KLF9 was associated with lower survival rates in several cancer types such as LGG, thymoma (THYM), and uterine carcinosarcoma (UCS), but hypermethylation of KLF9 in KIRC was associated with a better prognosis in KIRC (Fig. S3F). The results of the spearman correlation analysis between gene methylation and gene expression showed that gene methylation was negatively correlated with gene expression in most of the necroptosis-related genes in 33 cancer types. Interestingly, methylation levels of ALK and BCL2 were positively correlated with mRNA expression levels of ALK and BCL2 in most cancer types, as well as the positive correlation between methylation level of APP and mRNA expression level of APP in liver hepatocellular carcinoma (LIHC) and the positive correlation between methylation level of CD40 and mRNA expression level of CD40 in esophageal carcinoma (ESCA) (Fig. S2E). Our findings confirmed that copy number variation (CNV) is an important factor affecting the expression of necroptosis molecules. The mutation rates of copy number variations (CNV) and the expression levels of mRNA were found to be positively correlated in most necroptosis-related genes in 33 cancer types, especially in the cases of FADD and USP22. Conversely, in the cases of RIPK3 and FASLG, between the CNV mutation rates and the expression levels, showed a negative correlation in THYM and pancreatic adenocarcinoma (PAAD) (Fig. S2F). Heterozygous CNV mutations in necroptosis-associated genes were more prevalent in most tumor types (Fig. S3C, S3D).

In this study, we analyzed the differences in gene expression among the subtypes of necroptosis and investigated the characteristics of the associated signaling pathways. Our findings showed that many important necroptosis molecules in the pan-cancer, including PLK1, MLKL, FASLG, and ZBP1, demonstrated a high level of activation in the apoptosis signaling pathway. Meanwhile, PLK1, DNMT1, TARDBP, and HAT1 showed high activation in the cell cycle. On the other hand, TNFRSF21, TNFRSF1A, STAT3, RIPK1, KLF9, EGFR, and AXL showed high levels of inhibition in both the cell cycle and DNA damage response (Figs. S2G, S4A). Moreover, both MLKL and RIPK3 play a role in inhibiting the cell cycle signaling pathway in BUC and also in inhibiting the DNA damage repair signaling pathway, but only MLKL can promote the apoptosis signaling pathway. In prostate adenocarcinoma (PRAD), both the hormone ER and EMT signaling pathways are activated by MLKL and RIPK3 (Fig. S4B). In our study, we placed a significant emphasis on necroptosis in BUC. Of the 412 samples in BUC, TSC1 and CDKN2A had the highest mutation frequencies of 8% and 6%, respectively, in the TCGA database (Fig. S2H). Our results showed significant co-mutations between CDKN2A and STAT3, CDKN2A and TNFRSF21, CDKN2A and RNF31, ZBP1 and BRAF, FASLG and PLK1, and FASLG and PANX1 in BUC (P < 0.05, Fig. S2I). These findings suggest that the occurrence of BUC is strongly related to the imbalanced regulation of necroptosis-related genes.

### Characteristics of the relationship between 12 prognosis related necroptosis genes and immunity, tumor stemness

**Fig. 1:**
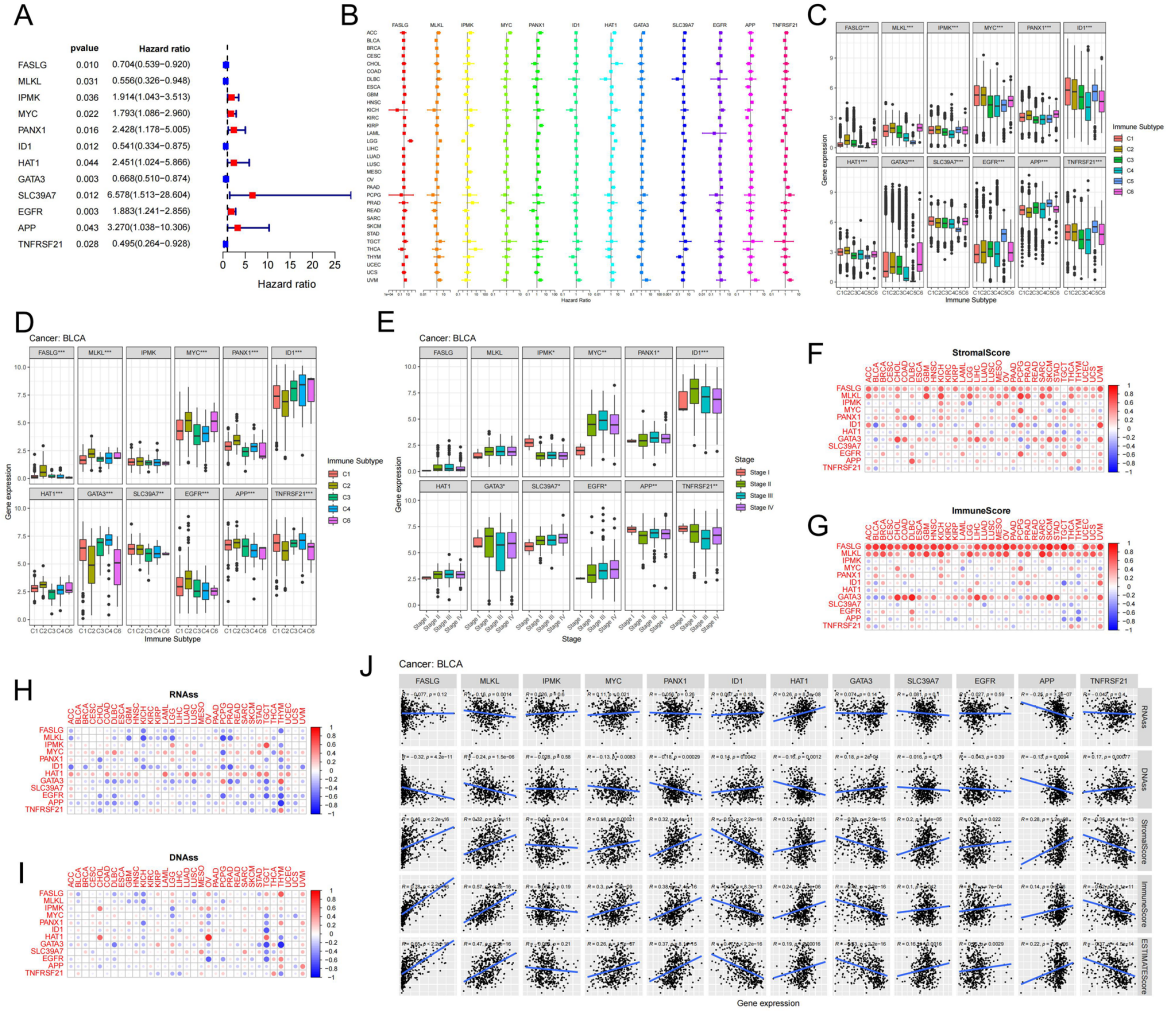
Characteristics of the relationship between 12 prognosis related necroptosis genes and immunity, tumor stemness.** A** Forest plots show the results of Cox regression analysis of the mean survival rates of 12 prognosis-related necroptosis genes in the TCGA-BLCA cohort. **B** Multi-forest plot showing the results of univariate Cox regression analysis of 12 necroptosis genes in pan-cancer. **C** Pearson correlation analysis of 12 necroptosis gene expression and immune subtypes in pan-cancer. **D** Pearson correlation analysis of 12 necroptosis gene expression and immune subtype in TCGA-BLCA cohort. **E** Pearson correlation analysis of 12 necroptosis gene expression and clinical stage in the TCGA-BLCA cohort. **F-G** Using the "estimate" package to calculate the ImmuneScore and StromalScore of tumor tissue in pan-cancer, and then analyze the relationship between the expression of 12 necroptosis genes and them. **H-I** Correlation between the expression of necroptosis genes and tumor stemness in pan-cancer. RNAss represents the tumor stemness index estimated by the expression of classical genes, and DNAss represents the tumor stemness estimated by the methylation modification of classical genes. **J** The plot depicts the association between 12 necroptosis genes and immune-related scores, tumor stemness in BUC

Next, we performed a univariate Cox regression analysis on 67 necroptosis-related genes in BUC and identified 12 prognosis-related genes (P < 0.05, Figs. 1A, S5). These 12 genes play different roles in 33 cancer types (Fig. 1B). Our analysis of the correlation between these 12 necroptosis-related genes and different immune subtypes in Pan-cancer showed significant differences in gene expression among subtypes. The FASLG, MLKL, and HAT1 genes showed the highest expression in the IFN-γ Dominant (C2) and TGF-beta Dominant (C6) subtype, but the lowest expression in the Immunologically Quiet (C5) subtype. Conversely, the EGFR, APP, and TNFRSF21 genes had the highest expression in the C5 subtype (Fig. 1C, Table S12.1). In BUC, the FASLG, MLKL, HAT1, MYC, PANX1 and EGFR gene expression levels were significantly higher in the C2 subtype compared to other immune subtypes including Wound Healing (C1), Inflammatory (C3) and Lymphocyte Depleted (C4) subtypes (Fig. 1D, Table S12.2). The MYC and SLC39A7 gene expressions were lower in stage I compared to stage IV, whereas the IMPK gene expressions were higher in stage I compared to stage IV (Fig. 1E, Table S12.3). We performed an EstimateScore analysis of the 12 prognosis necroptosis genes in Pan-cancer and found that FASLG and MLKL were strongly positively correlated with the StromalScore and ImmuneScore in 33 cancer types, while ID1, GATA3, and TNFRSF21 showed a negative correlation with the StromalScore and ImmuneScore in BUC (Fig. 1F, G, J). The correlation between gene expression and tumor StemnessScore was evaluated to determine the correlation between a necroptosis gene and the degree of tumor differentiation. The StemnessScore was calculated using RNA stemness indices assessed by RNA gene expression and DNA stemness indices assessed by DNA methylation. The StemnessScores in both RNAss and DNAss were negatively correlated with FASLG and MLKL expressions in cancers such as Lymphoid Neoplasm Diffuse Large B-cell Lymphoma (DLBC), GBM, KICH, and LUSC (Fig. 1H, I). Interestingly, we found that the expression of MLKL and APP in BUC was positively correlated with immune cell infiltration, but negatively correlated with tumor stemness (Fig. 1J). Our findings indicate that the 12 necroptosis genes, particularly MLKL, play a crucial role in tumor immune cell infiltration and tumor cell stemness.

### Two different patterns of necroptosis were identified by unsupervised cluster analysis in a cohort of 1841 BUC samples

**Fig. 2:**
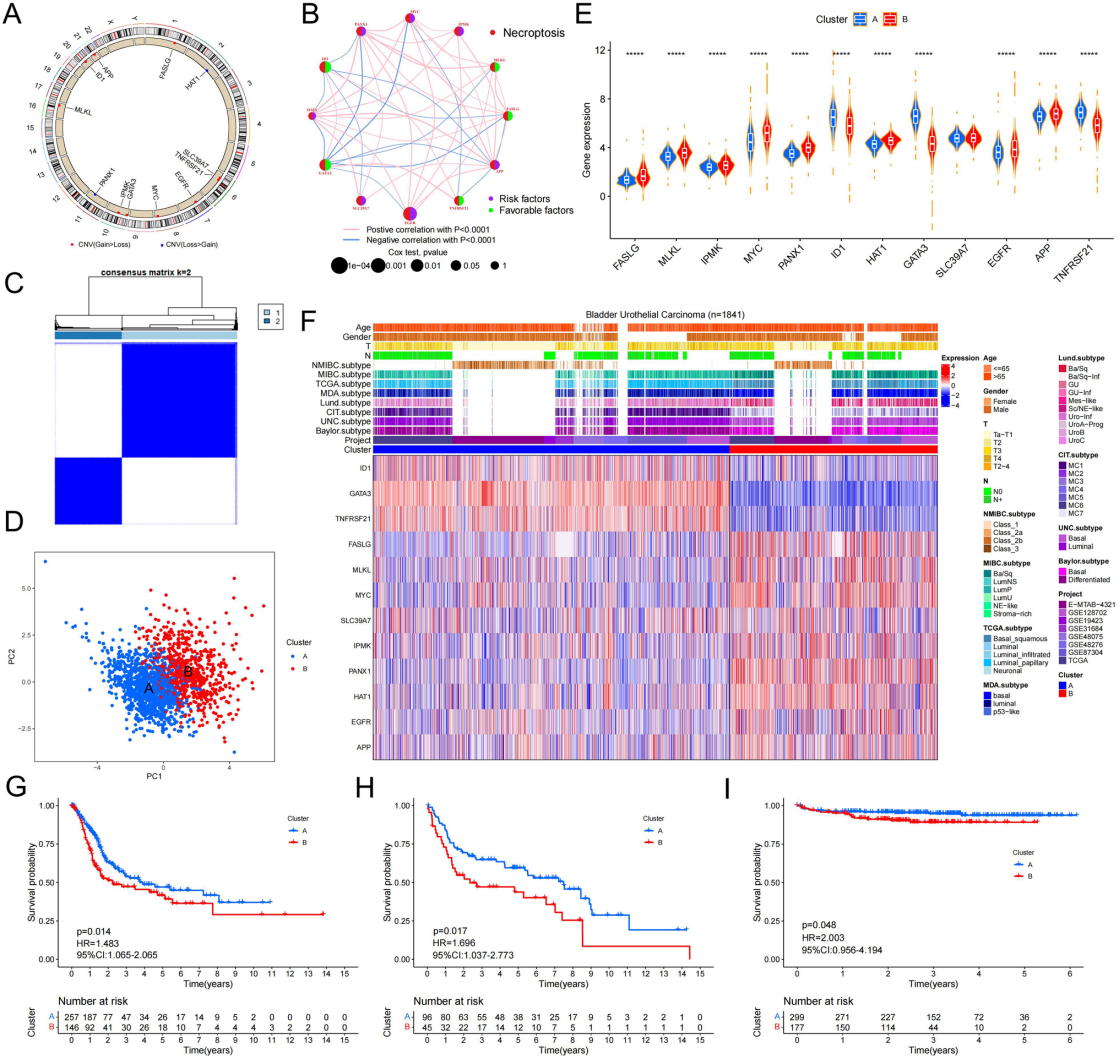
Unsupervised learning to identify two classification of necroptosis. **A** Chromosome circle map accurately shows chromosomal locations and gene copy number changes of 12 necroptosis genes. **B** The network map accurately shows the correlation between the 12 necroptosis genes. The association of 12 genes with prognosis was determined by univariate COX regression analysis. Circle size correlates with P value; green represents prognostic favorable factors; purple represents prognostic risk factors. The red line indicates the positive correlation between the gene expression levels of the two, and vice versa. **C** Unsupervised cluster learning divides 1841 BLCA cohorts into two clusters. **D** Principal component analysis results for two clusters. **E** Expression comparison of 12 necroptosis genes between the two clusters. **F** Composite heatmap shows correlations between two types of necroptosis and molecular subtypes of BUC, and differential expression of 12 necroptosis-related molecules. **G–I** Kaplan–Meier curves show significant differences in survival between the two necroptotic phenotypes in the TCGA, GEO and E-MTAB-4321 databases. G and H show overall survival rates for patients with TCGA and GEO databases respectively, while I shows disease progression survival rates for E-MTAB-4321 datasets (Color figure online)

The chromosome circle diagram and network diagram were constructed to visually display the chromosomal position and the interdependence between the expression of 12 prognosis-related necroptosis genes. In the TCGA database, HAT1 and PANX1 showed greater loss of copy number variation (CNV) compared to gain of CNV, unlike the other 10 genes (Fig. 2A). In the TCGA database, the expression levels of HAT1 and SLC39A7 in tumor tissues were higher than those in adjacent normal tissues, while the expression level of MYC in tumor tissues was lower than that in adjacent normal tissues (Fig. S6A). MLKL, FASLG, ID1, GATA3, and TNFRSF21 were key contributors to the favorable prognosis of BUC patients, compared to the other seven necroptosis-related genes (Figs. 2B, S5). However, unlike the positive correlation between the expression of most of the 12 necroptosis genes, GATA3 displayed a negative correlation with FASLG, APP, MLKL, MYC and PANX1, and similarly, ID1 showed a negative correlation with FASLG and APP (Fig. 2B).

To uncover the regulatory pattern of 12 necroptosis genes, we collected 1841 samples from BUC. We corrected for batch effects by using the 'combat' formula in the SVA package of R language. By performing unsupervised cluster analysis with the ‘ConsensusClusterPlus’ package of R language, we identified two necroptosis-related modules, named Cluster A (1163 cases) and Cluster B (678 cases) (Figs. 2C, S6B–S6E). Principal component analysis showed that the two clusters were differentiated by the expression of the 12 necroptosis genes (Fig. 2D). A differential expression analysis of the 12 genes between the two clusters revealed significant differences in all genes except SLC39A7, with the exception of ID1 and GATA3, which were expressed higher in Cluster B than in Cluster A (Fig. 2E). We used the R packages ‘consensusMIBC’, ;BLCAsubtyping’, and ‘classifyNMIBC’ to determine the molecular subtype of the 1841 BUC samples based on the gene expression matrix. Furthermore, we visualized the gene expression level, clinical features, and molecular typing of the 12 necroptosis genes in 1841 BUC samples using heatmap package (Table S2, Fig. 2F). The proportion of class_1/3 was significantly higher in Cluster A than that in Cluster B in NMIBC.subtype, whereas compared to Cluster A, the proportion of class_2b was significantly higher in Cluster B in NMIBC.subtype (Fig. S6F1–7, Table S13.1). Furthermore, the luminal subtype mainly occurred in Cluster A, while the basal subtype was more prevalent in Cluster B when compared across the 6 typing methods: Baylor.subtype, UNC.subtype, MDA.subtype, MIBC.subtype, and TCGA.subtype (Fig. S6F1–5, Table S13.1). Similarly, in lund.subtype, Cluster A was predominantly composed of UroA-Prog, UroC and GU, while Cluster B was mainly made up of Ba/Sq, Ba/Sq-inf and Mes-like (Fig. S6F6, Table S13.1). In CIT.subtype, MC1 type was predominant in Cluster A, while MC7 type was more abundant in Cluster B (Fig. S6F7, Table S13.1). We also compared the clinical characteristics between the two clusters and found that the proportion of patients with Ta-T1 stage was significantly higher in Cluster A compared to Cluster B, while the proportion of patients with stage T3 was higher in Cluster B compared to Cluster A (Figs. 2F, S6F9, Table S13.1). Finally, by analyzing the overall survival and progression-free survival curves in the TCGA dataset and the GEO dataset and the EMATB dataset, we found that the survival status and time of Cluster A and Cluster B were significantly different (Fig. 2G–I). These results demonstrate that our necroptosis model is successful and highlights the significant correlation between molecular subtype and disease prognosis between the two clusters.

### Differences in signaling pathways, single nucleotide variants and copy number variants in two necroptosis modules

**Fig. 3:**
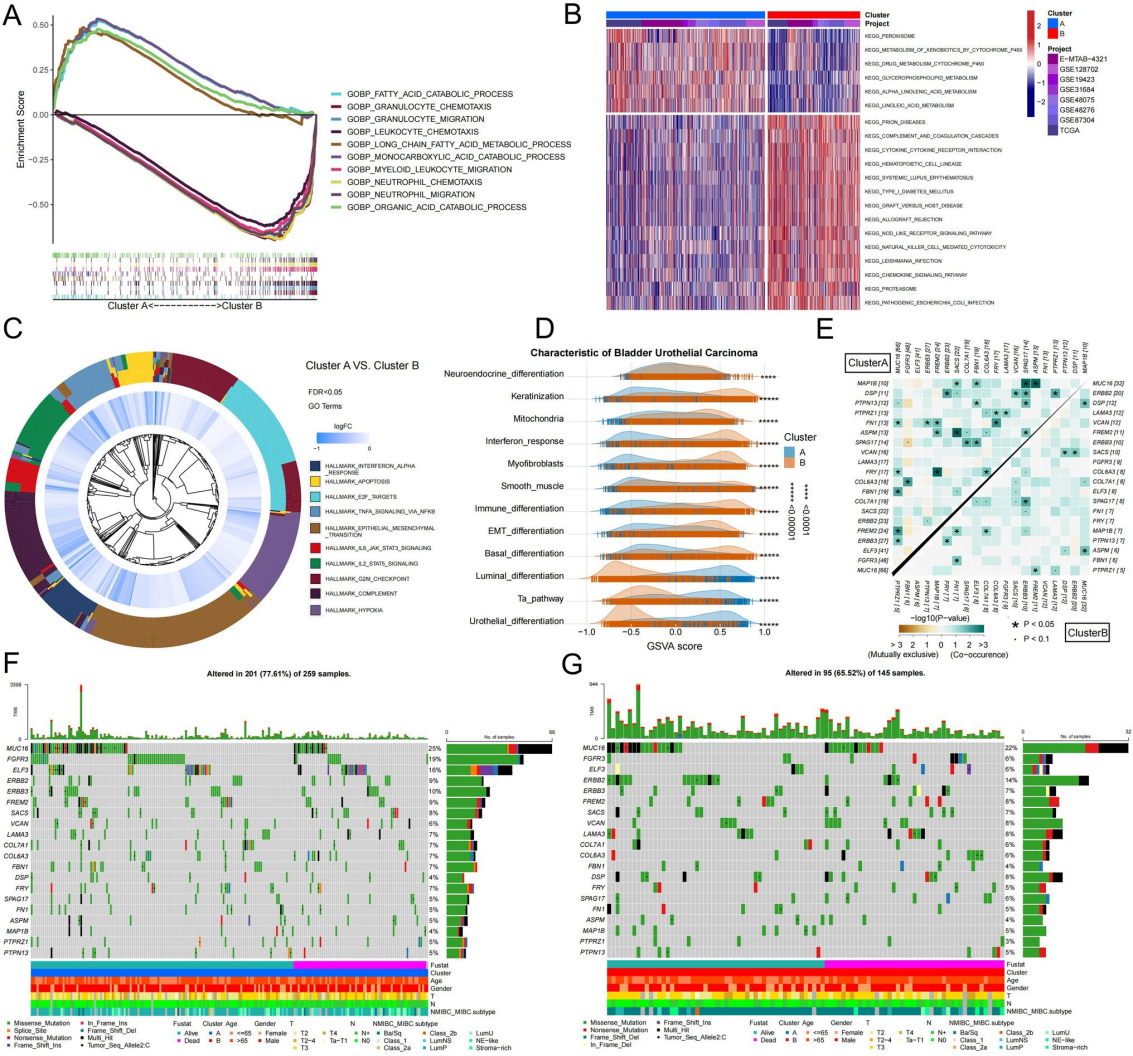
Differences in signaling pathways and genetic mutations of necroptosis modules. We performed differential genetic analysis of two necroptosis modules followed by signaling pathway enrichment. **A** Differences between the two groups were analyzed by multi-gsea enrichment of GO gene set. A positive enrichment score indicates that the signaling pathway is enriched in Cluster A, while a negative enrichment score indicates that the signaling pathway is enriched in Cluster B. The larger the absolute value of the enrichment score, the more genes are enriched in the signaling pathway, and vice versa. **B** The expression of all samples was analyzed by GSVA enrichment analysis of KEGG gene set, and the top 20 enriched signaling pathways are displayed. **C** The circle plot shows the enrichment of the two clusters on the hallmark gene set. LogFC represents the difference between the gene expression level of Cluster A and Cluster B. If it is negative, it means that the gene expression level in Cluster B is higher than that in Cluster A. **D** All samples were subjected to GSVA enrichment analysis of important signaling pathways in BUC. Ridge plot shows the distribution of enrichment scores of important signaling pathway in two clusters of BUC samples. We explored the gene mutational landscape of the two clusters by using the “maftools” package. **E** The correlation heatmap shows the co-mutation of the two groups of differentially expressed genes, and highlights the top 20 genes of gene mutation frequency. Green indicates co-occurrence, and yellow indicates co-exclusion. **F** and **G** Waterfall chart shows the gene mutation frequency and mutation type of the two clusters. The upper part of the figure shows the TMB, and the lower part of the figure shows the patient's clinical information and molecular subtypes. F displays the gene mutation information of Cluster A, while G displays that of Cluster B (Color figure online)

To identify the molecular features, we first conducted a differential analysis of gene expression between the two groups (Table S3). Results from gene set enrichment analysis showed that metabolism-related gene sets such as 'FATTY_ACID_CATABOLIC_PROCESS' and 'MONOCARBOXYLIC_ACID_CATABOLIC_PROCESS' were primarily enriched in Cluster A, while immune-related gene sets such as 'GRANULOCYTE_CHEMOTAXIS,' 'MYELOID_LEUKOCYTE_MIGRATION,' and 'NEUTROPHIL_MIGRATION' were primarily enriched in Cluster B (Fig. 3A). This result was consistent with the findings from KEGG gene set variation analysis (GSVA), which also showed that metabolism-related gene sets were primarily enriched in Cluster A, while Cluster B was not only enriched in immune cell-related signaling pathways, but also in cytokine-related signaling pathways (Fig. 3B). Additionally, hallmark gene set enrichment analysis (GSEA) revealed that Cluster B was enriched in apoptosis-related and hypoxia-related gene sets, as well as cytokine-related signaling pathways such as 'IL2_STAT5_SIGNALING,' 'IL6_JAK_STAT3_SIGNALING' (Fig. 3C, Table S4). We further collected gene sets of key molecular features of BUC and identified two subgroups of molecular characteristics through GSEA (Table S5.1). Our findings aligned with the previous analysis results, showing that the enrichment fraction of luminal differentiation was significantly higher in Cluster A, while that of basal differentiation and immune cell differentiation was significantly higher in Cluster B (Fig. 3D, Table S5.2). This suggests that Cluster A is primarily characterized by luminal differentiation and tumor metabolism, while Cluster B is primarily associated with immune cell infiltration and stromal differentiation.

Subsequently, we examined the characteristics of gene mutations between the two groups in the TCGA database. Our analysis revealed that Cluster A had a significantly higher gain of CNVs on chromosomes 1, 12, 17, and 19, and a higher loss of CNVs on chromosomes 4, 5, and 6, compared to Cluster B (Fig. S7). Furthermore, Cluster A had a significantly higher tumor mutational burden (TMB) than Cluster B. Visualizing the SNVs between the two groups using differential genes, we found that most of the SNVs were missense mutations. The waterfall chart indicated that the mutation frequency of FGFR3 and ELF3 was significantly different between the two groups, with higher mutation frequency of FGFR3 and ELF3 in Cluster A compared to Cluster B (Fig. 3F, G, Table S13.2). To understand the differences in gene mutations of FGFR3 and ELF3 between the two groups, we conducted differential analysis on the gene expression levels of WT and mutated samples of FGFR3 and ELF3, respectively. The results showed that the expression levels of EGFR, FASLG, HAT1, MYC, PANX1 and SLC39A7 were higher in WT FGFR3 samples than that in mutated FGFR3 samples, while the expression levels of GATA3, ID1 and TNFRSF21 were lower in WT FGFR3 samples than that in mutated FGFR3 samples (Fig. S8A). Furthermore, in WT ELF3 samples, the expression level of MYC was higher than that in mutated ELF3 samples, while the expression levels of GATA3 and ID1 were lower in WT ELF3 samples than that in mutated ELF3 samples (Fig. S8B). The co-mutation analysis of necroptosis-related differential genes between the two groups screened out significant co-occurrence and mutually exclusive genes, with MUC16 co-occurring with ERBB3, FREM2, FBN1, FN1, and FRY in Cluster A, whereas MUC16 co-occurred with ASPM and DSP in Cluster B. In Cluster A, FGFR3 was found to co-occur with COL6A3, whereas in Cluster B, this co-occurrence was absent. In addition, ERBB2 was observed to co-occur with DSP in Cluster A, whereas ERBB2 was co-occurring with SACS in Cluster B (Fig. 3E). Since the two necroptotic modules are capable of distinguishing immune patterns, we conducted a simultaneous differential analysis of immune cell infiltration levels between WT and mutated samples of genes including FGFR3 and ELF3. The results showed that samples with different genotypes of FGFR3 and ELF3 had different levels of immune cell infiltration, with significantly lower levels of immune cell infiltration in mutated FGFR3 samples than that in WT FGFR3 samples, including CD8+ T cells, CD4+ T cells, macrophages, neutrophils, and dendritic cells (Fig. S8C). Only dendritic cells showed different levels of immune cell infiltration in WT and mutated samples of ELF3 (Fig. S8D). These findings suggest that the single nucleotide variant of FGFR3 is strongly related to the necroptosis patterns in BUC.

### Characteristics in immune infiltration and checkpoint of the tumor immune microenvironment in necroptosis phenotypes

**Fig. 4:**
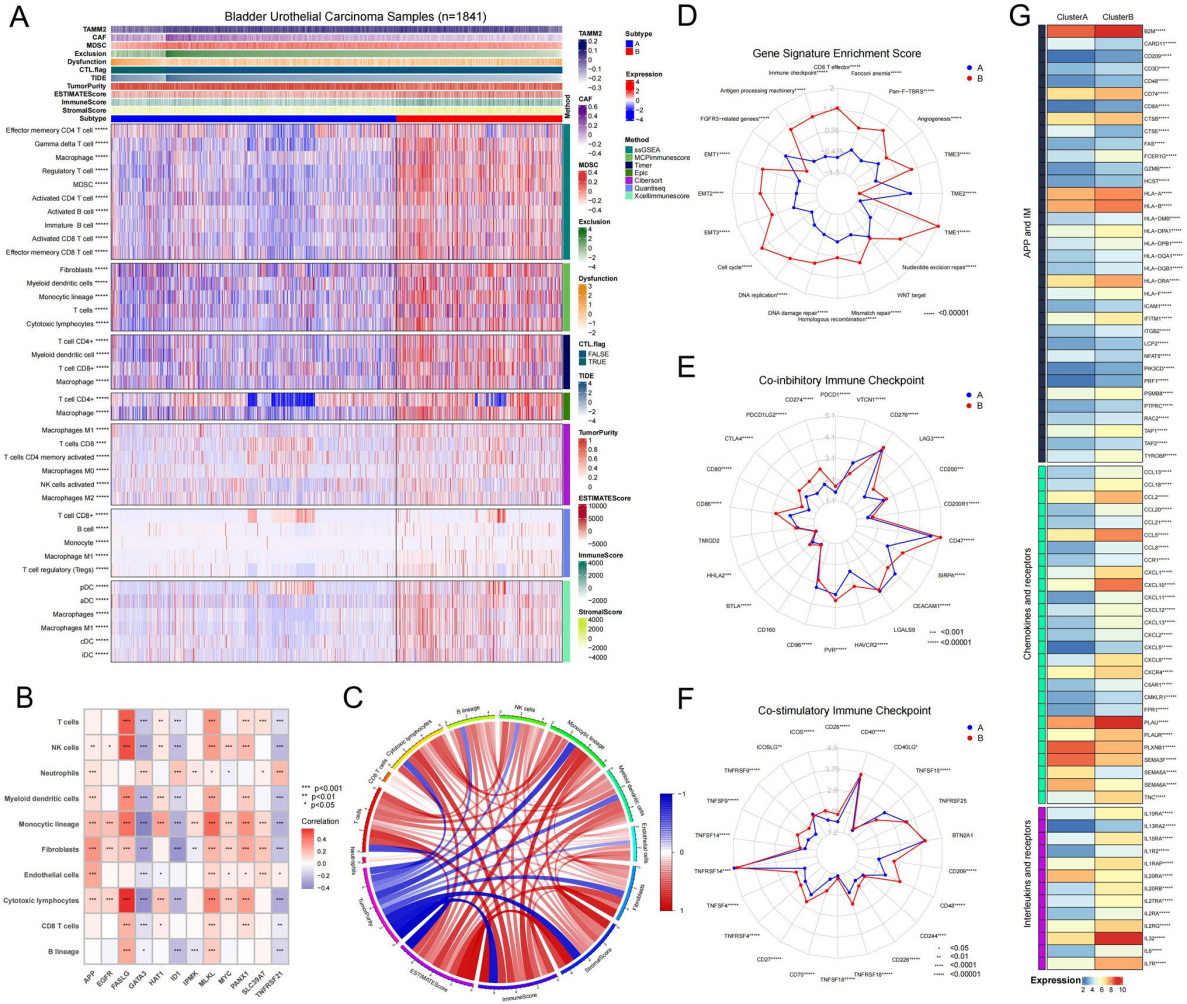
Characteristics in immune infiltration and checkpoint of the tumor immune microenvironment in necroptosis phenotypes. **A** Heatmap shows frequency and immune score of TME infiltrating cells (Kruskal–Wallis test) in the two necroptotic phenotypes. Asterisks indicate P-values (*****P < 0.00001). **B** Heatmap shows correlation between MCP-immunescore and 12 necroptosis genes.** C** Circle map shows connections between tumor immune-infiltrating cells assessed by MCP method. Red represents positive correlation and blue represents negative correlation between different immune cell. The darker line color indicates the stronger correlation between different immune cell, and the larger line width indicates the stronger correlation. **D**–**F** Radar plots show differences in immune checkpoints and core signaling pathways between two necroptotic phenotypes (Wilcoxon test). **G** The heatmap shows variations in mRNA expression of antigen processing and presentation, BCR signaling pathway, TCR signaling pathway, natural killer cells, chemokines, interleukins, and other cytokines in the two necroptosis modules (Wilcoxon test). Asterisks indicate P values (*****P < 0.00001) (Color figure online)

We conducted a detailed investigation into the immune cell infiltration and immune checkpoint characteristics of the two necroptotic modules, as they exhibit different immune patterns. Using seven different scoring methods, including ssGSEA, MCPimmunescore, Timer, Epic, CIBERSORT, Quantiseq, and Xcellimmunescore, we analyzed the immune cell infiltration levels in 1841 BUC samples using R language based on gene expression characteristics. Additionally, we evaluated the samples for ImmuneScore and StromalScore using the 'estimate' R package and for signs of T cell dysfunction and exclusion (TIDE) to predict immunotherapy response (Table S6). Our results revealed that the level of macrophage infiltration in BUC was significantly higher in Cluster B compared to Cluster A, as seen in all seven immune cell scoring methods. Specifically, the infiltration level of tumor-associated macrophages M2 was significantly higher in Cluster B (Fig. 4A). The tumor immune microenvironment also showed a high concentration of myeloid-derived suppressor cells (MDSCs), myeloid dendritic cells, and monocytes in Cluster B (Figs. S9A4, 4A). Furthermore, a substantial accumulation of T cells, including CD4+ and CD8+ T cells, was observed in Cluster B, resulting in significantly higher ImmuneScore and StromalScore compared to Cluster A, despite lower tumor purity (Fig. S9B). The higher scores for T cell dysfunction and exclusion in Cluster B indicated a severe impairment of T cell immunity (Fig. S9A). In conclusion, although Cluster B has a higher T cell infiltration, its T cell function is severely compromised due to a large presence of tumor immunosuppressive cells, such as MDSCs, carcinoma-associated fibroblasts (CAFs), and macrophages. This may contribute to a lower overall survival rate for patients in Cluster B (Fig. 4A). To further understand the impact of necroptosis on the tumor microenvironment, we analyzed the correlation between tumor immune cells and necroptosis genes. Our findings showed positive correlations between FASLG and MLKL and most immune cells using the seven immune scoring methods, while GATA3 ID1 and TNFRSF21 showed negative correlations (Figs. 4B, S10B–C). With the exception of a few immune infiltration cells between each other that showed negative correlation, such as between CD4+ T cells and CD8+ T cells, most tumor immune cells exhibited mutual promotion (Figs. 4C, S9C).

Given the distinct patterns of immune cell infiltration between the two necroptotic modules, we compared the expression differences in key biological processes, cytokines, and immune checkpoints between the two Clusters. We utilized PCA to obtain 17 core biological pathway scores of all samples based on related gene expression (Table S7). Interestingly, the core biological pathway scores, particularly the CD8+ T cell effector scores, were significantly higher in Cluster B than in Cluster A, encompassing processes such as antigen presentation, immune checkpoint regulation, epithelial-mesenchymal transition (EMT), cell cycle, DNA damage repair, tumor immune cell infiltration, and angiogenesis. On the other hand, the score of FGFR3-related genes was higher in Cluster A compared to Cluster B, which could be attributed to the higher rate of FGFR3 mutations in cluster A as depicted in Fig. 3F (Figs. 4D, 3F). Furthermore, by examining the expression of 44 immune checkpoint-related genes, we discovered that the expression of most immune checkpoint genes in Cluster B was significantly higher than that in Cluster A, particularly in the case of co-inhibitory immune checkpoints like PD1, PD-L1, PD-L2, and CTLA4 (Fig. 4E, F). Additionally, the expression levels of cytokine genes revealed the distinct differences between the two necroptotic modules. The cytokines analyzed mainly covered areas such as antigen processing and presentation, BCR signaling, TCR signaling, natural killer cells, chemokines and their receptors, interleukins and their receptors, and other cytokines. Most cytokines were expressed at higher levels in Cluster B than in Cluster A, particularly in the case of the HLA family and the CC and CXC families of chemokines (Figs. 4G, S10A).

### Validation of expression of necroptosis genes in bladder tissues

**Fig. 5:**
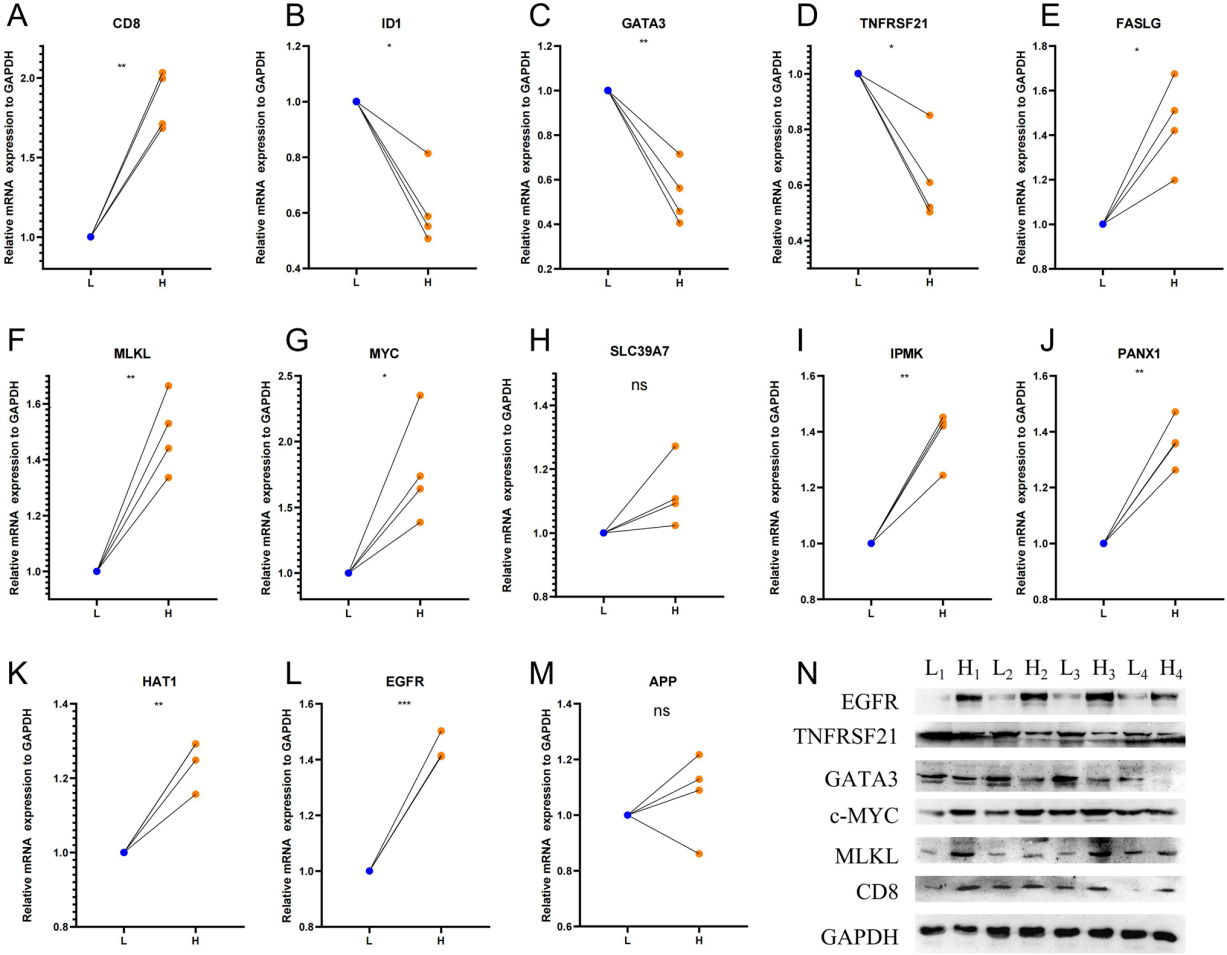
The experimental validation of necroptosis genes in four pairs BUC tissues by RT-qPCR (A-M) and Western Blot (N). The asterisks represent the P value (*P < 0.05; **P < 0.01; ***P < 0.001)

Initially, we detected the mRNA expression of 12 prognostic necroptosis-related genes and CD8 in eight samples (Table S14). Based on the expression level of CD8 mRNA, we divided the samples into two groups: high and low, using the median value as a cutoff. To investigate the relationship between CD8 and the remaining 12 genes, we randomly paired the high CD8 group samples with the low CD8 group samples, resulting in four pairs for comparison. Our findings, presented in Fig. 5, indicate that BUC tissues with high CD8 expression exhibited higher expression levels of genes such as FASLG, MLKL, MYC, IPMK, PANX1, HAT1, and EGFR, and lower expression levels of genes such as ID1, GATA3, and TNFRSF21 compared to those with low CD8 expression. Furthermore, we confirmed the relationship between EGFR, MLKL, TNFRSF21, GATA3, MYC and CD8 in the protein expression levels, and the results were consistent with the above findings (Fig. 5). Regrettably, we did not identify any significant relationship between the expression of CD8 and clinicopathological indicators (Table S15). The correlation between the expression of these genes and CD8 expression was consistent with the correlation between the expression of necroptosis genes and the infiltration level of CD8+ T cells.

### Immune characteristics of key necroptosis genes RIPK3 and MLKL in BUC

We analyzed the association between the CNVs of necroptosis key genes, RIPK3 and MLKL, and the level of immune infiltration of various immune cells using the Tumor Immune Estimation Resource (TIMER) database. The results revealed a correlation between arm-level deletion of MLKL and the infiltration levels of B cells and CD4+ T cells (Fig. S11A). Furthermore, arm-level deletion of RIPK3 was strongly linked to the infiltration levels of CD8+ T cells, CD4+ T cells, neutrophils, and dendritic cells. In contrast, arm-level gain of RIPK3 showed an association with the immune infiltration levels of B cells, CD4+ T cells, neutrophils, and dendritic cells (Fig. S11B).

Next, we evaluated the relationship between the copy number variations of RIPK3 and MLKL, the key genes of necroptosis in pan-cancer, and the levels of immune infiltration of different immune cells using the TIMER database. Our findings showed that MLKL was positively correlated with the expression of immune checkpoints such as PD1, PD-L1, PD-L2, and CTLA4 in most cancer types, except for ICOSLG, CD200, CEACAM1, HHLA2, and VTCN1 in BUC. Additionally, MLKL expression was positively correlated with the level of all immune cell infiltration in most cancer types (Fig. S11C, Table S8.1 and Fig. S11D, Table S8.2). On the other hand, the expression of RIPK3 showed a positive correlation with most immune checkpoints, only in nine cancer types such as KIRC, KIRP, PRAD, SARC, SKCM, and TCGT. However, in BUC, RIPK3 expression was not significantly related to important immune co-inhibitory checkpoints like PD1, PD-L1, PD-L2, and CTLA4 (Fig. S11E, Table S8.3). The expression of RIPK3 was positively correlated with the level of immune cell infiltration in cancers like ACC and GBM, but in BUC, it was not strongly correlated with the level of immune cell infiltration (Fig. S11F, Table S8.4). In conclusion, our findings showed that MLKL promotes the infiltration of various immune cells and co-expression of immune checkpoints, especially in BUC, while the type of copy number variation of RIPK3 has a significant effect on the level of immune cell infiltration.

### Clinical and immunological characteristics of NecroScore

**Fig. 6:**
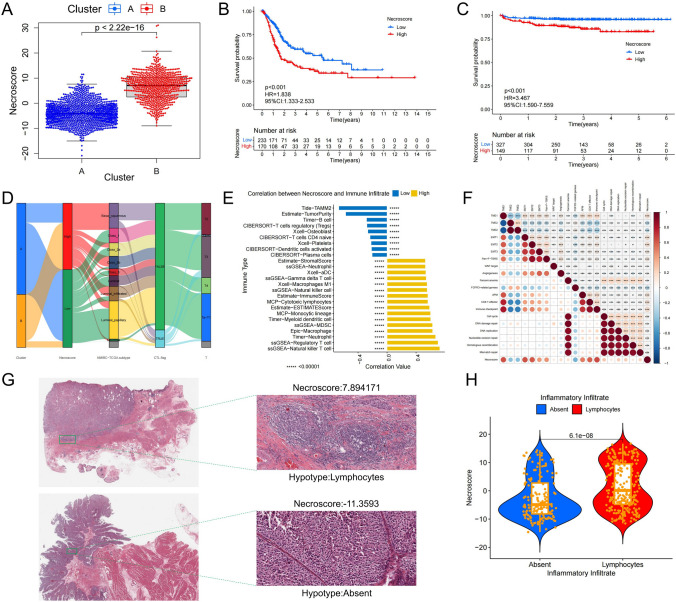
Clinical significance and immune characteristics of NecroScore. **A** The Kruskal–Wallis test showed NecroScore scores for two necroptotic phenotypes. **B** Kaplan–Meier curves show significant differences in overall survival between high or low NecroScore in the TCGA database. **C** Kaplan–Meier curves show significant differences in disease progression survival between high or low NecroScore in the E-MTAB-4321 database. **D** Sankey diagram shows correlation between NecroScore and molecular subtypes, T cell functional status in BUC. **E** Histogram shows the relationship between NecroScore and level of immune cell infiltration. **F** The bubble chart shows the correlation between NecroScore and TME scores, core signaling pathways. Brown bubbles indicate positive correlations, and blue indicate negative correlations. Color depth and bubble size indicate the strength of the correlation (***P < 0.001). **G** Images represent pathological HE staining changes between high and low NecroScore groups in the TCGA database. **H** The Wilcoxon test measures NecroScore differences between pathological immunophenotypes. The points represent the NecroScore value for each sample, with the upper and lower ends representing the interquartile range for that value. Lines in boxes represent median values; black dots represent outliers (Color figure online)

To predict the prognosis and immunotherapy effect of BUC patients, we created a PCA model named NecroScore using the differential genes between two necroptosis modules that showed significant survival differences and immune correlations. We applied the NecroScore scoring formula to 1841 BUC samples based on gene expression data (Table S10). Our analysis revealed that the NecroScore of Cluster B was significantly higher than that of Cluster A (Fig. 6A). Of the 12 necroptosis genes that make up the necroptosis module, 11 genes were found to be strongly correlated with the NecroScore (Fig. S12A). The genes PANX1, MYC, FASLG, and MLKL had a positive correlation with NecroScore, while ID1, TNFRSF21, and GATA3 were negatively correlated. The gene SCL39A7 had no significant correlation with NecroScore. Furthermore, our investigation into the relationship between NecroScore and the clinical molecular typing of BUC showed that as the NecroScore increased, the molecular typing of BUC tended to shift from non-muscle-related molecular typing and luminal molecular typing to basal molecular typing (Fig. S12B, Table S13.4). The overall survival rates of the BUC samples with high and low NecroScore scores were significantly different in the TCGA and GEO datasets and the progression survival rates in the EMTAB dataset also differed significantly (Figs. 6B, C, S14A).

The TCGA dataset reveals similarities between the SNV mutations in the high and low NecroScore groups and the necroptosis module, as evidenced by the waterfall plot and correlation pheatmap of gene co-mutation (Fig. S13A–C, Table S13.3). In particular, the high NecroScore group is comparable to Cluster B, while the low NecroScore group is comparable to Cluster A in the TCGA mutation data (Tables S13.2, S13.3). Furthermore, the molecular typing of BUC shows improved accuracy in differentiating the high and low NecroScore groups. The low NecroScore group is primarily comprised of class_1, class_2a and class_3 molecular typing in NMIBC_subtype, while the high NecroScore group primarily consists of basal-related subtypes in MIBC_subtype, MDA_subtype, TCGA_subtype, Lund_subtype, UNC_subtype, and Baylor_subtype. The luminal-like subtype and other subtypes are predominantly found in the low NecroScore group. Of utmost significance, the NecroScore can effectively identify patients with defective toxic T lymphocytes (CTL.flag). Furthermore, a higher level of defective cytotoxic T lymphocyte infiltration may be one of the reasons for the lower survival rate observed among patients in the high NecroScore group. In the CIT_subtype, the MC7 subtype is primarily found in the high NecroScore group, while the MC1 subtype is mostly located in the low NecroScore group (Figs. 6D, S13D, Table S13.4).

To further evaluate the role of NecroScore, we investigated its ability to predict immune cell infiltration levels and its relationship with core biological pathways. In 1841 BUC samples, it was found that all co-inhibitory and co-stimulatory immune checkpoints, except for TMIGD2, CD160, ICOSLG, BTN2A1, and TNFRSF25, were significantly different between the high and low NecroScore groups. The high NecroScore group exhibited significantly higher expression of co-suppressive immune checkpoints, such as PD1, PD-L1, PD-L2, and CTLA4, compared to the low NecroScore group (Fig. S12C–D). The correlation analysis between NecroScore and the level of immune cell infiltration revealed that most immune cell infiltration levels increased with the increase of NecroScore, particularly in natural killer T cells, regulatory T cells, neutrophils, and macrophages. In contrast, there was a strong negative correlation between NecroScore and tumor purity (Figs. 6E, S13E). NecroScore had a positive effect on most core pathways and showed a positive correlation with CD8 T effector, immune checkpoint, and EMT. The negative correlation between NecroScore and FGFR3-related genes was consistent with the previously observed low mutation rate of FGFR3 in the high NecroScore group (Figs. 6F, S13B). To validate the role of NecroScore in predicting immune infiltration levels, we selected BUC HE samples from the TCGA database. The results showed that HE samples with high levels of lymphocyte infiltration also had high NecroScore, while HE samples lacking immune cell infiltration had low NecroScore (Fig. 6G, H). This confirms the efficacy of NecroScore as a model for analyzing immune infiltration levels and its accuracy in predicting important immune checkpoints such as PD1 and PD-L1.

### Prediction of prognosis of BUC patients by NecroScore

**Fig. 7:**
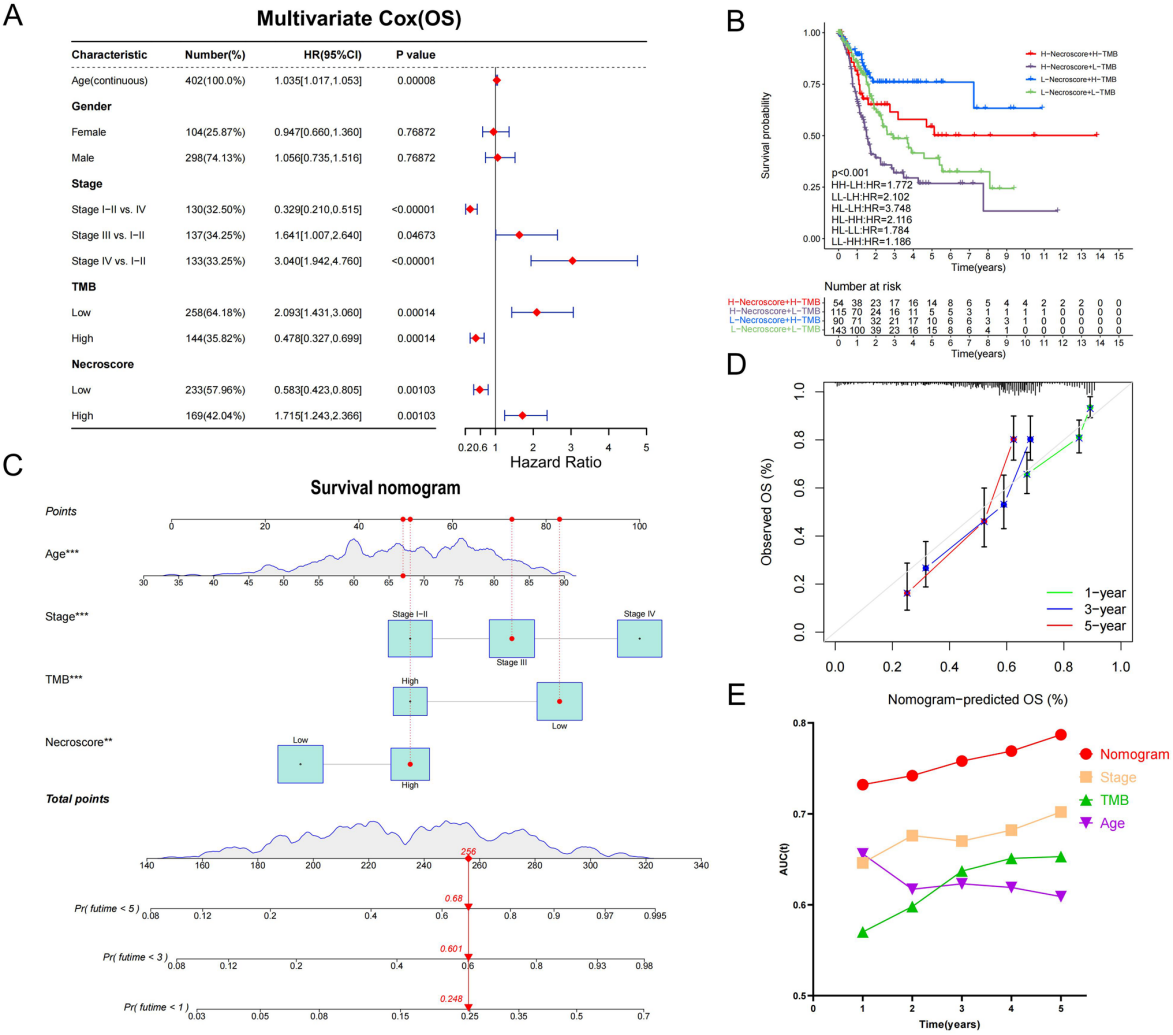
Prediction of prognosis of BUC patients by NecroScore.** A** We performed a multivariate COX analysis of the TCGA cohort using five metrics, Age Gender Stage TMB and NecroScore. **B** Kaplan–Meier curve analysis of survival for patients in the TCGA cohort using NecroScore and TMB. **C** Use the "regplot" package to score four prognostic indicators (Age Stage TMB NecroScore) to establish a new prognostic model. Survival nomogram shows scoring and prognostic results for the first patient in the TCGA BUC cohort.** D** Calibration curve plot showing the relationship between the patient prognosis predicted by the survival nomogram and the actual patient prognosis. The closer the curve is to the central axis, the more accurate the prediction results. **E** We tested model sensitivity and specificity using ROC curves for Nomogram, Stage, TMB, and Age. The abscissa represents survival time, and the ordinate represents the area under the curve (AUC). The higher AUC value indicates the higher the authenticity of the detection method

Based on the results of our analysis, we found that the prognosis of BUC patients in the high and low NecroScore groups was significantly different. To determine the impact of NecroScore on BUC patient prognosis, we performed a multivariate Cox regression analysis. Five factors were considered in the analysis, including Age, Gender, Grade, TMB, and NecroScore. Of these, Age, Grade, TMB, and NecroScore had a significant impact on BUC patient survival rate (Fig. 7A). It was observed that TMB was positively associated with survival in BUC patients, unlike NecroScore and Stage (Fig. S14B). To further understand the impact of NecroScore and TMB, we divided the TCGA BUC dataset into four groups based on these two factors and found that there were significant differences in survival rate among the groups, with the highest survival rate in the low-NecroScore-high-TMB group and the lowest in the high-NecroScore-low-TMB group (Fig. 7B). To predict the prognosis of BUC patients, we developed a comprehensive evaluation model that took into account Age, Stage, TMB, and NecroScore (Fig. 7C). This model was effective in predicting 1-year and 3-year survival rate but less accurate in predicting 5-year survival rate [Fig. 7D). The model was verified by the ROC curve, which showed that the AUC value increased with increased survival time. However, the use of a single index, such as Stage or TMB, is not considered reliable enough for predicting BUC patient prognosis. To evaluate the prediction accuracy of the model, we used the C index and found that the evaluation model is reliable (Figs. 7E, S14C–H).

### NecroScore predicts sensitivity of BUC patients to anti-tumor therapy

Based on the drug response data from the public pharmacogenomics databases, Genomics of Drug Sensitivity in Cancer (GDSC) and Therapeutics Response Portal (CTRP), we analyzed the relationship between necroptosis-related molecules and the efficacy of clinical treatment for BUC. The genomic drug resistance analysis data were obtained from GDSC/CTRP IC50 drug data, and the correlation between gene expression and drug efficacy was analyzed using the Spearman correlation method. A negative correlation indicates that high gene expression makes a patient more sensitive to the drug, while a positive correlation indicates the opposite. Our results demonstrate that most genes exhibit high resistance to these drugs, but EGFR, TNFRSF21, and ID1 display significant synergistic effects against ERBB therapeutic drugs, including lapatinib, gefitinib, erlotinib, cetuximab and afatinib. Additionally, we observed synergistic effects of multiple genes on drugs such as 17-AAG (Tanespimycin, an Hsp90 inhibitor), trametinib (a MEK inhibitor), and docetaxel (a paclitaxel antineoplastic drug). Patients with high MLKL expression were found to be sensitive to 17-AAG, Trametinib, RDEA119 (Refametinib, MEK inhibitors), and CI-1040 (MEK inhibitors) (Fig. S15A). To our surprise, nearly all antineoplastic drugs in the CTRP database were found to be strongly sensitive to patients with high MYC expression (Fig. S15B). To gain a more comprehensive understanding of the relationship between necroptosis-related molecules and antitumor drug sensitivity, we analyzed the correlation between gene expression and drug sensitivity in various cancer cell lines using the CellMiner database. A positive correlation implies that a drug is more sensitive to higher gene expression. The results revealed that several drugs had synergistic effects on necroptosis genes, including AMG-900 (pan-Aurora kinase inhibitor), BLU-667 (Pralsetinib, RET inhibitor), BOS-172722 (MPS1 inhibitor), CH5132799 (PI3K inhibitor), Dexrazoxane (cardioprotective), IDH-C227 (IDH1 inhibitor), Irofulven (DNA alkylating agents), P529 (Palomid 529, mTORC1 and mTORC2 complex inhibitors), and PQR530 (PI3K/mTORC1/2 dual inhibitor) etc. Drugs such as AMG900, BOS-172722, and Dexrazoxane were found to be strongly sensitive to several necroptosis-related molecules, including MLKL (Fig. S15C, Table S11).

The significance of necroptosis-related molecules in chemotherapy prompted us to evaluate the accuracy of NecroScore in predicting chemotherapy outcomes in BUC patients. We examined gene expression differences between high and low NecroScore groups of BUC with common drug targets, including Chemotherapy, Immune-therapy, ERBB-therapy, FGFR-therapy, and antiangiogenic-therapy. The results showed that most common drug targets were highly expressed in the high NecroScore group, except for Afatinib (ERBB2, ERBB4), Trastuzumab (ERBB2), Lapatinib (ERBB2), Infigratinib (FGFR2, FGFR3), and Sorafenib (BRAF, RAF1) (Fig. S16A). To further validate the therapeutic effect of chemotherapy drugs in BUC patients, we utilized the GDSC database and the “pRRophetic” R package to predict the response of common chemotherapy drugs in patients with high and low NecroScore. The results showed that the IC50 values of cisplatin, doxorubicin, gemcitabine, and other chemotherapeutics were lower in the highNecroScore group, indicating that patients with high NecroScore may be more sensitive to these drugs (Fig. S16B–I).

### NecroScore predicts immunotherapy response in BUC patients

**Fig. 8:**
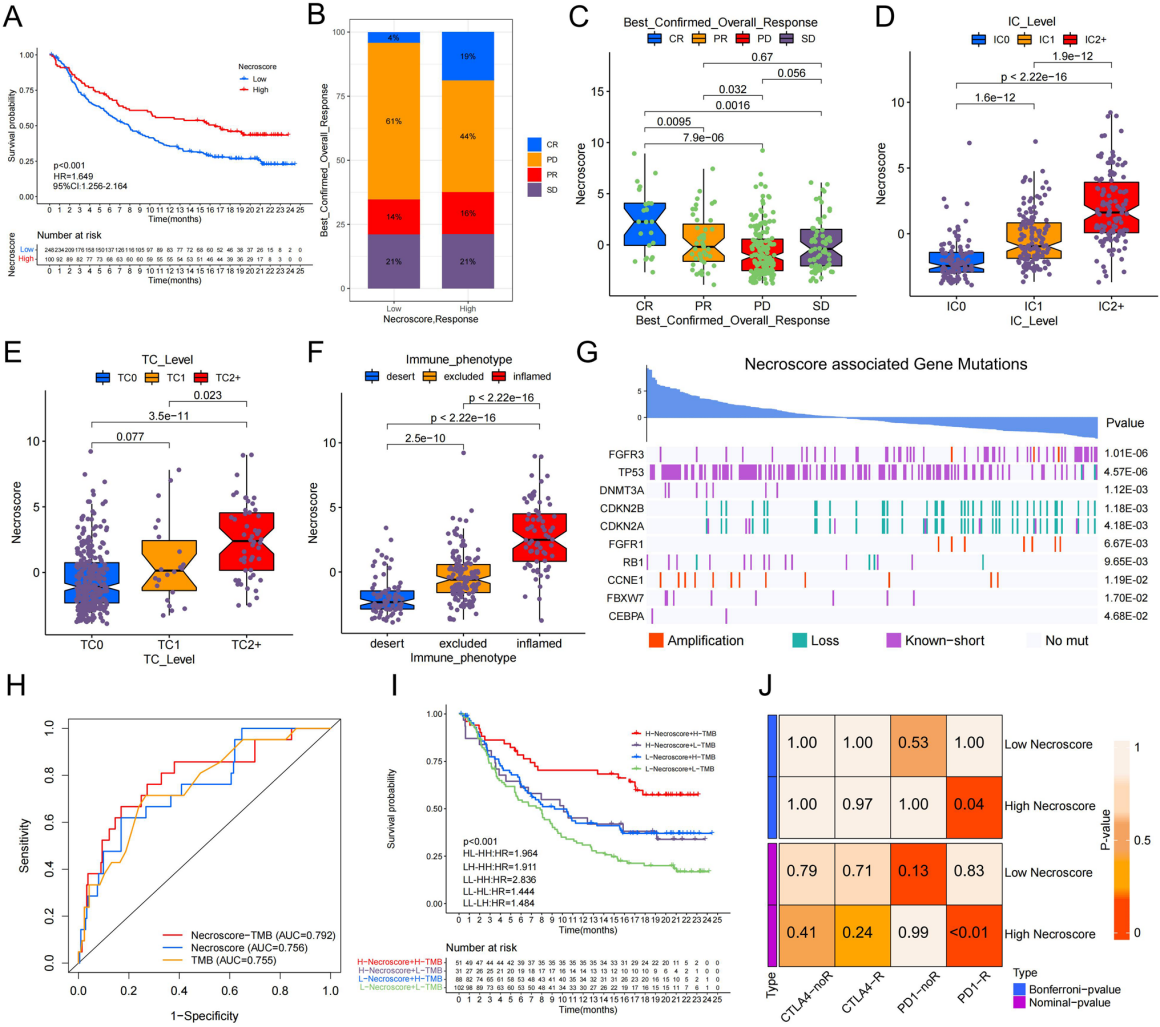
NecroScore predicts immunotherapy response in BUC patients.** A** Kaplan–Meier curves show a significant difference in survival between the high and low NecroScore groups of the IMvigor210 dataset.** B** The stacked histograms show differences in anti-PD-L1 responses between high and low NecroScore. CR (complete response), PR (partial response), SD (stable disease), PD (progressive disease). **C** Boxplots show the anti-PD-L1 reactivity of NecroScore variables, using the Wilcoxon test between pairs. The upper and lower bounds represent the interquartile range of values, and the lines in the boxes represent the median. **D** and **E** Boxplots show NecroScore variables for immune cells (D) and tumor cells (E) with different PD-L1 expression levels, using the Kruskal–Wallis test between pairs. The upper and lower ends represent the interquartile range of the value, and the lines in boxes indicate the median. **F** NecroScore was tested in three immune subtypes using the Kruskal–Wallis test. The upper and lower bounds represent the interquartile range of values, and the lines in boxes indicate the median.** G** Correlation between NecroScore and gene copy number variation in BUC. Histograms represent NecroScore, with each column representing one patient, red for copy number amplification, green for copy number loss, purple for copy number short mutation, and grey for no mutation. **H** ROC curve analysis of NecroScore and TMB predictive value of CR in patients with anti-PD-L1 immunotherapy. **I** Kaplan–Meier curve analysis of survival in patients receiving anti-PD-L1 immunotherapy using NecroScore and TMB. **J** Probabilities of anti-PD1 and anti-CTLA4 immunotherapy responses in high and low NecroScore groups were predicted using the submap algorithm. The high NecroScore group may have a better response on anti-PD-1 treatment (Bonferroni-corrected P = 0.04). R: Respond, noR: no Response

We conducted NecroScore analysis on patients in the IMvigor210 cohort and divided them into high and low NecroScore groups. The results showed that patients in the high NecroScore group had better outcomes after anti-PD-L1 treatment compared to those in the low NecroScore group. (Figs. 8A, S17A, B). Our analysis revealed a significant correlation between NecroScore and the effectiveness of anti-PD-L1 treatment, with a higher proportion of complete responses (CR) observed in the high NecroScore group compared to the low NecroScore group (Fig. 8B, C). Interestingly, we discovered that as NecroScore increased, the proportion of positive cells (PD-L1 expression ≥ 1%) also increased in both immune cells and tumor cells in the IMvigor210 cohort, especially in the TC2+ (TC ≥ 5%) and IC2+ (IC ≥ 5%) ratios, which were significantly higher in the high NecroScore group compared to the low NecroScore group (Figs. 8D, E, S17C, D). Furthermore, the expression levels of PD1 and PD-L1 were significantly higher in the high NecroScore group compared to the low NecroScore group (Fig. S17F, G). The immune phenotype of BUC patients in the high NecroScore group was more inclined towards an inflamed phenotype, while the low NecroScore group was more inclined towards a desert phenotype (Figs. 8F, S17E). Our evaluation of the relationship between gene copy number alterations and NecroScore in the IMvigor210 cohort showed that short variants (< 49 bp long) of FGFR3 were more prevalent in patients with lower NecroScore, while the opposite was true for short variants of TP53. Deletions of the cell cycle-related genes CDKN2A and CDKN2B were the main form of mutation (Fig. 8G).

Previous studies have shown that TMB is a key factor in determining the effectiveness of immunotherapy [19]. By combining NecroScore with TMB, we found that the prediction of CR improved significantly compared to using a single factor (Fig. 8H). The combination of NecroScore and TMB provided a more accurate prognostic prediction compared to using a single factor in the IMvigor210 cohort (Figs. 8, I, S17H). Additionally, using the submap module, we predicted the immunotherapy response to anti-PD1 and anti-CTLA4 in both the high and low NecroScore groups. Results showed that the high NecroScore group had a better response to anti-PD1 treatment (Fig. 8J). Our NecroScore model also demonstrated high accuracy in predicting the immunotherapy effects of anti-PD-L1 and anti-PD1.

### RIPK3 and MLKL can regulate the tumor growth rate and the degree of tumor immune cell infiltration in vivo

**Fig. 9:**
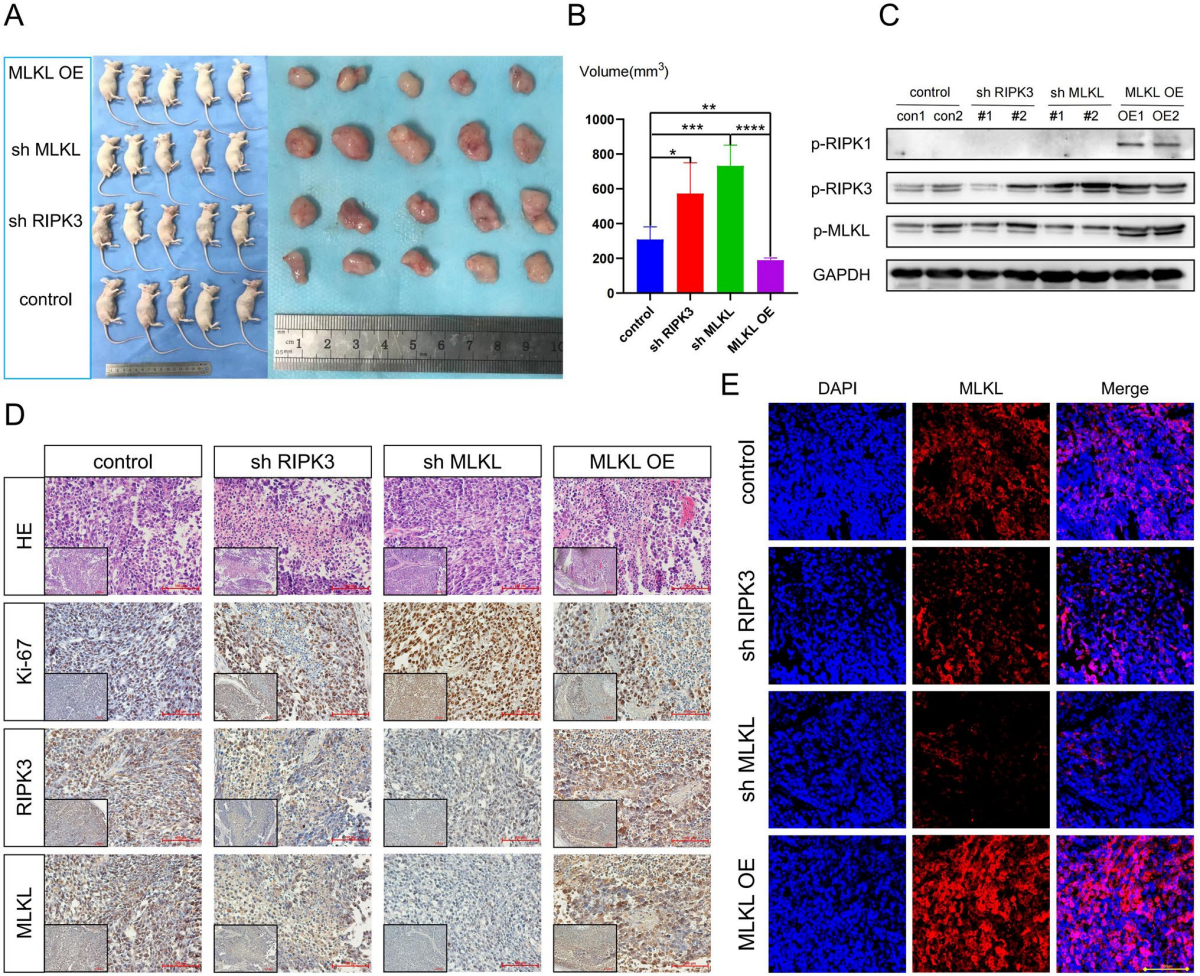
Effects of RIPK3 and MLKL on tumor growth and tumor immune cell infiltration in vivo.** A** Four groups of cells constructed using T24 cell lines were implanted subcutaneously in nude mice, including negative control cells, sh-RIPK3 and sh-MLKL cells, and MLKL-OE cells. **B** The tumor volume was calculated using V = $\frac{\pi }{6}$ LW^2^ (L = tumor length, W = tumor width), and was compared between the two groups using T test (*P < 0.05, **P < 0.01, ***P < 0.001, ****P < 0.0001). **C-E** Four groups of subcutaneous tumors of BUC were analyzed by WB, HE (hematoxylin–eosin staining), IHC (immunohistochemistry), IF (immunofluorescence) assay

We successfully established a subcutaneous BUC model in nude mice using control, shRIPK3, shMLKL, and overexpressed MLKL T24 cells. The growth rate of subcutaneous BUC tumors with knockout of RIPK3 and MLKL was significantly faster compared to the ordinary T24 cell-transfected nude mice. On the other hand, the growth rate of subcutaneous BUC tumors with overexpression of MLKL was the reverse (Fig. 9A, B). Our results showed that the expression of the corresponding phosphorylated proteins of RIPK3 and MLKL decreased in the knockout groups, while the expression of phosphorylated MLKL increased in the overexpressed group (Fig. 9C–E). Furthermore, the proliferation capacity of subcutaneous BUC tumors with overexpressed MLKL was significantly lower, while that with knockout of RIPK3 and MLKL improved (Fig. 9D). The subcutaneous tumor tissues with MLKL overexpression displayed a large number of neutrophil infiltrations, while the subcutaneous tumor tissues with knockout MLKL had virtually no neutrophil infiltration. This is consistent with previous findings that higher RNA expression of MLKL leads to more neutrophil infiltration (Figs. 9D, S11D).

## Discussion

The impact of necroptosis on tumor immunity is not fully understood, despite evidence suggesting that it plays a crucial role in inducing antitumor immunity [20]. In this study, we analyzed the genetic and transcriptomic diversity of 67 necroptosis genes in normal and tumor tissues across 33 cancer types. Our findings suggest that imbalances in the expression of necroptosis molecules may be linked to gene methylation modifications, genetic mutations, and key signaling pathways such as EMT and the cell cycle. By using univariate COX regression analysis, we screened 12 necroptosis genes that have a significant impact on prognosis and found that they play a crucial role in the immune subtypes of BUC, particularly MLKL. Through a comprehensive analysis of 1841 BUC samples, we identified two subtypes of necroptosis and observed significant differences in clinical characteristics, gene mutation patterns, and immune cell infiltration between the two clusters. While previous research has shown that RIPK3-dependent necroptosis of pancreatic cancer cells leads to the recruitment of immunosuppressive cells such as MDSCs to create an immunosuppressive tumor microenvironment [10], our study highlights a strong association between necroptosis subtypes and the tumor immune microenvironment in BUC.

The validation of transcriptome expression in different subtypes of necroptosis has been shown to be crucial for molecular typing of BUC and understanding its impact on immune-related biological pathways. Our study revealed an association between necroptosis and BUC molecular classification: the low necroptosis group (Cluster A) was found to be related to the luminal-like subtype, while the high necroptosis group (Cluster B) was linked to the basal-like subtype. Previous research has shown that necroptosis signaling pathways play a role in the immunosuppressive microenvironment of tumors in diseases such as pancreatic ductal adenocarcinoma (PDAC) and melanoma, leading to immune tolerance and resistance to immunotherapy [10, 11, 21]. Our study found that necroptosis genes are critical for promoting tumor immune cell infiltration and leading to a worse prognosis in BUC. Additionally, our research validated a strong correlation between the high necroptosis group and the hypoxia signaling pathway in BUC. While the relationship between hypoxia and necroptosis has been found in non-neoplastic diseases such as retinal neovascular diseases, hepatic injury, and ischemic heart disease [22–24], this study extends these findings to BUC. Previous studies have also shown that SMAC mimetics, which bind and degrade cIAPs and induce necroptosis, can promote antitumor immune responses [25]. Moreover, SMAC mimetics can collaborate with immune checkpoint inhibitors to maintain a durable therapeutic response for glioblastoma [26, 27]. Our study found significant differences in immune checkpoint expression, particularly PD1 and PD-L1, between different necroptosis patterns. The high necroptosis modules (Cluster B) were also found to have higher enrichment in core tumor progression biological processes such as CD8 T cell effector and EMT. As a result, the combination therapy of necroptosis-inducing SMAC mimetics and immune checkpoint inhibitors has great potential for BUC with high necroptosis modules.

The frequency of FGFR3 alterations in BUC is generally higher in non-muscle-invasive cases compared to muscle-invasive cases, with alterations being associated with lower grades and stages [28]. The frequency of FGFR3 alterations was significantly higher in non-muscle-invasive BUC (49%) than in muscle-invasive BUC (10–14%) [28–30]. In this study, we analyzed invasive BUCs from TCGA and identified two necroptotic modules: high and low. We found that the high necroptotic module had a significantly lower rate of FGFR3 alterations (6%) compared to the low necroptotic module (19%). Our results showed a clear relationship between necroptosis gene expression and FGFR3 mutations, contributing to a more accurate classification of FGFR3 alterations in muscle-invasive bladder carcinomas (MIBC). The luminal papillary subtype of urothelial carcinoma is associated with FGFR3 genetic mutations [31, 32], which is consistent with the high rate of FGFR3 alterations observed in the low necroptotic module. Despite FGFR3 inhibiting key components of the adaptive immune response, including lymphocyte infiltration and CD8A T-cell expression [33], our findings revealed that WT FGFR3 in BUC tissues promote the infiltration of tumor immune cells such as CD8+ T cells, macrophages, and dendritic cells, compared to mutated FGFR3.

Subsequently, we examined the relationship between the two core necroptosis-related genes, RIPK3 and MLKL, and tumor immune infiltration across different types of cancer. Surprisingly, in the majority of cancers, both RIPK3 and MLKL promoted tumor immune cell infiltration, particularly in BUC where MLKL increased the infiltration of neutrophils, which supports previous findings that necroptosis can boost antitumor immunity [34]. Conventional therapeutic drugs primarily function by inducing tumor cell death through apoptosis, but this approach often lacks efficacy due to resistance to drugs and scattered immune responses. Our study found that high expression of MLKL significantly inhibited the growth of subcutaneous tumors in BUC, which could be a promising therapeutic target. There are also several therapeutic agents, including doxorubicin and cisplatin, in classical chemotherapy drugs, that can trigger necroptosis in tumor cells when combined with other modulators [35]. Our study discovered that several antitumor drugs, such as 17-AAG, AMG900, and BOS-172722, were highly effective in patients with high expression of cross-necroptosis genes, particularly MLKL, which may overcome resistance to classical chemotherapy and enhance the anti-tumor effect.

In view of the crucial role of necroptosis in regulating BUC immunity and the heterogeneous necroptosis phenotype among BUC patients, it is essential to classify the expression of necroptosis regulators in these patients. To achieve this, we developed a scoring system, NecroScore, to evaluate necroptosis patterns in BUC patients. Our findings confirmed that NecroScore provides a reliable and comprehensive assessment of BUC-related molecules, with a high NecroScore indicating basal-like differentiation and low FGFR3 alterations. Validation of NecroScore on the prognosis of BUC patients demonstrated its reliability, and we constructed a prognostic model incorporating NecroScore to accurately predict the outcome of BUC patients. The NecroScore was positively correlated with tumor immune cell infiltration levels including MDSCs, M2 macrophages and defective toxic T lymphocytes etc., and a higher NecroScore was closely linked to higher expression of co-inhibitory immune checkpoints such as PD1 and PD-L1. The validation of the IMvigor210 cohort showed that NecroScore can predict the treatment effect of anti-PD-L1, especially in patients who achieve complete response. Furthermore, the combination of TMB and NecroScore provided a better prediction of anti-PD-L1 treatment response. Our study also demonstrated that high NecroScore scores had higher sensitivity to classical BUC chemotherapy drugs, such as cisplatin and gemcitabine. In conclusion, NecroScore can be used to assess the expression patterns of necroptosis-related molecules and corresponding immune cell infiltration characteristics in BUC patients, thereby guiding their chemotherapy. Furthermore, NecroScore plays a critical role in predicting the survival rate of BUC patients and the therapeutic effect of immune checkpoint inhibitors such as anti-PD-L1 in advanced and metastatic BUC. A recent study has shown that administering first-line maintenance Avelumab following initial platinum-containing chemotherapy without PD can significantly increase the survival rate of patients with bladder urothelial carcinoma [13]. As a predictor of chemotherapeutic drug sensitivity and anti-PD-L1 treatment effect, NecroScore can be utilized to guide treatment regimens for advanced and metastatic BUC, particularly the combination of PD-L1 inhibitors and platinum chemotherapy.

Although we attempted to study the heterogeneity of necroptosis in as many samples as possible, this was a cross-cohort retrospective study that had limitations such as batch effects. Despite NecroScore's high accuracy in predicting immune effects, further validation is needed through the use of patient data from a multicenter clinical cohort. To confirm the role of RIPK3 and MLKL in promoting tumor immune cell infiltration in BUC, large-scale protein analysis is necessary. Our study suggests that some anti-tumor drugs that are highly sensitive to patients with high expression of necroptosis genes could be new and promising therapeutic measures, especially for those with advanced or chemo-resistant BUC. However, more research is needed to fully understand and explore the potential of these findings.

In conclusion, the combination therapy of necroptosis-inducing SMAC mimetics and immune checkpoint inhibitors exhibits immense potential for bladder urothelial carcinoma patients with high necroptosis modules. It is worth noting that the high necroptotic module was associated with a significantly lower rate of FGFR3 alterations (6%) compared to the low necroptotic module (19%). NecroScore, as a scoring tool, displayed a positive correlation with the infiltration levels of tumor immune cells, including MDSCs, M2 macrophages, and defective toxic T lymphocytes, among others. A higher NecroScore was also found to be closely linked to higher expression of co-inhibitory immune checkpoints such as PD1 and PD-L1. As a predictor of chemotherapeutic drug sensitivity and anti-PD-L1 treatment effect, NecroScore can be utilized to guide treatment regimens for advanced and metastatic bladder urothelial carcinoma patients, particularly the combination of PD-L1 inhibitors and platinum chemotherapy. We hope to obtain more clinical data to support our study findings, and NecroScore can play a vital role in guiding the chemotherapy and immunotherapy of bladder urothelial carcinoma patients in the future.

## Supplementary Information

Below is the link to the electronic supplementary material.

Supplementary file1 (JPG 667 KB)

Supplementary file2 (JPG 1996 KB)

Supplementary file3 (JPG 2057 KB)

Supplementary file4 (JPG 1675 KB)

Supplementary file5 (JPG 875 KB)

Supplementary file6 (JPG 1027 KB)

Supplementary file7 (JPG 740 KB)

Supplementary file8 (JPG 838 KB)

Supplementary file9 (JPG 1451 KB)

Supplementary file10 (JPG 1731 KB)

Supplementary file11 (JPG 3637 KB)

Supplementary file12 (JPG 1193 KB)

Supplementary file13 (JPG 1980 KB)

Supplementary file14 (JPG 763 KB)

Supplementary file15 (JPG 1757 KB)

Supplementary file16 (JPG 1749 KB)

Supplementary file17 (JPG 521 KB)

Supplementary file18 (DOC 76 KB)

Supplementary file19 (XLSX 4884 KB)

Supplementary file20 (XLSX 46 KB)

Supplementary file21 (DOCX 18 KB)

Supplementary file22 (DOC 225 KB)

## Data Availability

All public datasets used in this research are all available from public and open source databases. For detailed datasets information and sources, please see the Materials and Methods section.

## References

[1] Sung H, Ferlay J, Siegel RL, Laversanne M, Soerjomataram I, Jemal A (2021). Global cancer statistics 2020: GLOBOCAN estimates of incidence and mortality worldwide for 36 cancers in 185 countries. CA Cancer J Clin.

[2] Liu S, Chen X, Lin T (2022). Emerging strategies for the improvement of chemotherapy in bladder cancer: current knowledge and future perspectives. J Adv Res.

[3] Flaig TW, Spiess PE, Agarwal N, Bangs R, Boorjian SA, Buyyounouski MK (2018). NCCN guidelines insights: bladder cancer, version 5.2018. J Natl Compr Canc Netw.

[4] Lenis AT, Lec PM, Chamie K, Mshs MD (2020). Bladder cancer: a review. JAMA.

[5] Tran L, Xiao J-F, Agarwal N, Duex JE, Theodorescu D (2021). Advances in bladder cancer biology and therapy. Nat Rev Cancer.

[6] Cathomas R, Lorch A, Bruins HM, Compérat EM, Cowan NC, Efstathiou JA (2021). Updated European Association of Urology guidelines on metastatic urothelial carcinoma. Eur Urol.

[7] Gao W, Wang X, Zhou Y, Wang X, Yu Y (2022). Autophagy, ferroptosis, pyroptosis, and necroptosis in tumor immunotherapy. Signal Transduct Target Ther.

[8] Galluzzi L, Kepp O, Krautwald S, Kroemer G, Linkermann A (2014). Molecular mechanisms of regulated necrosis. Semin Cell Dev Biol.

[9] Tang R, Xu J, Zhang B, Liu J, Liang C, Hua J (2020). Ferroptosis, necroptosis, and pyroptosis in anticancer immunity. J Hematol Oncol.

[10] Seifert L, Werba G, Tiwari S, Giao Ly NN, Alothman S, Alqunaibit D (2016). The necrosome promotes pancreatic oncogenesis via CXCL1 and Mincle-induced immune suppression. Nature.

[11] Wang W, Marinis JM, Beal AM, Savadkar S, Wu Y, Khan M (2018). RIP1 kinase drives macrophage-mediated adaptive immune tolerance in pancreatic cancer. Cancer Cell.

[12] Wang Y, Hao F, Nan Y, Qu L, Na W, Jia C (2018). PKM2 inhibitor shikonin overcomes the cisplatin resistance in bladder cancer by inducing necroptosis. Int J Biol Sci.

[13] Grivas P, Agarwal N, Pal S, Kalebasty AR, Sridhar SS, Smith J (2021). Avelumab first-line maintenance in locally advanced or metastatic urothelial carcinoma: applying clinical trial findings to clinical practice. Cancer Treat Rev.

[14] Liu C-J, Hu F-F, Xia M-X, Han L, Zhang Q, Guo A-Y (2018). GSCALite: a web server for gene set cancer analysis. Bioinformatics.

[15] Qin X, Li J, Hu W, Yang J (2020). Machine learning K-means clustering algorithm for interpolative separable density fitting to accelerate hybrid functional calculations with numerical atomic orbitals. J Phys Chem A.

[16] Wilkerson MD, Hayes DN (2010). ConsensusClusterPlus: a class discovery tool with confidence assessments and item tracking. Bioinformatics.

[17] Mariathasan S, Turley SJ, Nickles D, Castiglioni A, Yuen K, Wang Y (2018). TGFβ attenuates tumour response to PD-L1 blockade by contributing to exclusion of T cells. Nature.

[18] Zhao Z, Liu H, Zhou X, Fang D, Ou X, Ye J (2021). Necroptosis-related lncRNAs: predicting prognosis and the distinction between the cold and hot tumors in gastric cancer. J Oncol.

[19] Topalian SL, Hodi FS, Brahmer JR, Gettinger SN, Smith DC, McDermott DF (2012). Safety, activity, and immune correlates of anti-PD-1 antibody in cancer. N Engl J Med.

[20] Snyder AG, Hubbard NW, Messmer MN, Kofman SB, Hagan CE, Orozco SL (2019). Intratumoral activation of the necroptotic pathway components RIPK1 and RIPK3 potentiates antitumor immunity. Sci Immunol.

[21] Vodnala SK, Eil R, Kishton RJ, Sukumar M, Yamamoto TN, Ha N-H (2019). T cell stemness and dysfunction in tumors are triggered by a common mechanism. Science.

[22] Gao X-Q, Liu C-Y, Zhang Y-H, Wang Y-H, Zhou L-Y, Li X-M (2022). The circRNA CNEACR regulates necroptosis of cardiomyocytes through Foxa2 suppression. Cell Death Differ.

[23] Haga S, Kanno A, Ozawa T, Morita N, Asano M, Ozaki M (2018). Detection of necroptosis in ligand-mediated and hypoxia-induced injury of hepatocytes using a novel optic probe-detecting receptor-interacting protein (RIP)1/RIP3 binding. Oncol Res.

[24] He C, Liu Y, Huang Z, Yang Z, Zhou T, Liu S (2021). A specific RIP3 subpopulation of microglia promotes retinopathy through a hypoxia-triggered necroptotic mechanism. Proc Natl Acad Sci USA.

[25] Brumatti G, Ma C, Lalaoui N, Nguyen N-Y, Navarro M, Tanzer MC (2016). The caspase-8 inhibitor emricasan combines with the SMAC mimetic birinapant to induce necroptosis and treat acute myeloid leukemia. Sci Transl Med..

[26] Fulda S, Vucic D (2012). Targeting IAP proteins for therapeutic intervention in cancer. Nat Rev Drug Discov.

[27] Beug ST, Beauregard CE, Healy C, Sanda T, St-Jean M, Chabot J (2017). Smac mimetics synergize with immune checkpoint inhibitors to promote tumour immunity against glioblastoma. Nat Commun.

[28] Pietzak EJ, Bagrodia A, Cha EK, Drill EN, Iyer G, Isharwal S (2017). Next-generation sequencing of nonmuscle invasive bladder cancer reveals potential biomarkers and rational therapeutic targets. Eur Urol.

[29] Robertson AG, Kim J, Al-Ahmadie H, Bellmunt J, Guo G, Cherniack AD (2017). Comprehensive molecular characterization of muscle-invasive bladder cancer. Cell.

[30] Sfakianos JP, Gul Z, Shariat SF, Matin SF, Daneshmand S, Plimack E (2021). Genetic differences between bladder and upper urinary tract carcinoma: implications for therapy. Eur Urol Oncol.

[31] Knowles MA, Hurst CD (2015). Molecular biology of bladder cancer: new insights into pathogenesis and clinical diversity. Nat Rev Cancer.

[32] Tan TZ, Rouanne M, Tan KT, Huang RY-J, Thiery J-P (2019). Molecular subtypes of urothelial bladder cancer: results from a meta-cohort analysis of 2411 tumors. Eur Urol.

[33] Kacew A, Sweis RF (2020). Alterations in the era of immunotherapy for urothelial bladder cancer. Front Immunol.

[34] Yan J, Wan P, Choksi S, Liu Z-G (2022). Necroptosis and tumor progression. Trends Cancer.

[35] Zang X, Song J, Li Y, Han Y (2022). Targeting necroptosis as an alternative strategy in tumor treatment: from drugs to nanoparticles. J Control Release.

