# Supplementary figures and images for: MLKL and other necroptosis-related genes promote the tumor immune cell infiltration, guiding for the administration of immunotherapy in bladder urothelial carcinoma
Source: Apoptosis. 2023 Mar 31;28(5-6):892–911. doi: 10.1007/s10495-023-01830-8 (PMC10232593; doi:10.1007/s10495-023-01830-8)

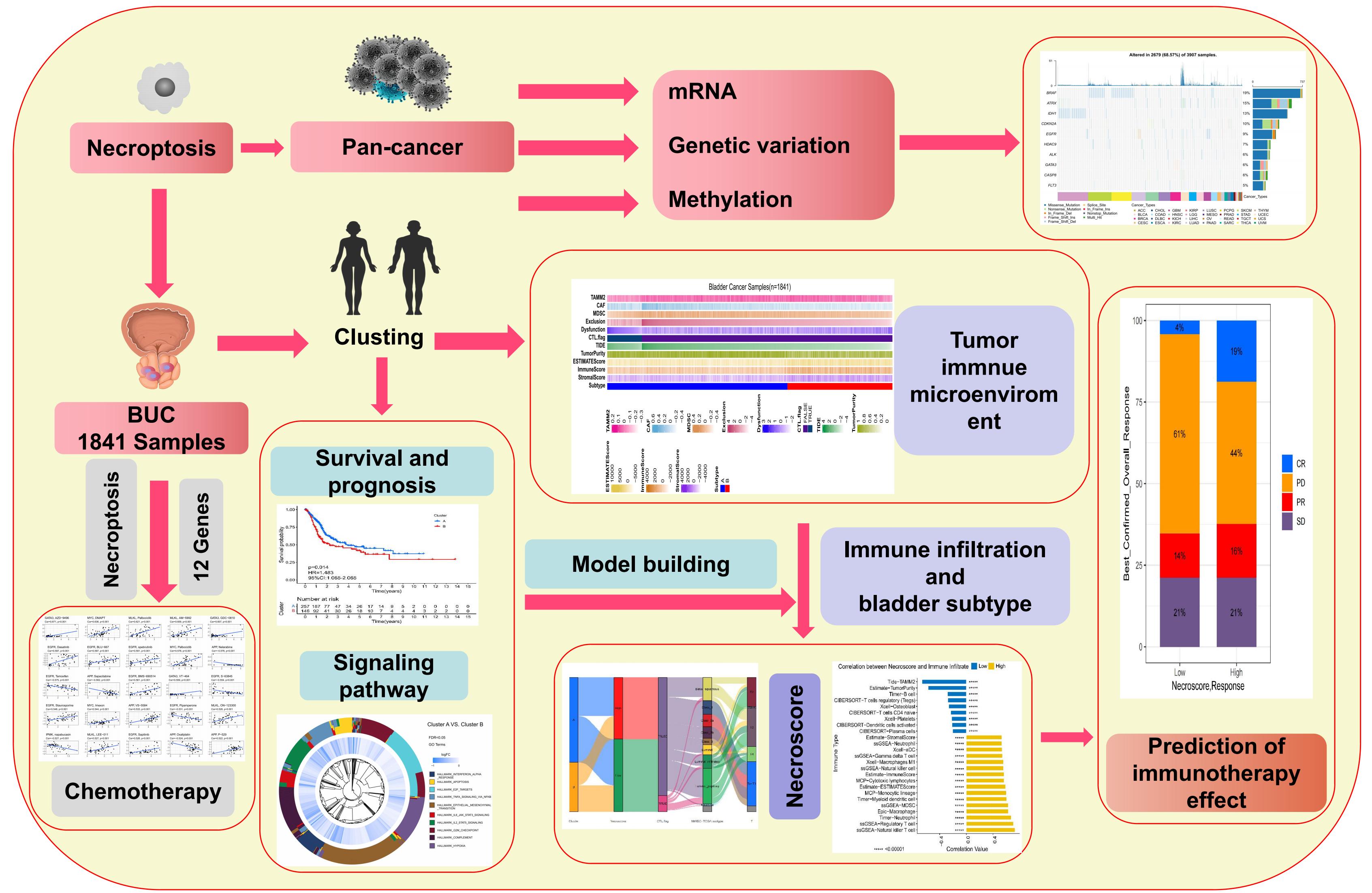

Supplement: Supplementary file 1 — Supplementary file1 (JPG 667 KB) [file 10495_2023_1830_MOESM1_ESM.jpg]

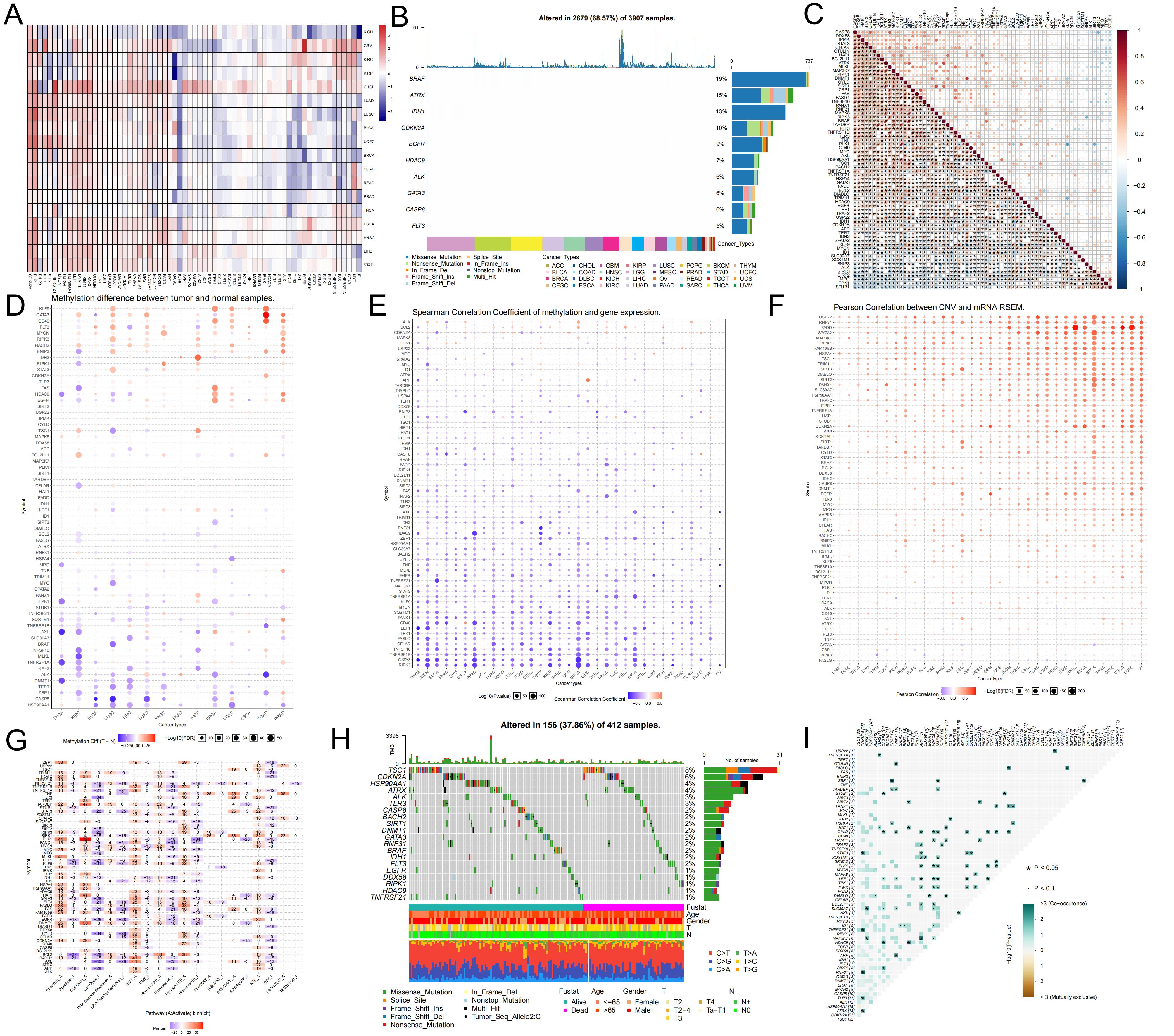

Supplement: Supplementary file 2 — Supplementary file2 (JPG 1996 KB) [file 10495_2023_1830_MOESM2_ESM.jpg]

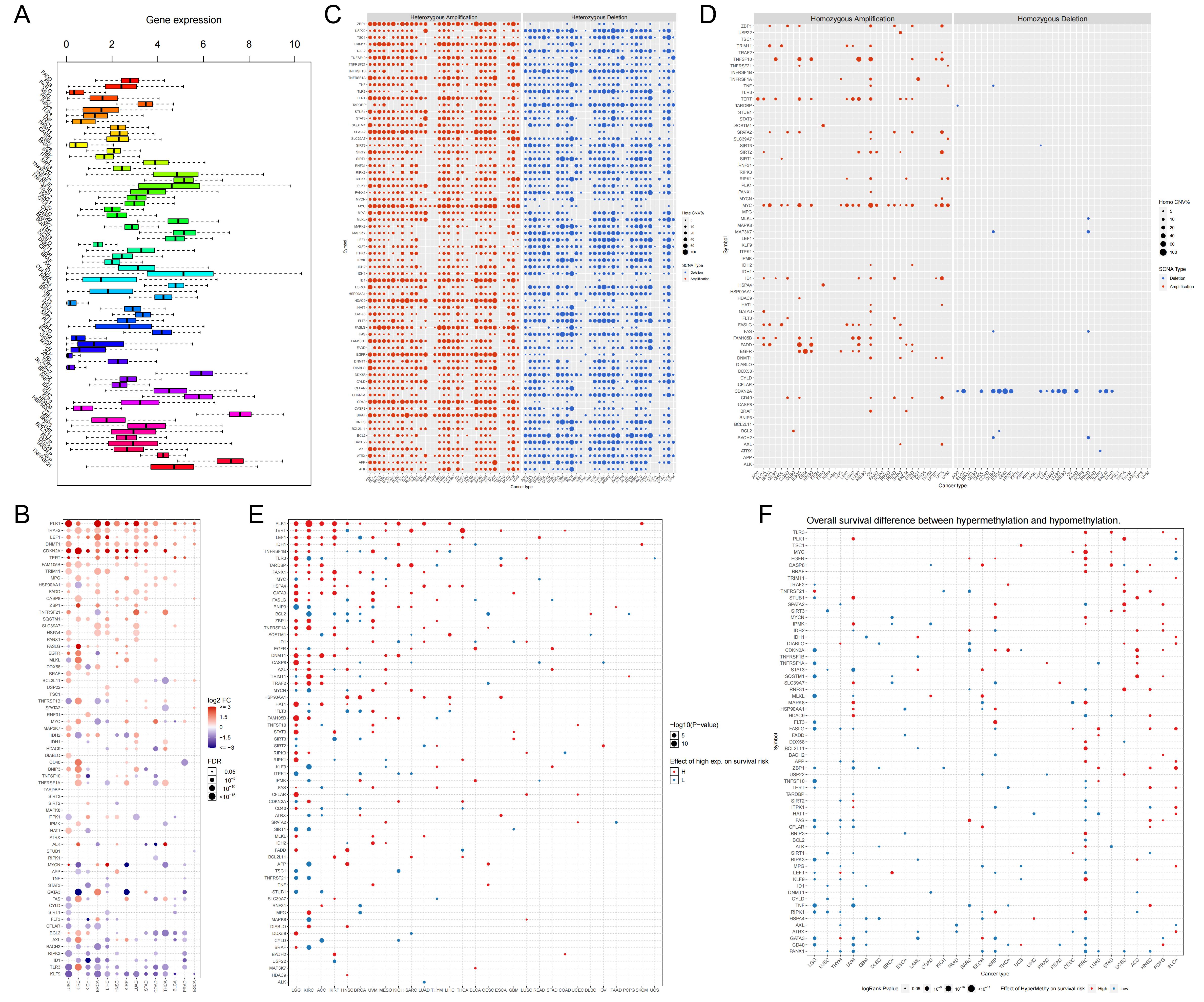

Supplement: Supplementary file 3 — Supplementary file3 (JPG 2057 KB) [file 10495_2023_1830_MOESM3_ESM.jpg]

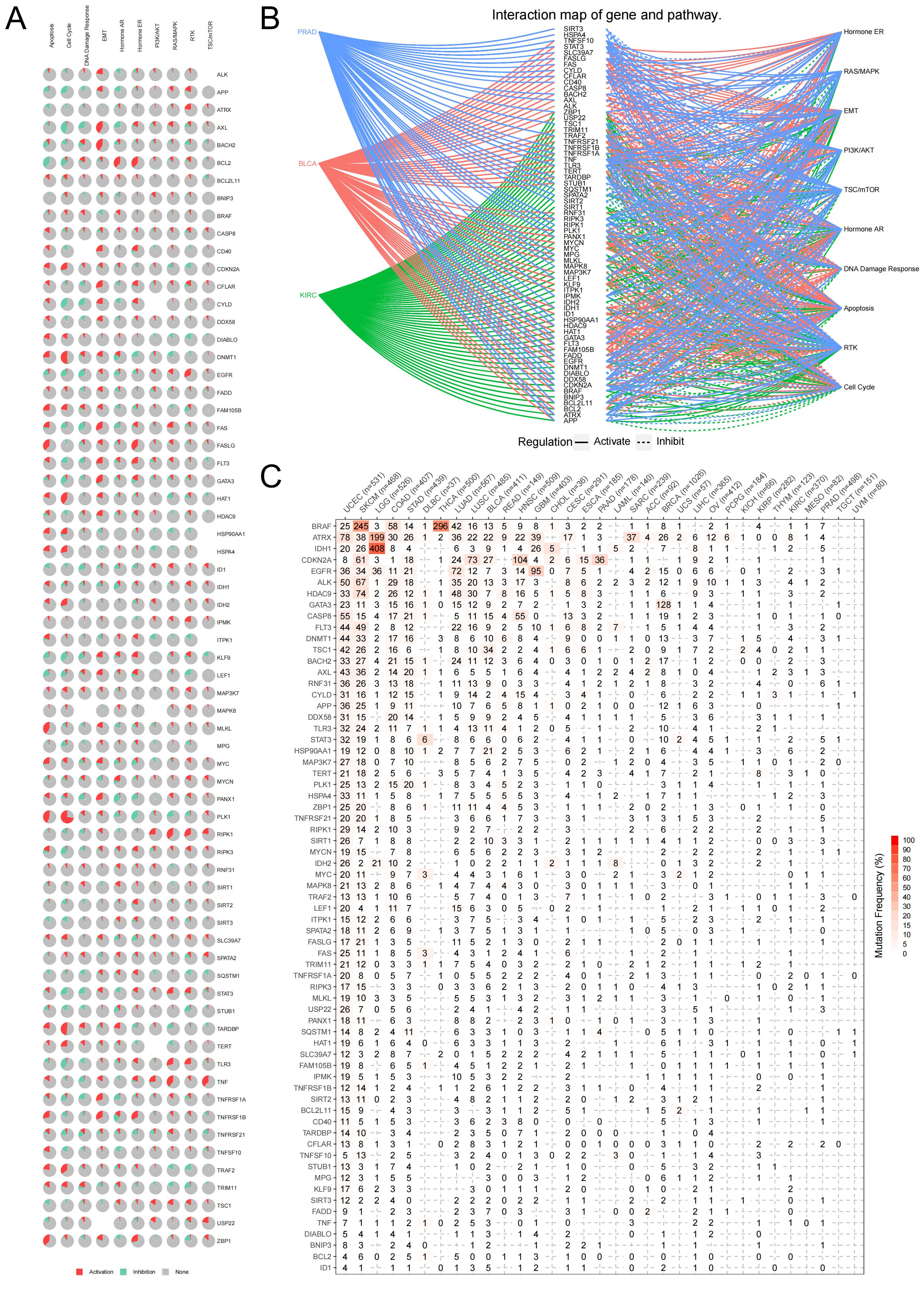

Supplement: Supplementary file 4 — Supplementary file4 (JPG 1675 KB) [file 10495_2023_1830_MOESM4_ESM.jpg]

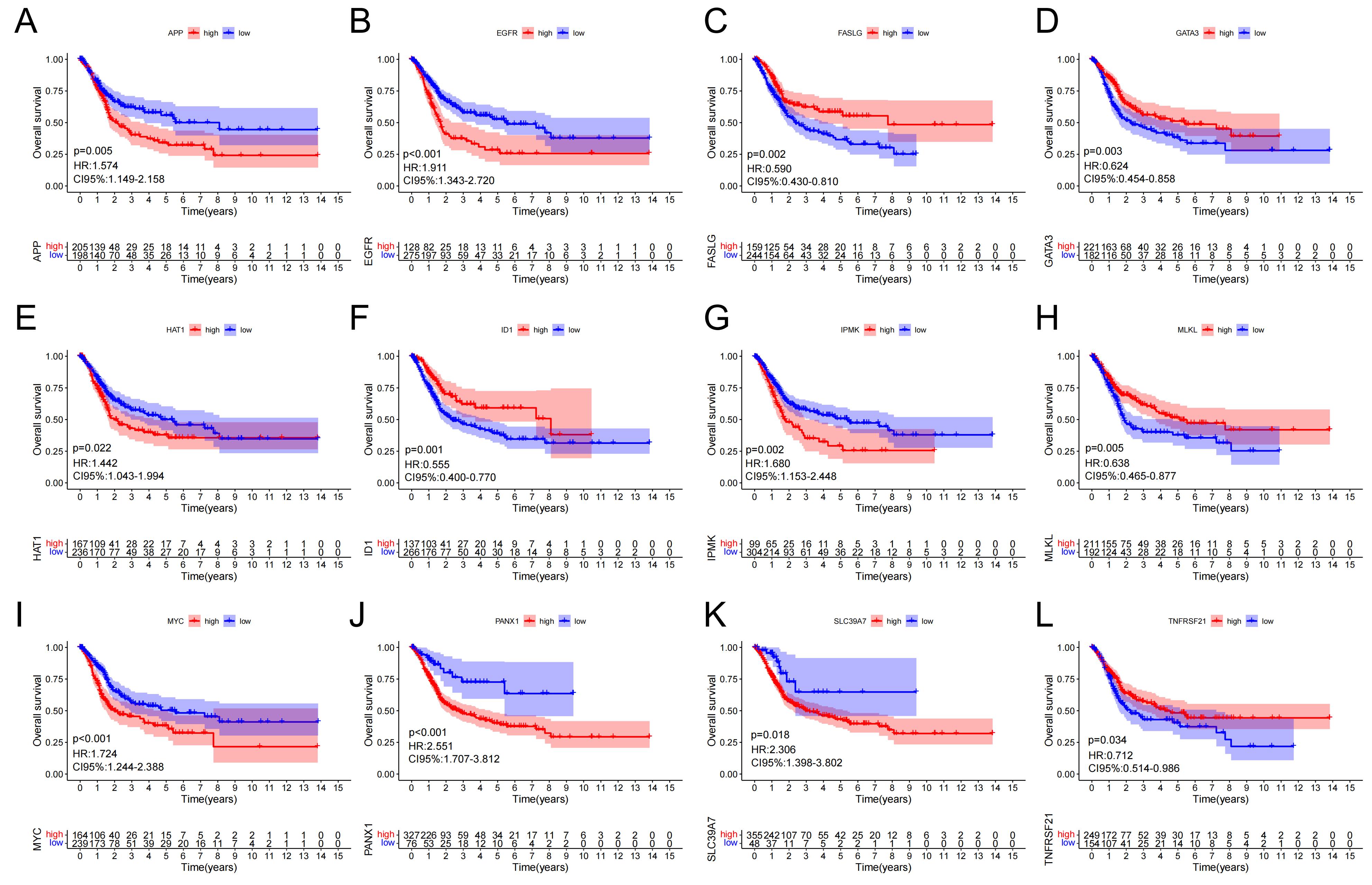

Supplement: Supplementary file 5 — Supplementary file5 (JPG 875 KB) [file 10495_2023_1830_MOESM5_ESM.jpg]

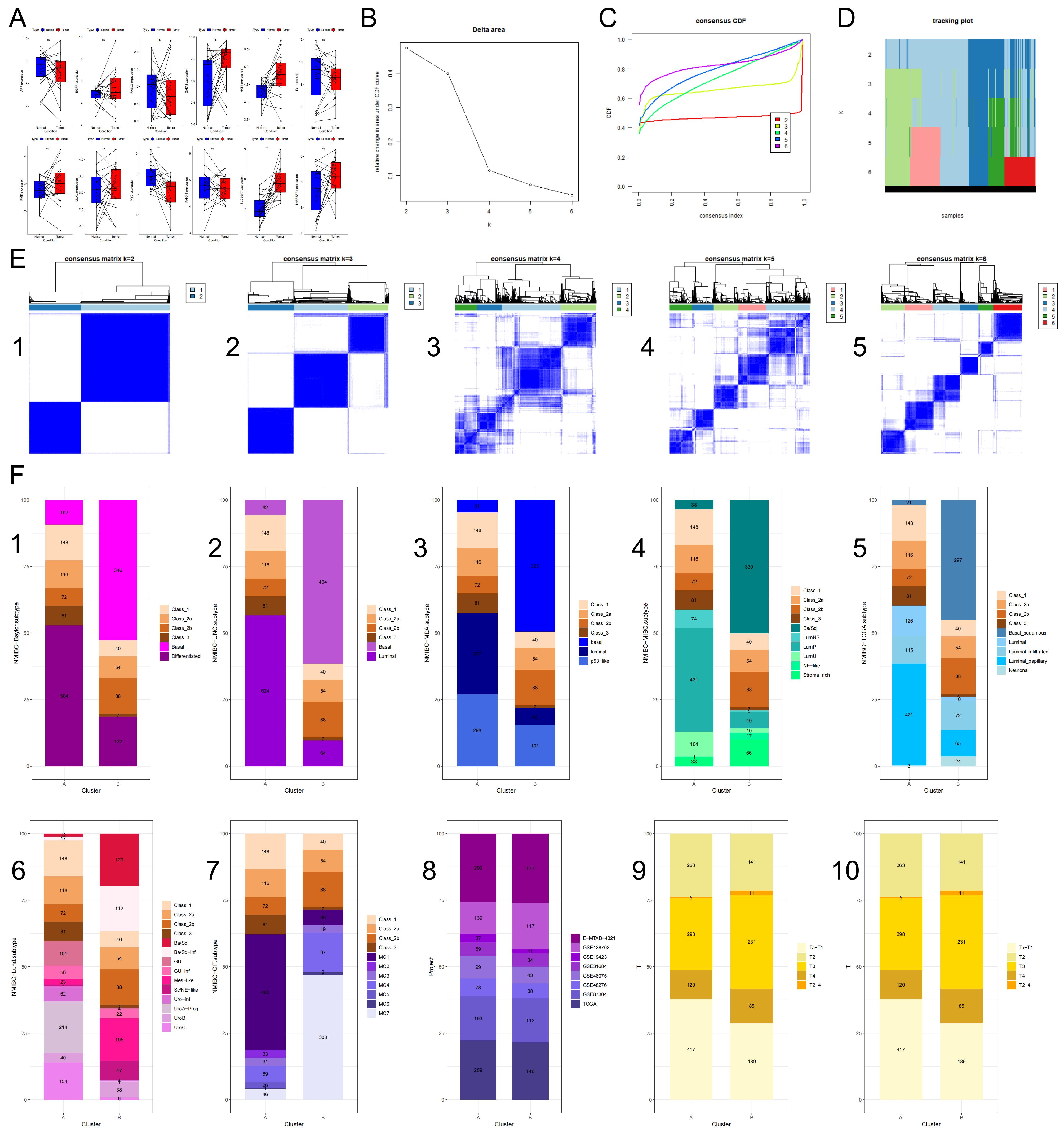

Supplement: Supplementary file 6 — Supplementary file6 (JPG 1027 KB) [file 10495_2023_1830_MOESM6_ESM.jpg]

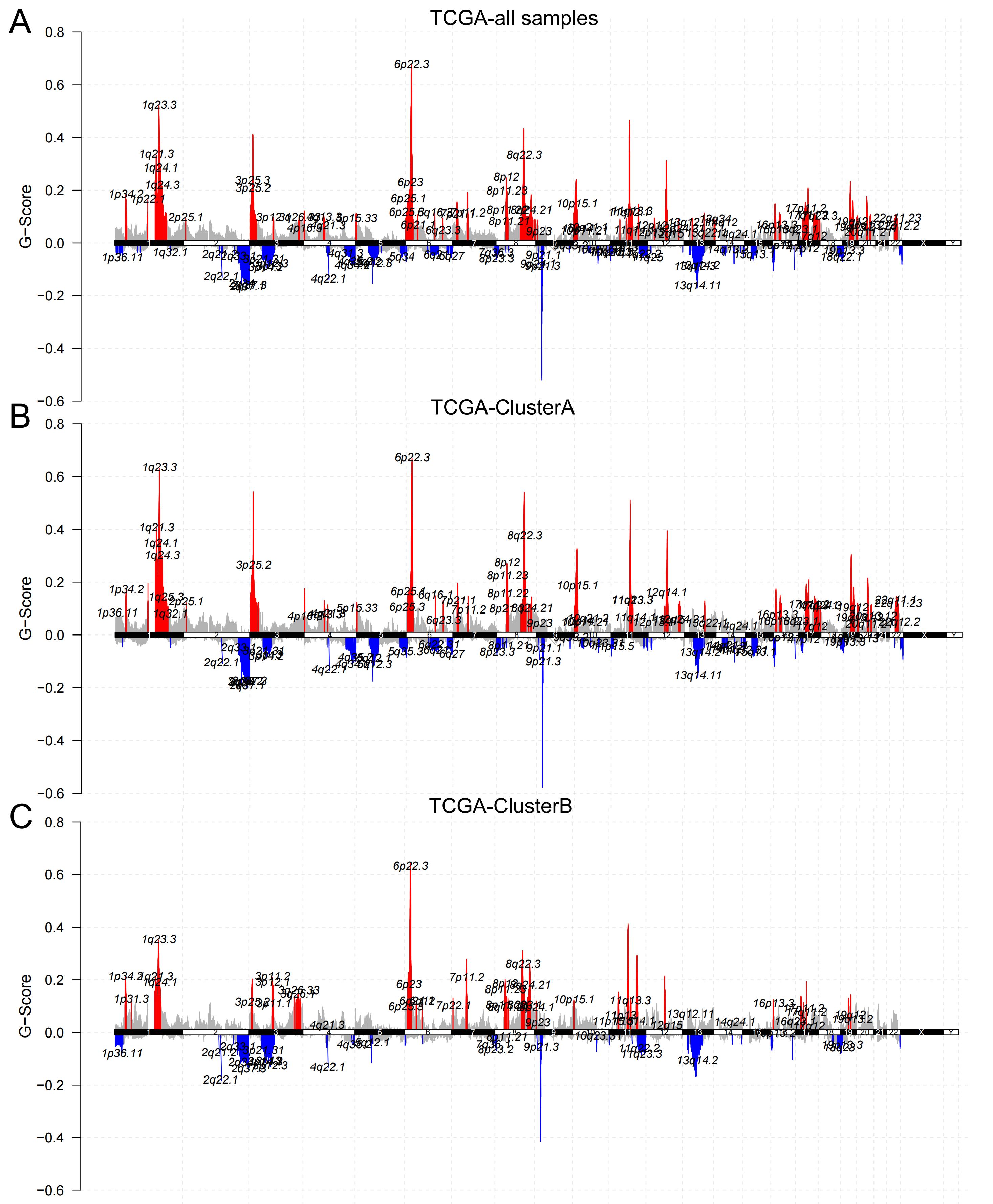

Supplement: Supplementary file 7 — Supplementary file7 (JPG 740 KB) [file 10495_2023_1830_MOESM7_ESM.jpg]

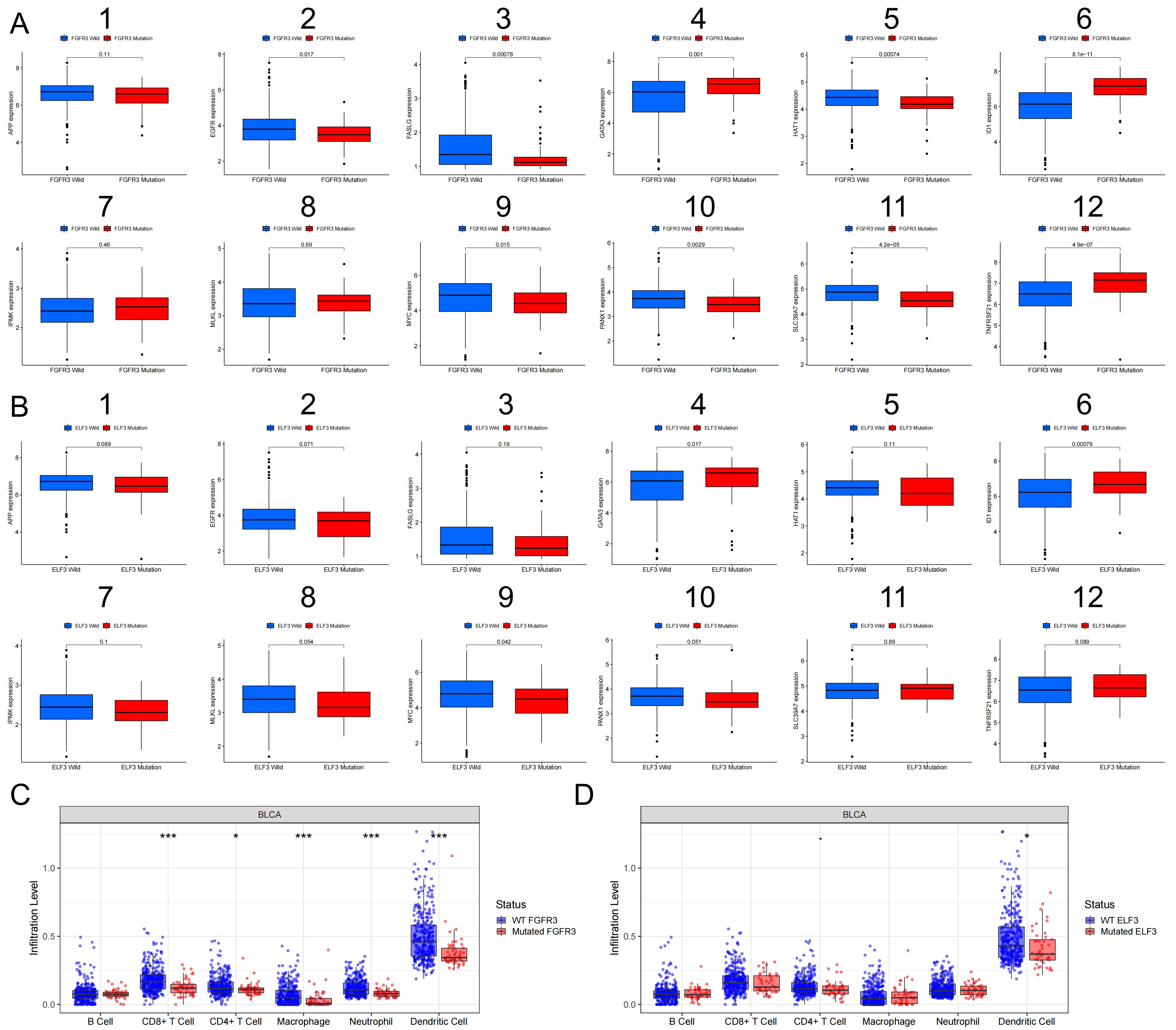

Supplement: Supplementary file 8 — Supplementary file8 (JPG 838 KB) [file 10495_2023_1830_MOESM8_ESM.jpg]

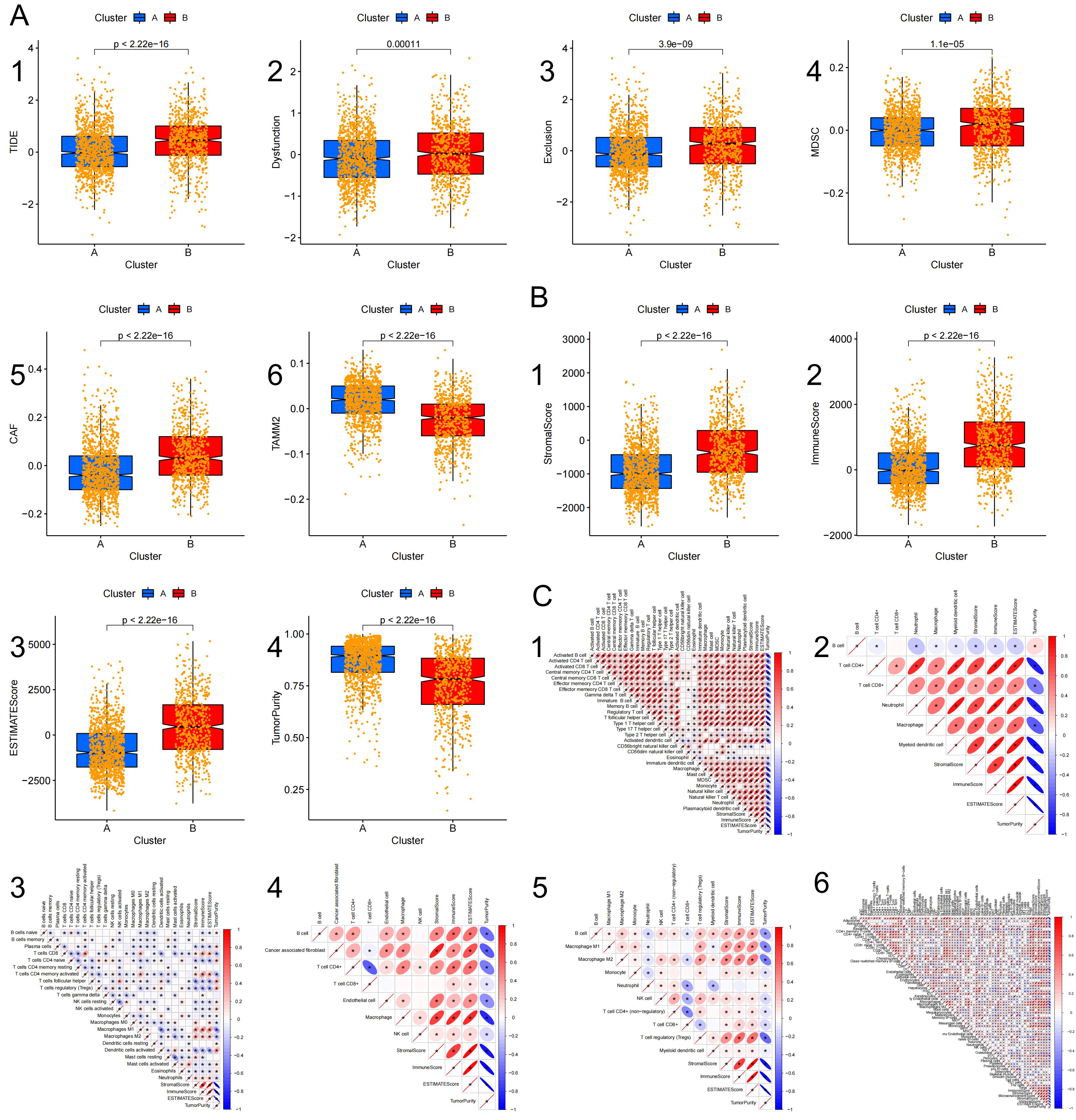

Supplement: Supplementary file 9 — Supplementary file9 (JPG 1451 KB) [file 10495_2023_1830_MOESM9_ESM.jpg]

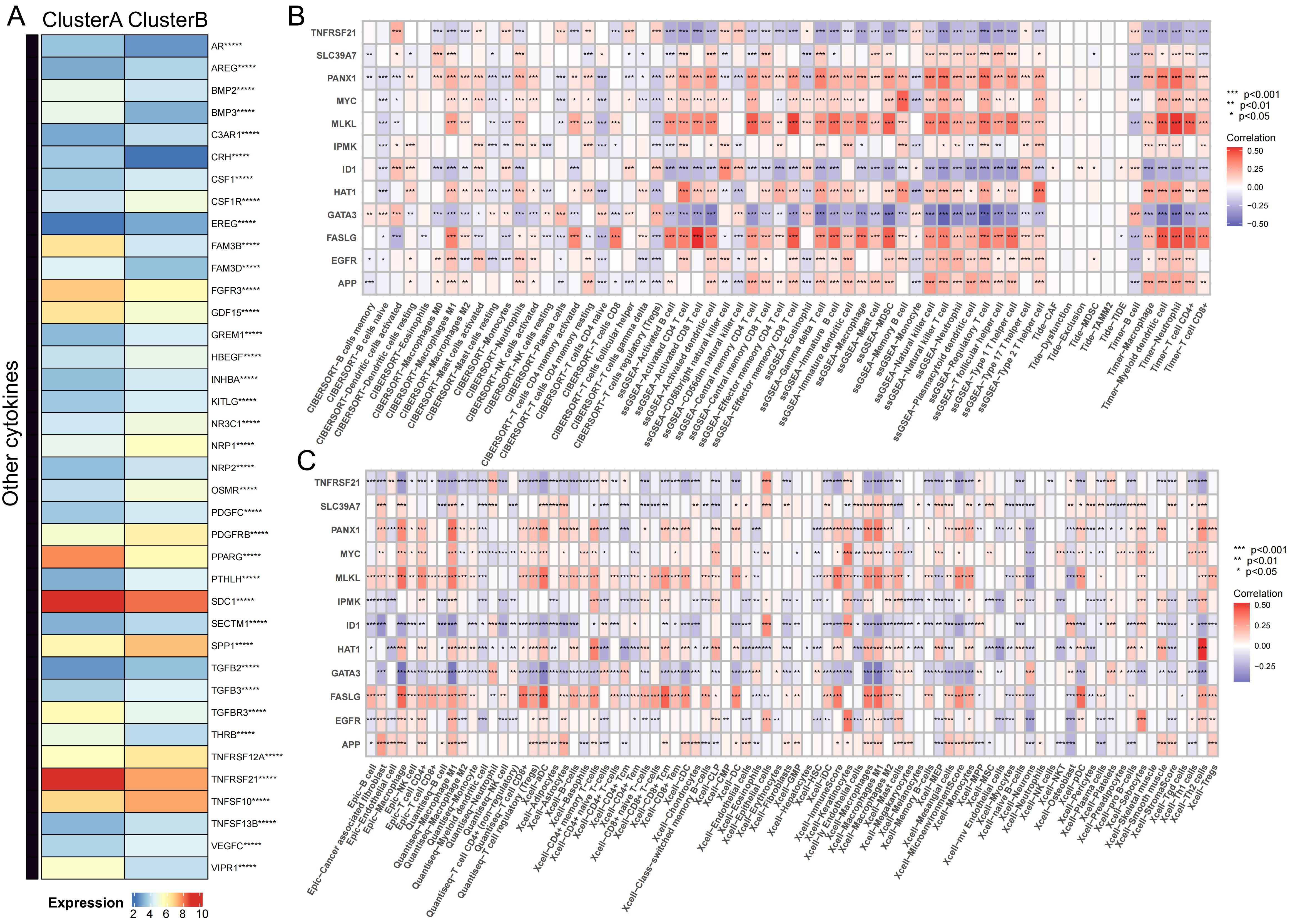

Supplement: Supplementary file 10 — Supplementary file10 (JPG 1731 KB) [file 10495_2023_1830_MOESM10_ESM.jpg]

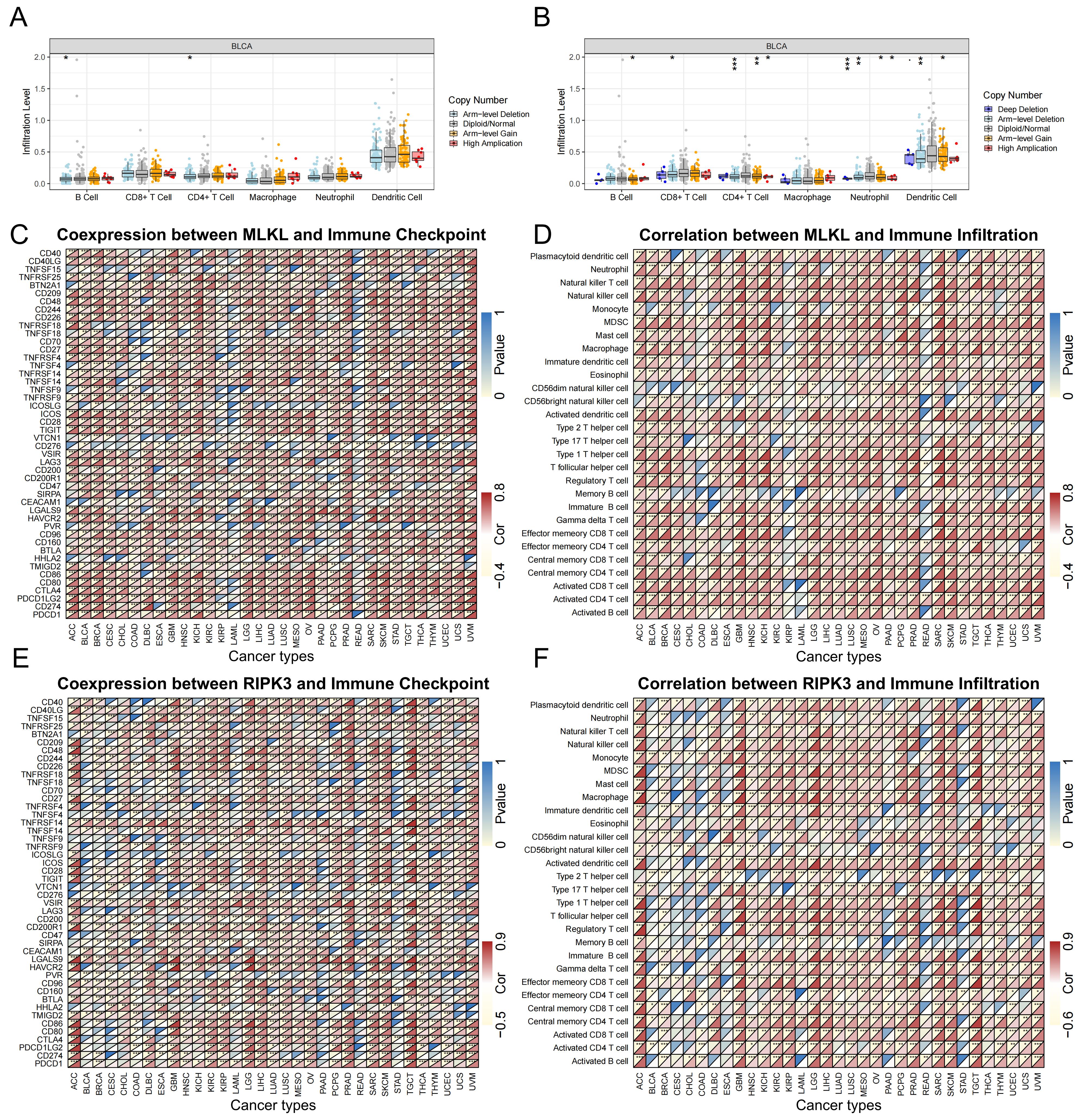

Supplement: Supplementary file 11 — Supplementary file11 (JPG 3637 KB) [file 10495_2023_1830_MOESM11_ESM.jpg]

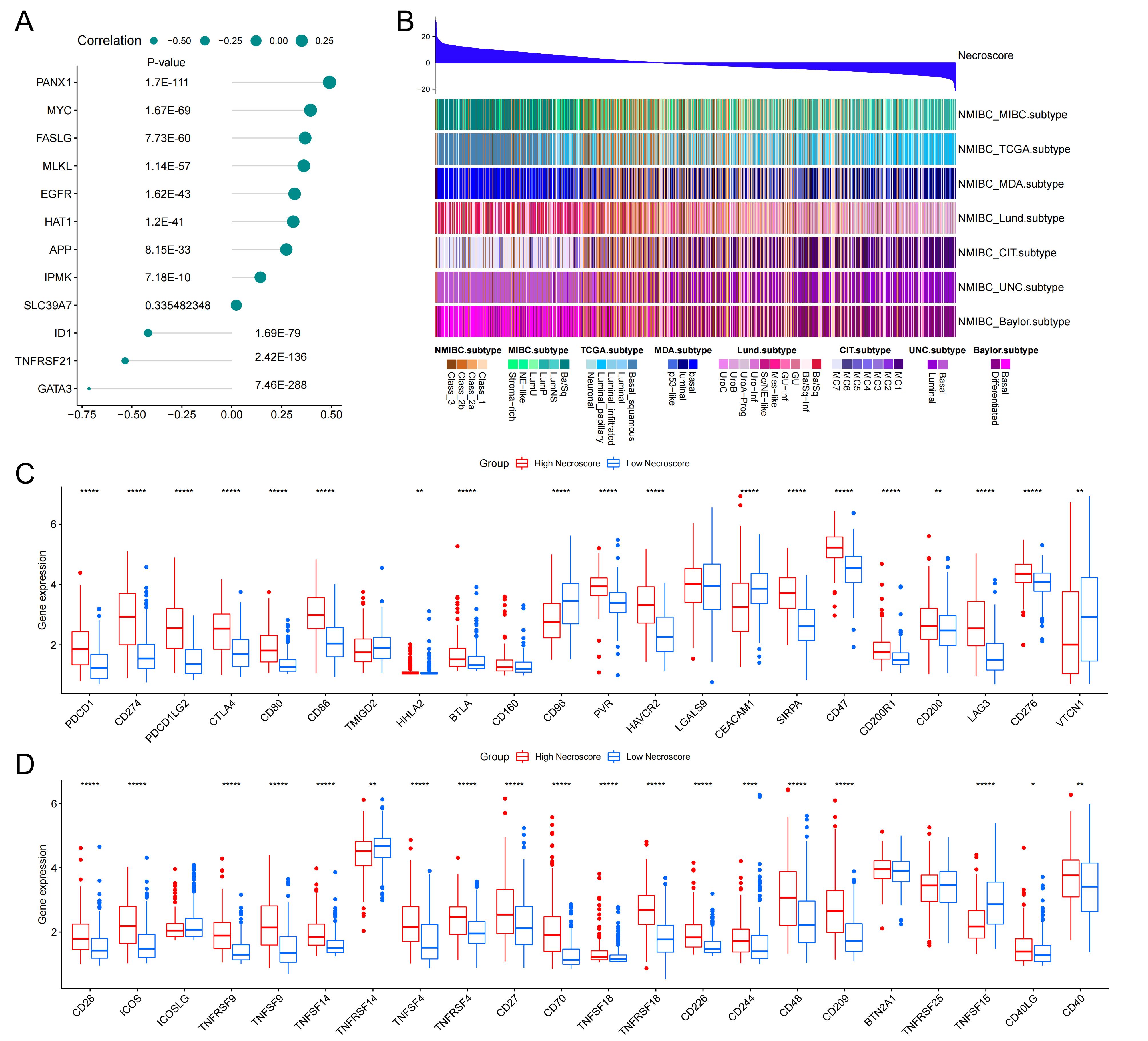

Supplement: Supplementary file 12 — Supplementary file12 (JPG 1193 KB) [file 10495_2023_1830_MOESM12_ESM.jpg]

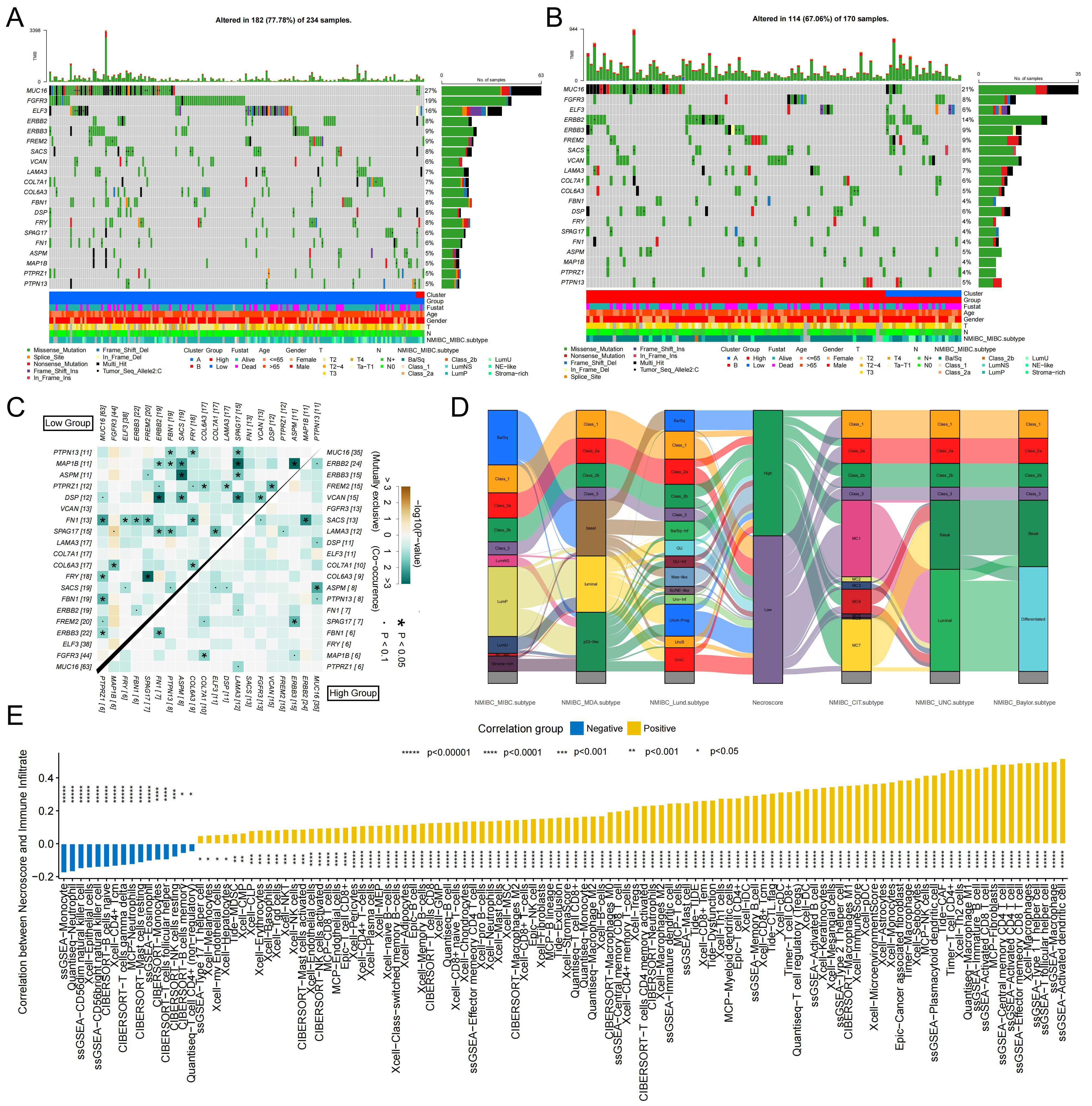

Supplement: Supplementary file 13 — Supplementary file13 (JPG 1980 KB) [file 10495_2023_1830_MOESM13_ESM.jpg]

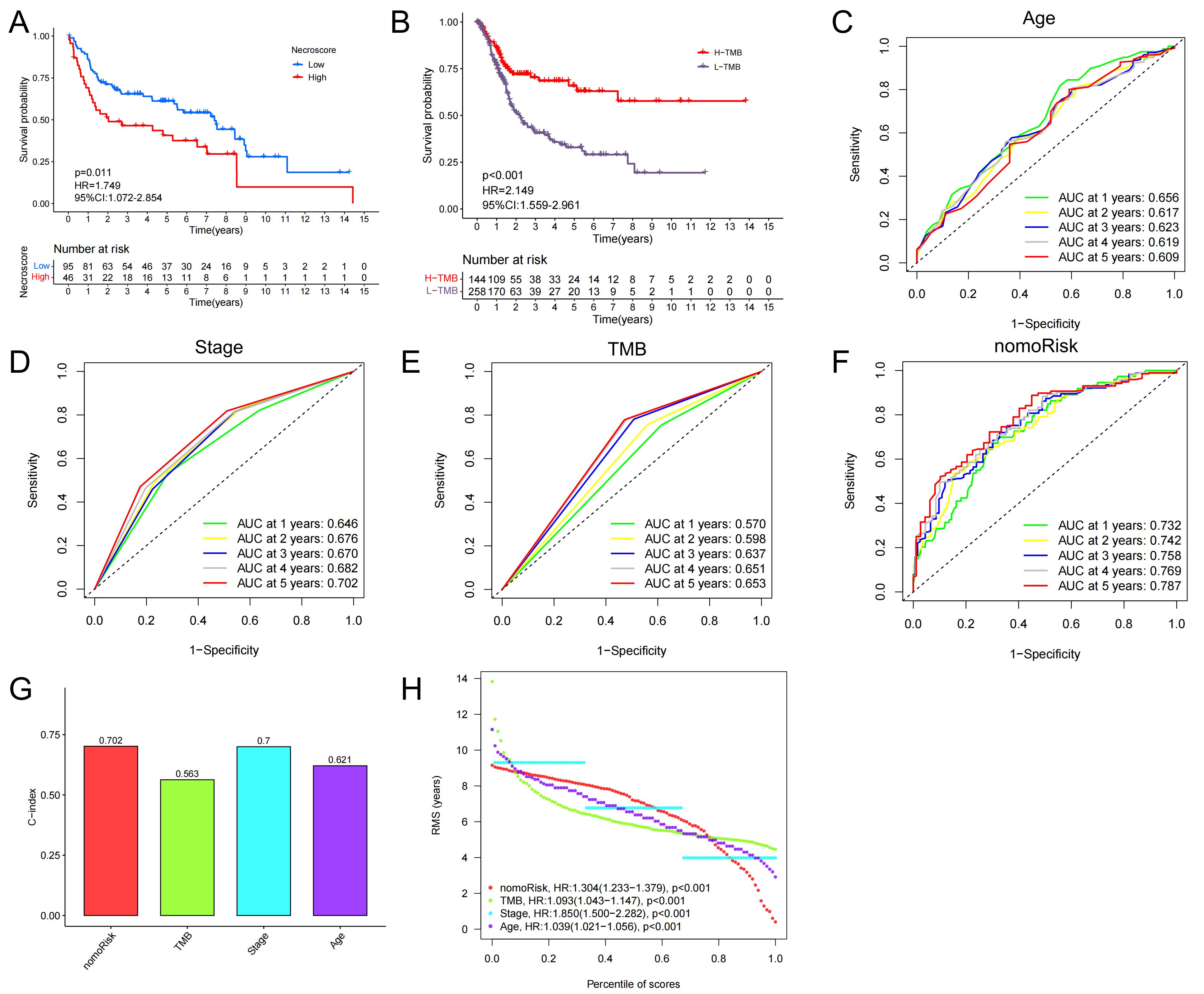

Supplement: Supplementary file 14 — Supplementary file14 (JPG 763 KB) [file 10495_2023_1830_MOESM14_ESM.jpg]

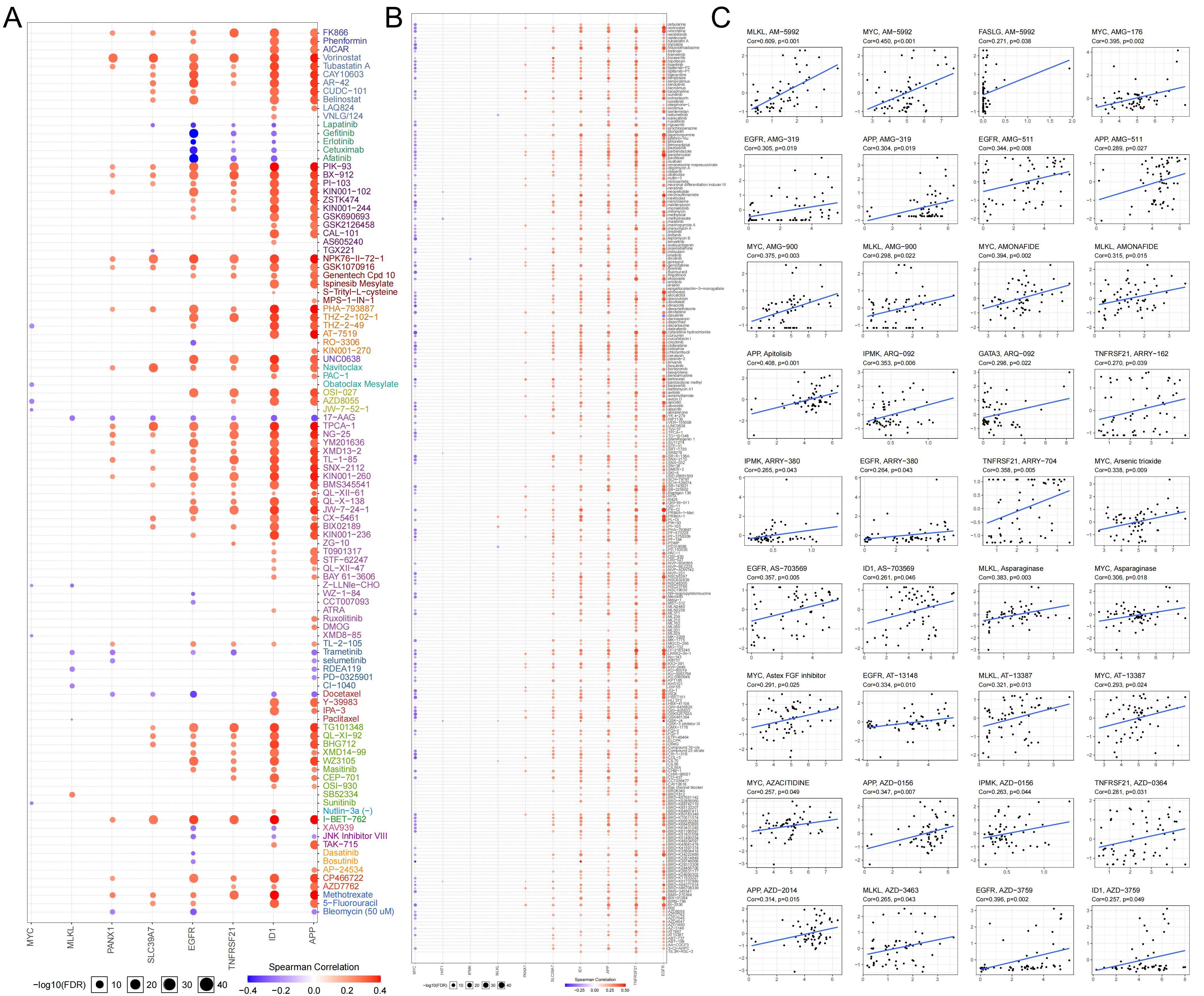

Supplement: Supplementary file 15 — Supplementary file15 (JPG 1757 KB) [file 10495_2023_1830_MOESM15_ESM.jpg]

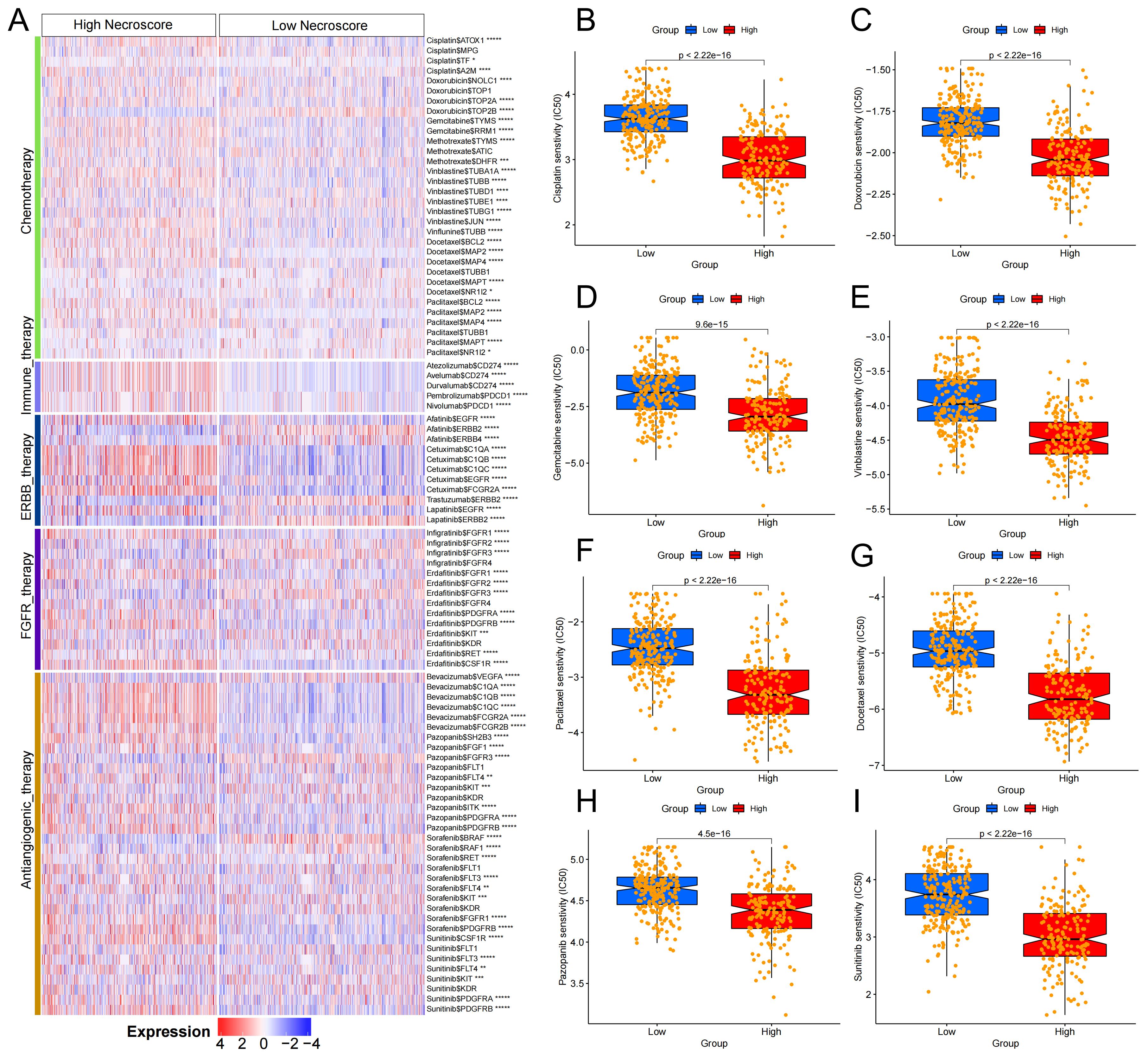

Supplement: Supplementary file 16 — Supplementary file16 (JPG 1749 KB) [file 10495_2023_1830_MOESM16_ESM.jpg]

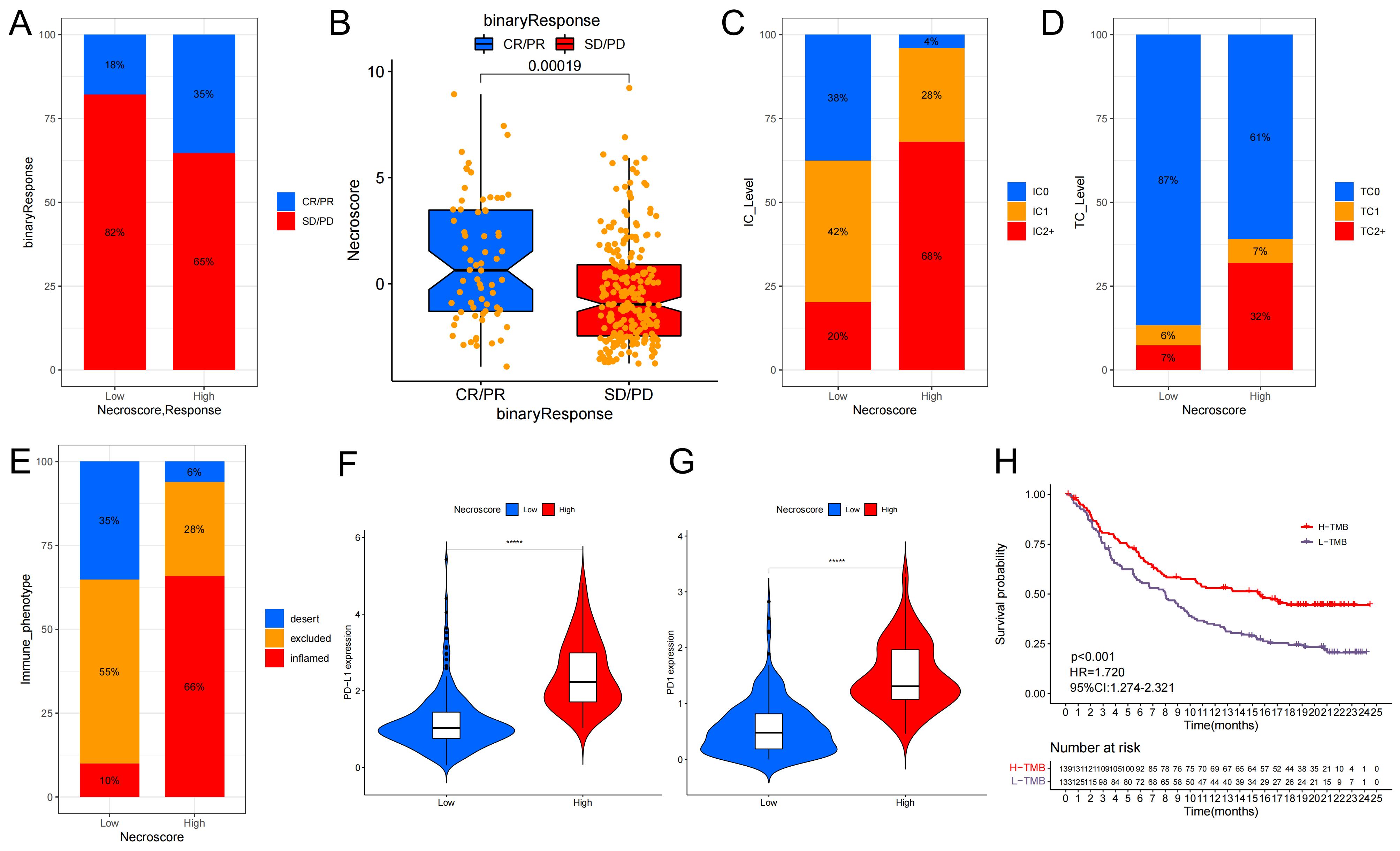

Supplement: Supplementary file 17 — Supplementary file17 (JPG 521 KB) [file 10495_2023_1830_MOESM17_ESM.jpg]
