# Supplementary material for: MLKL and other necroptosis-related genes promote the tumor immune cell infiltration, guiding for the administration of immunotherapy in bladder urothelial carcinoma
Source: Apoptosis. 2023 Mar 31;28(5-6):892–911. doi: 10.1007/s10495-023-01830-8 (PMC10232593; doi:10.1007/s10495-023-01830-8)
Supplement: Supplementary file 18 — Supplementary file18 (DOC 76 KB) [file 10495_2023_1830_MOESM18_ESM.doc]

**Materials and Methods**

**Data sources**

In the pan-cancer analysis, we collected RNA-seq (FPKM value), survival, phenotype, and single nucleotide variations data of 33 cancer types from the Cancer Genome Atlas (TCGA) through the UCSC Xena website (<http://xena.ucsc.edu/>). Part of the pan-cancer analysis was conducted using the GSCALite website[1] (<http://bioinfo.life.hust.edu.cn/web/GSCALite/>). We also obtained tumor stemness score and immune subtype data from the UCSC Xena website. For our study on bladder urothelial carcinoma (BUC), we collected six BUC cohorts (GSE19423, GSE31684, GSE48075, GSE48276, GSE87304, GSE128702) from the Gene Expression Omnibus database (GEO) and downloaded 476 urothelial carcinomas (E-MTAB-4321) RNA-seq and clinical data from the "ArrayExpress" database (<https://www.ebi.ac.uk/arrayexpress/>). In addition, we downloaded RNA-seq, clinical phenotype, SNV, copy number variation (CNV), pathological slide, and other data of BUC from TCGA (<https://portal.gdc.cancer.gov/>). We used a total of 1841 samples from all BUC cohorts to correct for BLCA cohort batch effects using the "battle" algorithm of the R package "SVA". For the IMvigor210 cohort (a research cohort of anti-PD-L1 immunotherapy in BUC), complete gene expression, phenotype, and CNV data are available at <http://research-pub.gene.com/IMvigor210CoreBiologies/packageVersions/>. All the aforementioned databases are publicly available, and all gene expression levels were calculated by Log2(X+1). The clinical tissue samples were obtained from bladder cancer patients who underwent total bladder cystectomy at the Second Affiliated Hospital of Tianjin Medical University (Tianjin, China), and professional pathologists checked the samples to obtain pathological grading. The study was approved by the Ethics Committee of Tianjin Medical University and strictly followed the Helsinki Declaration of Human Rights.

**Comprehensive analysis of 33 cancer types**

We analyzed the expression levels of 67 necroptosis genes in 33 types of tumors, and delineated their distribution as well as the correlation between each gene using the R packages "pheatmap" and "corrplot". Additionally, we systematically analyzed the methylation modification, SNVs, and regulation of key signaling pathways in these 67 necroptosis genes across 33 cancer types using the GSCALite website.

**Analysis of survival differences**

We utilized the "survival" package in R to perform univariate regression analysis on survival time and survival status in pan-cancer (P-value < 0.05). We screened out 12 necroptosis-related prognostic genes, namely FASLG, MLKL, IPMK, MYC, PANX1, ID1, HAT1, GATA3, SLC39A7, EGFR, APP, and TNFRSF21. To visualize the survival curves for different necroptosis modules, NecroScore, and TMB, we employed the "survminer" and "survival" packages in R.

**Assessment of immune subtypes and tumor stemness**

To begin with, we performed gene deduplication in the expression matrix of non-normal tissue samples using the "limma" package[2]. Next, we grouped 12 necroptosis-related prognostic genes based on the immunophenotyping of pan-cancer samples, including BLCA (Immune C1: Wound Healing; Immune C2: IFN-gamma Dominant; Immune C3: Inflammatory; Immune C4: Lymphocyte Depleted; Immune C5: Immunologically Quiet; Immune C6: TGF-beta Dominant). We visualized the relationship between gene expression and different immune subtypes using the "ggplot2" and "reshapes" packages. Subsequently, we assessed the correlation of RNAss and DNAss scores with these 12 necroptosis genes in pan-cancer samples using the "corrplot" package.

**Construction of necroptosis regulator phenotypes**

We began by performing unsupervised cluster analysis[3] using K-Means based on Euclidean distance to identify necroptosis in BUC based on the expression levels of 12 prognostic necroptosis-related molecules. The resulting phenotype was used to classify patients. To determine the optimal number of clusters in the BLCA cohort, we used the consistent clustering algorithm and verified its stability. Each step was carried out using the R package "ConsencesclusterPlus", and the procedure was repeated 100 times with a resampling rate of 80%[4].

**Molecular subtypes of BUC**

BUC phenotypes can be classified into NMIBC-subtype and MIBC-subtype based on whether they are muscle-invasive BUC or not. The MIBC-subtype includes CIT, Lund, MDA, TCGA, Baylor, UNC, and consensus subtypes[5-10]. We used the R packages "classifyNMIBC", "ConsensusMIBC" and "BLCAsubtyping"[11] to determine 1841 BUC subtypes and compared these phenotypes with two necroptotic subtypes.

**Differential gene expression analysis of two necroptotic phenotypes**

We used the R package "Limma" to identify differentially expressed genes (DEGs) for the two necroptosis patterns, with a screening criterion of FDR < 0.05. To run Gene Set Variation Analysis (GSVA), we used the "c2.cp.kegg.v7.5.1.symbols" gene set, and the R packages "clusterProfiler" and "enrichplot" were employed for Gene Ontology (GO) and hallmark gene sets analysis. For GSVA analysis of 1841 samples, we collected 12 sets of marking characteristics of BUC[11] and visualized them using the "ggridges" package.

**Identification of genetic alterations and copy number variation differences**

We utilized the R package "maftools" to generate waterfall plots of SNVs and heatmaps of gene co-mutation in the TCGA-BLCA cohort. For copy number analysis, we used GISTIC.2 to determine amplified genome and deleted gene sequences[12]. The analysis was conducted through the GenePattern (<https://cloud.genepattern.org/>).

**Estimation of immune infiltration for the necroptotic phenotype**

We utilized various methods to analyze immune cells and their infiltration in BUC. Firstly, we employed the Quantiseq, Timer, Mcp_counter, Xcell, cibersort, epic methods (via the R package "immunedeconv") and ssGSEA (a metagenes set of immune cells derived from Jia et al 2014)[13] to calculate the prevalence of immune cells. We predicted tumor purity and the presence of stromal/immune cells in tumor tissue using the "estimate" package. Furthermore, we utilized the Tumor Immunity Estimation Resource Database (TIMER, [https://cistrome.shinyapps.io/timer/)[14]](https://cistrome.shinyapps.io/timer/)%5B14%5D) to evaluate the effect of copy number model composition gene pairs and gene mutation types on immune cell infiltration[15]. We also calculated the Tumor Immune Dysfunction and Rejection (TIDE) scores for 1841 BUC patients using the website provided by Jiang et al[16] (<http://tide.dfci.harvard.edu/>). TIDE scores accurately predict immunotherapy response in cancer patients and assess two distinct tumor immune mechanisms: dysfunction of tumor-infiltrating cytotoxic T lymphocytes (CTLs) and rejection of CTLs by immunosuppressive factors. We performed PCA scoring of 1841 samples using genes from core biological processes[17]. Furthermore, we analyzed immune cell infiltration in pan-cancer using the ssGSEA method and visualized the relationship between the gene expression levels of RIPK3 and MLKL and immune cell infiltration and immune checkpoints using the R packages "RColorBrewer" and "reshape2".

**PCA scoring calculation**

To conduct gene expression scoring analysis, we first z-score transformed the expression of each gene in the signature. Then, we performed principal component analysis to extract principal component 1 as the gene feature score. This approach offers the advantage of focusing the ensemble's score on the largest block of highly correlated (or anti-correlated) genes in the ensemble, while down-weighting the contributions of genes that are not tracked with other ensemble members[17].

**Construction of a prognostic model**

We first evaluated the impact of Age, Gender, Grade, TMB, and NecroScore on BUC prognosis using multivariate Cox analysis with the "survival" package, and then visualized the results using the "forestplot" package. Subsequently, we used these four prognostic factors to construct a prognostic scoring model with the "regplot" package. Finally, we assessed the reliability of the model using ROC curve and C-index analyses.

**Drug Sensitivity Analysis**

We obtained some of the data on necroptosis-related molecules and chemotherapeutic drug sensitivity from the GSCALite website. We assessed necroptosis molecules and drug sensitivity in 60 cell lines using the NCI-60 database from the CellMiner website (<https://discover.nci.nih.gov/cellminer/home.do>). Data on drug targets were obtained from the DrugBank database (<https://go.drugbank.com/>)[18]. We used public pharmacogenomics databases, namely the Genomics of Cancer Drug Sensitivity (GDSC, <https://www.cancerrxgene.org/>) and the Cancer Therapy Response Portal (CTRP, <https://portals.broadinstitute.org/>), to predict chemotherapy drug sensitivity. We predicted chemotherapeutic drug sensitivity using the R package "pRRophetic"[19], and then performed a correlation analysis between drug sensitivity data and NecroScore with a filter condition of P<0.001.

**Prediction of response to immune checkpoint inhibitors**

We evaluated responders (comprising patients with complete and partial responses) and non-responders (comprising patients with stable and progressive disease) in the IMvigor210 cohort using the NecroScore algorithm. To investigate the role of NecroScore in anti-PD1 and anti-CTLA4 immunity, we utilized the Submap algorithm, which is a tool for comparing expression profiles[20], and human immunotherapy transcriptome data came from Whijae Roh et al[21].

**RNA extraction and Analysis**

As per the manufacturer's instructions, RNA was extracted using the Trizol reagent. Next, 1 μg of RNA underwent reverse transcription using Superscript III transcriptase (Invitrogen) to obtain cDNA. The obtained cDNA was then used for qPCR analysis on a Quantagene q225 system. The primers used and RNA expression levels can be found in Supplementary Table 14.

**Western Blot Analysis**

Total protein was extracted from tumor tissues using RIPA (Biosharp) and PMSF, and the protein concentration was determined using the BCA kit. Equal amounts of protein (40 μg) were separated on 10% acrylamide gels by SDS-polyacrylamide gel electrophoresis (PAGE), and transferred to polyvinylidene difluoride (PVDF) membranes (Millipore, Billerica, MA). The membranes were blocked in 5% fat-free milk and incubated overnight at 4℃ with primary antibodies including rabbit anti-GAPDH (1: 1000, affinity), rabbit anti-CD8 (1: 1000, affinity), rabbit anti-MLKL (1: 1000, affinity), rabbit anti-c-MYC (1: 1000, affinity), rabbit anti-GATA3 (1: 1000, affinity), rabbit anti-TNFRSF21 (DR6) (1: 1000, affinity), rabbit anti-EGFR (1: 1000, affinity); rabbit anti-p-RIPK1 (Ser166, 1: 1000, affinity); rabbit anti-p-RIPK3 (Ser227, 1:1000, Abcam); rabbit anti-p-MLKL (MLKL (Ser358, 1: 1000, affinity). After washing, the PVDF membranes were incubated with anti-rabbit IgG at room temperature for 1 hour. The immunoreactive bands were detected using a chemiluminescent method and visualized using a Luminescent Imaging Workstation.

**IHC (immunohistochemistry)**

Mouse tumor specimens were obtained from the animal laboratory after surgery and fixed in formalin. Pathological sections were prepared by freezing, paraffin fixation, and sectioning. The sections were then incubated in an oven at 65°C for 45 minutes, dewaxed in xylene, and rehydrated with graded alcohol. Antigenic epitopes were repaired with citrate buffer (5 minutes at high heat, 10 minutes at medium heat), and the sections were treated with endogenous peroxidase for 20 minutes. Primary antibodies were added to the sections, which were refrigerated overnight at 4℃. The next day, the sections were warmed to room temperature and incubated with a secondary antibody for 1 hour. PBS buffer was used to wash the sections three times after each step between rehydration and color development. The sections were detected using the DAB color development agent and restained with hematoxylin after rinsing with tap water. After dehydration and transparency, the plates were sealed with neutral glue and photographed under a microscope (100×, 200×). The corresponding antibodies used were anti-Ki-67 (AF0198, 1:100, Affinity); anti-RIPK3 (DF10141, 1:100, Affinity); anti-MLKL (DF7412, 1:100, Affinity).

**IF (Immunofluorescence)**

Pathological sections of bladder cancer were first treated and subjected to immunofluorescence analysis following the protocol. The sections were dewaxed, treated with sodium citrate to repair antigenic epitopes, blocked with bovine serum albumin, and incubated overnight with the designated primary antibody. On the second day, the sections were incubated with the secondary antibody and DAPI and observed and photographed. The corresponding antibodies and reagents used were rabbit anti-MLKL (DF7412, 1:200, Affinity), Donkey anti-Rabbit secondary antibody, Alexa Fluor™ 647 (A-31573, 1:100, Invitrogen), DAPI ([F6057](https://www.sigmaaldrich.cn/CN/zh/product/sigma/f6057), Sigma).

**Cell derived xenograft**

T24 cells were transfected with control plasmid (Beyotime), shRIPK3 (Beyotime), shMLKL (Beyotime), and MLKL-OE (Axybio). Six-week-old male BALB/cnu mice (HFK Bio-Technology Co. Ltd., Beijing) were injected subcutaneously with 2×10^6^ T24 cells suspended in 200 μl Matrigel and 1640 medium in the control group, shRIPK3 group, shMLKL group, and MLKL-OE group, respectively, in the abdominal area. Tumor volume data were collected for at least 2 weeks and measured at the same time every day. Finally, the mice were euthanized, and the tumor volume was accurately measured.

All experiments involving mice were approved by the Animal Use and Care Committee of Tianjin Medical University and conformed to all regulatory standards.

**Statistical Analysis**

All statistical analyses were performed using R (<https://www.r-project.org/>), and a P-value < 0.05 (two-tailed) was considered statistically significant [Table S16]. The Pearson and Spearman correlation coefficients were used to determine the correlation between variables. The unpaired student t-test was used to estimate the statistical significance of normally distributed variables, while the Mann-Whitney U test (also known as the Wilcoxon rank-sum test) was used to analyze non-normally distributed variables. Kruskal-Wallis and one-way ANOVA tests were used to compare two or more groups. Contingency tables were analyzed by a two-sided Fisher's exact test. The "Surv cutpoint" function in the R package "Survminer" was used to evaluate the critical value for each dataset, and the Kaplan-Meier method was used to generate survival curves for each subgroup. The log-rank (Mantel-Cox) test was used to determine statistically significant differences. Finally, hazard ratios were calculated using a univariate Cox proportional hazards regression model.

1. Liu C-J, Hu F-F, Xia M-X, Han L, Zhang Q, Guo A-Y. GSCALite: a web server for gene set cancer analysis. Bioinformatics. 2018; 34: 3771-2.

2. Ritchie ME, Phipson B, Wu D, Hu Y, Law CW, Shi W, et al. limma powers differential expression analyses for RNA-sequencing and microarray studies. Nucleic Acids Res. 2015; 43: e47.

3. Qin X, Li J, Hu W, Yang J. Machine Learning K-Means Clustering Algorithm for Interpolative Separable Density Fitting to Accelerate Hybrid Functional Calculations with Numerical Atomic Orbitals. J Phys Chem A. 2020; 124: 10066-74.

4. Wilkerson MD, Hayes DN. ConsensusClusterPlus: a class discovery tool with confidence assessments and item tracking. Bioinformatics. 2010; 26: 1572-3.

5. Choi W, Porten S, Kim S, Willis D, Plimack ER, Hoffman-Censits J, et al. Identification of distinct basal and luminal subtypes of muscle-invasive bladder cancer with different sensitivities to frontline chemotherapy. Cancer Cell. 2014; 25: 152-65.

6. Damrauer JS, Hoadley KA, Chism DD, Fan C, Tiganelli CJ, Wobker SE, et al. Intrinsic subtypes of high-grade bladder cancer reflect the hallmarks of breast cancer biology. Proc Natl Acad Sci U S A. 2014; 111: 3110-5.

7. Mo Q, Nikolos F, Chen F, Tramel Z, Lee Y-C, Hayashi K, et al. Prognostic Power of a Tumor Differentiation Gene Signature for Bladder Urothelial Carcinomas. J Natl Cancer Inst. 2018; 110: 448-59.

8. Rebouissou S, Bernard-Pierrot I, de Reyniès A, Lepage M-L, Krucker C, Chapeaublanc E, et al. EGFR as a potential therapeutic target for a subset of muscle-invasive bladder cancers presenting a basal-like phenotype. Sci Transl Med. 2014; 6: 244ra91.

9. Robertson AG, Kim J, Al-Ahmadie H, Bellmunt J, Guo G, Cherniack AD, et al. Comprehensive Molecular Characterization of Muscle-Invasive Bladder Cancer. Cell. 2017; 171.

10. Sjödahl G, Lauss M, Lövgren K, Chebil G, Gudjonsson S, Veerla S, et al. A molecular taxonomy for urothelial carcinoma. Clin Cancer Res. 2012; 18: 3377-86.

11. Kamoun A, de Reyniès A, Allory Y, Sjödahl G, Robertson AG, Seiler R, et al. A Consensus Molecular Classification of Muscle-invasive Bladder Cancer. Eur Urol. 2020; 77: 420-33.

12. Mermel CH, Schumacher SE, Hill B, Meyerson ML, Beroukhim R, Getz G. GISTIC2.0 facilitates sensitive and confident localization of the targets of focal somatic copy-number alteration in human cancers. Genome Biol. 2011; 12: R41.

13. Jia Q, Wu W, Wang Y, Alexander PB, Sun C, Gong Z, et al. Local mutational diversity drives intratumoral immune heterogeneity in non-small cell lung cancer. Nat Commun. 2018; 9: 5361.

14. Acharya N, Madi A, Zhang H, Klapholz M, Escobar G, Dulberg S, et al. Endogenous Glucocorticoid Signaling Regulates CD8 T Cell Differentiation and Development of Dysfunction in the Tumor Microenvironment. Immunity. 2020; 53.

15. Li T, Fu J, Zeng Z, Cohen D, Li J, Chen Q, et al. TIMER2.0 for analysis of tumor-infiltrating immune cells. Nucleic Acids Res. 2020; 48: W509-W14.

16. Jiang P, Gu S, Pan D, Fu J, Sahu A, Hu X, et al. Signatures of T cell dysfunction and exclusion predict cancer immunotherapy response. Nat Med. 2018; 24: 1550-8.

17. Mariathasan S, Turley SJ, Nickles D, Castiglioni A, Yuen K, Wang Y, et al. TGFβ attenuates tumour response to PD-L1 blockade by contributing to exclusion of T cells. Nature. 2018; 554: 544-8.

18. Nadal R, Bellmunt J. Management of metastatic bladder cancer. Cancer Treat Rev. 2019; 76: 10-21.

19. Geeleher P, Cox N, Huang RS. pRRophetic: an R package for prediction of clinical chemotherapeutic response from tumor gene expression levels. PLoS One. 2014; 9: e107468.

20. Hoshida Y, Brunet J-P, Tamayo P, Golub TR, Mesirov JP. Subclass mapping: identifying common subtypes in independent disease data sets. PLoS One. 2007; 2: e1195.

21. Roh W, Chen P-L, Reuben A, Spencer CN, Prieto PA, Miller JP, et al. Integrated molecular analysis of tumor biopsies on sequential CTLA-4 and PD-1 blockade reveals markers of response and resistance. Sci Transl Med. 2017; 9.
