# Supplementary material for: MLKL and other necroptosis-related genes promote the tumor immune cell infiltration, guiding for the administration of immunotherapy in bladder urothelial carcinoma
Source: Apoptosis. 2023 Mar 31;28(5-6):892–911. doi: 10.1007/s10495-023-01830-8 (PMC10232593; doi:10.1007/s10495-023-01830-8)
Supplement: Supplementary file 21 — Supplementary file21 (DOCX 18 KB) [file 10495_2023_1830_MOESM21_ESM.docx]

**Supplementary Table 13. Relationship between CD8 expression and clinicopathological parameters**

| **Parameters** | **N** | **CD8 expression** | | **P value** |
| --- | --- | --- | --- | --- |
|  |  | **High** | **Low** |  |
| **Age** |  |  |  | ns |
| ＜60 | **1** | **1** | **0** |  |
| ≥60 | **7** | **3** | **4** |  |
| **Gender** |  |  |  | ns |
| male | **5** | **3** | **2** |  |
| female | **3** | **1** | **2** |  |
| **Tumor size (cm)** |  |  |  | ns |
| ＜3 | **5** | **2** | **3** |  |
| ≥3 | **3** | **2** | **1** |  |
| **Multiple neoplasms** |  |  |  | ns |
| single | **3** | **1** | **2** |  |
| multiple | **5** | **3** | **2** |  |
| **TNM stage** |  |  |  |  |
| **T** |  |  |  | ns |
| pTa-pT1 | **2** | **0** | **2** |  |
| pT2-pT4 | **6** | **4** | **2** |  |
| **N** |  |  |  | ns |
| yes | **6** | **3** | **3** |  |
| no | **2** | **1** | **1** |  |
| **M** |  |  |  | ns |
| yes | **1** | **1** | **0** |  |
| no | **7** | **3** | **4** |  |
| **Histologic grade** |  |  |  | ns |
| low | **5** | **2** | **3** |  |
| medium to high | **3** | **2** | **1** |  |
