# Supplementary material for: MLKL and other necroptosis-related genes promote the tumor immune cell infiltration, guiding for the administration of immunotherapy in bladder urothelial carcinoma
Source: Apoptosis. 2023 Mar 31;28(5-6):892–911. doi: 10.1007/s10495-023-01830-8 (PMC10232593; doi:10.1007/s10495-023-01830-8)
Supplement: Supplementary file 22 — Supplementary file22 (DOC 225 KB) [file 10495_2023_1830_MOESM22_ESM.doc]

**R Codes**

1.

inputFile="panGeneExp.txt"

setwd("")

rt=read.table(inputFile,sep="\t",header=T,check.names=F,row.names=1)

rt=rt[(rt[,"Type"]=="Tumor"),]

data=rt[,(1:(ncol(rt)-2))]

geneNum=ncol(data)

pdf(file="boxplot.pdf",width=8,height=5)

par(mar=c(3, 4, 1, 1))

boxplot(data,ylab ="Gene expression",col = rainbow(geneNum),xaxt = "n",outline = FALSE)

text(1:geneNum, par("usr")[3]-0.25, srt=45, adj=1, labels=colnames(data), xpd=TRUE, cex=0.5)

dev.off()

2. library(pheatmap)

inputFile="panGeneExp.txt"

setwd("")

rt=read.table(inputFile,header=T,sep="\t",check.names=F,row.names=1)

logFcTab=data.frame()

pTab=data.frame()

genelist=colnames(rt[,(1:(ncol(rt)-2))])

for(cancerType in levels(factor(rt[,"CancerType"]))){

data=rt[(rt[,"CancerType"]==cancerType),]

normal=data[(data[,"Type"]=="Normal"),]

tumor=data[(data[,"Type"]=="Tumor"),]

if(nrow(normal)>=5){

logFcVector=data.frame(cancerType)

pVector=data.frame(cancerType)

normal=normal[,(1:(ncol(normal)-2))]

tumor=tumor[,(1:(ncol(tumor)-2))]

for(gene in colnames(tumor)){

logFC=mean(tumor[,gene])-mean(normal[,gene])

test=wilcox.test(tumor[,gene],normal[,gene])

pVector=cbind(pVector,test$p.value)

logFcVector=cbind(logFcVector,logFC)

}

logFcTab=rbind(logFcTab,logFcVector)

pTab=rbind(pTab,pVector)

}

}

colnames(logFcTab)=c("CancerType",genelist)

write.table(logFcTab,file="logFC.txt",sep="\t",row.names=F,quote=F)

colnames(pTab)=c("CancerType",genelist)

write.table(pTab,file="pvalue.txt",sep="\t",row.names=F,quote=F)

row.names(logFcTab)=logFcTab[,1]

logFcTab=logFcTab[,-1]

pdf(file="heatmap.pdf",height=6,width=8)

pheatmap(logFcTab,

color = colorRampPalette(c("navy", "white", "firebrick3"))(50),

fontsize = 6,

fontsize_row=6,

fontsize_col=6,

treeheight_row = 0, border_color = "black")

dev.off()

3.

library(corrplot)

inputFile="panGeneExp.txt"

setwd("")

rt=read.table(inputFile,sep="\t",header=T,check.names=F,row.names=1)

rt=rt[(rt[,"Type"]=="Tumor"),]

data=rt[,(1:(ncol(rt)-2))]

geneNum=ncol(data)

res1=cor.mtest(data, conf.level = 0.95)

pdf("corrplot.pdf",height=7,width=7.5)

par(oma=c(0.1,0.1,0.1,0.1))

M=cor(data)

write.table(M, "cor.txt", quote = F, sep = "\t", row.names = T)

corrplot(M, order = "FPC", type = "upper", tl.cex=0.6, tl.col="black", tl.pos = "lt", number.cex = 0.8, col=colorRampPalette(c("#053061", "#2166AC", "#4393C3", "#92C5DE", "#D1E5F0", "#FFFFFF", "#FDDBC7", "#F4A582", "#D6604D", "#B2182B", "#67001F"))(100))

corrplot(M, add = TRUE, type = "lower", method = "ellipse", order = "FPC",pch=T,

p.mat = res1$p,

insig = "label_sig",

pch.cex = 1.0,

sig.level=0.05, tl.cex=0.6, tl.col="black",

diag = FALSE, tl.pos = "n", cl.pos = "n", number.cex = 0.8, col=colorRampPalette(c("#053061", "#2166AC", "#4393C3", "#92C5DE", "#D1E5F0", "#FFFFFF", "#FDDBC7", "#F4A582", "#D6604D", "#B2182B", "#67001F"))(100))

dev.off()

4.

expFile="panGeneExp.txt"

setwd("")

files=dir()

files=grep(".survival.tsv$",files,value=T)

surTime=data.frame()

for(i in 1:length(files)){

inputFile=files[i]

rt=read.table(inputFile,header=T,sep="\t",check.names=F,row.names=1)

rt=rt[,c(3,1)]

surTime=rbind(surTime,rt)

}

colnames(surTime)=c("futime","fustat")

exp=read.table(expFile,sep="\t",header=T,check.names=F,row.names=1)

exp=exp[(exp[,"Type"]=="Tumor"),]

sameSample=intersect(row.names(surTime),row.names(exp))

surTime=surTime[sameSample,]

exp=exp[sameSample,]

surData=cbind(surTime,exp)

surData=cbind(id=row.names(surData),surData)

write.table(surData,file="expTime.txt",sep="\t",quote=F,row.names=F)

5.

library(survival)

setwd("")

rt=read.table("expTime.txt",header=T,sep="\t",check.names=F,row.names=1)

rt$futime=rt$futime/365

allGene=colnames(rt)[3:(ncol(rt)-2)]

allType=levels(factor(rt[,"CancerType"]))

pdf(file="forest-2.pdf", width = 15,height =10)

geneNum=length(allGene)

n=length(allType)

nRow=n+1

ylim=c(1,nRow)

layout(matrix(1:(geneNum+1),nc=geneNum+1))

forestCol=rainbow(geneNum)

xlim = c(0,1)

par(mar=c(4.5,1,2,1))

plot(1,xlim=xlim,ylim=ylim,type="n",axes=F,xlab="",ylab="")

text(0.5,n:1,allType,adj=0,cex=1.5)

i=0

for(gene in colnames(rt)[3:(ncol(rt)-2)]){

i=i+1

outTab=data.frame()

for(CancerType in levels(factor(rt[,"CancerType"]))){

rt1=rt[(rt[,"CancerType"]==CancerType),]

cox=coxph(Surv(futime, fustat) ~ rt1[,gene], data = rt1)

coxSummary = summary(cox)

coxCoef=coxSummary$coefficients

coxP=coxCoef[,"Pr(>|z|)"]

outTab=rbind(outTab,

cbind(cancer=CancerType,

HR=coxSummary$conf.int[,"exp(coef)"],

HR.95L=coxSummary$conf.int[,"lower .95"],

HR.95H=coxSummary$conf.int[,"upper .95"],

pvalue=coxP) )

}

outFile=paste0(gene,".cox.txt")

write.table(outTab,file=outFile,sep="\t",row.names=F,quote=F)

coxRT <- read.table(outFile,header=T,sep="\t",row.names=1,check.names=F)

hr <- sprintf("%.3f",coxRT$"HR")

hr[as.numeric(hr)<0.001]=0.001

hrLow <- sprintf("%.3f",coxRT$"HR.95L")

hrLow[as.numeric(hrLow)<0.0001]=0.0001

hrHigh <- sprintf("%.3f",coxRT$"HR.95H")

hrHigh[as.numeric(hrHigh)>1000]=1000

par(mar=c(4.5,1,2,1),mgp=c(2,0.5,0))

LOGindex = 10

hrLow = log(as.numeric(hrLow),LOGindex)

hrHigh = log(as.numeric(hrHigh),LOGindex)

hr = log(as.numeric(hr),LOGindex)

xlim = c(floor(min(hrLow,hrHigh)),ceiling(max(hrLow,hrHigh)))

plot(1,xlim=xlim,ylim=ylim,type="n",axes=F,ylab="",xaxs="i",xlab="")

arrows(as.numeric(hrLow),n:1,as.numeric(hrHigh),n:1,angle=90,code=3,length=0.01,col=forestCol[i],lwd=3)

segments(log(1,LOGindex), 0, x1 = log(1,LOGindex), y1 = n)

points(as.numeric(hr), n:1, pch = 15, col = forestCol[i], cex=2)

a1 = axis(1,labels=F,tick=F)

axis(1,a1,10^a1)

text(log(1,LOGindex),n+1,gene,cex=1.5,xpd=T)

if(i==round(geneNum/2,0)){

text(log(1,LOGindex),-1.6,"Hazard Ratio",cex=1.5,xpd=T)

}

}

dev.off()

6.

library(limma)

library(ggplot2)

library(reshape2)

expFile="panGeneExp.txt"

subtypeFile="Subtype_Immune_Model_Based.txt"

setwd("")

exp=read.table(expFile, header=T,sep="\t",row.names=1,check.names=F)

exp=exp[(exp[,"Type"]=="Tumor"),]

exp=as.matrix(exp[,1:(ncol(exp)-2)])

row.names(exp)=gsub(".$","",row.names(exp))

exp=avereps(exp)

subtype=read.table(subtypeFile, header=T,sep="\t",row.names=1,check.names=F)

sameSample=intersect(row.names(subtype),row.names(exp))

subtype=subtype[sameSample,]

subtype=gsub(".+Immune |\\)","",subtype)

exp=exp[sameSample,]

exp=cbind(as.data.frame(exp),subtype)

outTab=data.frame()

geneSig=c()

for(gene in colnames(exp)[1:(ncol(exp)-1)]){

rt1=exp[,c(gene,"subtype")]

colnames(rt1)=c("expression","subtype")

ks=kruskal.test(expression ~ subtype, data = rt1)

p=ks$p.value

outTab=rbind(outTab,cbind(gene,pvalue=p))

Sig=ifelse(p<0.001,"***",ifelse(p<0.01,"**",ifelse(p<0.05,"*","")))

geneSig=c(geneSig,Sig)

}

geneSig=c(geneSig,"")

colnames(exp)=paste0(colnames(exp),geneSig)

write.table(outTab,file="immuneType.xls",sep="\t",row.names=F,quote=F)

data=melt(exp)

colnames(data)=c("Subtype","Gene","Expression")

p1=ggplot(data,aes(x=Subtype,

y=Expression,

fill=Subtype))+

guides(fill=guide_legend(title="Immune Subtype"))+

labs(x = "Immune Subtype", y = "Gene expression")+

geom_boxplot()+ facet_wrap(~Gene,nrow =2)+ theme_bw()

pdf(file="immuneType.pdf",width=8,height=6.5)

print(p1)

dev.off()

7. library(limma)

library(estimate)

setwd("")

files=dir()

files=grep("^symbol.",files,value=T)

outTab=data.frame()

for(i in files){

CancerType=gsub("symbol\\.|\\.txt","",i)

rt=read.table(i,sep="\t",header=T,check.names=F)

rt=as.matrix(rt)

rownames(rt)=rt[,1]

exp=rt[,2:ncol(rt)]

dimnames=list(rownames(exp),colnames(exp))

data=matrix(as.numeric(as.matrix(exp)),nrow=nrow(exp),dimnames=dimnames)

data=avereps(data)

group=sapply(strsplit(colnames(data),"\\-"),"[",4)

group=sapply(strsplit(group,""),"[",1)

group=gsub("2","1",group)

data=data[,group==0]

out=data[rowMeans(data)>0,]

out=rbind(ID=colnames(out),out)

write.table(out,file="uniq.symbol.txt",sep="\t",quote=F,col.names=F)

uniqFile="uniq.symbol.txt"

inputDs="commonGenes.gct"

outputDs="estimateScore.gct"

filterCommonGenes(input.f=uniqFile, output.f=inputDs, id="GeneSymbol")

estimateScore(input.ds =inputDs ,output.ds=outputDs)

scores=read.table("estimateScore.gct",skip = 2,header = T)

rownames(scores)=scores[,1]

scores=t(scores[,3:ncol(scores)])

rownames(scores)=gsub("\\.","\\-",rownames(scores))

outTab=rbind(outTab,cbind(scores,CancerType))

file.remove(uniqFile)

file.remove(inputDs)

file.remove(outputDs)

}

out=cbind(ID=row.names(outTab),outTab)

write.table(out,file="estimateScores.txt",sep="\t",quote=F,row.names=F)

8.

library(corrplot)

expFile="panGeneExp.txt"

scoreFile="estimateScores.txt"

scoreType="ImmuneScore"

setwd("")

exp=read.table(expFile, header=T,sep="\t",row.names=1,check.names=F)

exp=exp[(exp[,"Type"]=="Tumor"),]

TME=read.table(scoreFile, header=T,sep="\t",row.names=1,check.names=F)

sameSample=intersect(row.names(TME),row.names(exp))

TME=TME[sameSample,]

exp=exp[sameSample,]

outTab=data.frame()

pTab=data.frame()

for(i in levels(factor(exp[,"CancerType"]))){

exp1=exp[(exp[,"CancerType"]==i),]

TME1=TME[(TME[,"CancerType"]==i),]

x=as.numeric(TME1[,scoreType])

pVector=data.frame(i)

outVector=data.frame(i)

genes=colnames(exp1)[1:(ncol(exp1)-2)]

for(j in genes){

y=as.numeric(exp1[,j])

corT=cor.test(x,y,method="spearman")

cor=corT$estimate

pValue=corT$p.value

pVector=cbind(pVector,pValue)

outVector=cbind(outVector,cor)

}

pTab=rbind(pTab,pVector)

outTab=rbind(outTab,outVector)

}

colNames=c("CancerType",colnames(exp1)[1:(ncol(exp1)-2)])

colnames(outTab)=colNames

write.table(outTab,file="estimateCor.cor.txt",sep="\t",row.names=F,quote=F)

colnames(pTab)=colNames

write.table(pTab,file="estimateCor.pval.txt",sep="\t",row.names=F,quote=F)

pdf(file=paste0(scoreType,".pdf"),height=3,width=7)

par(mar=c(0.2,0.5,0.1,0.5))

row.names(outTab)=outTab[,1]

outTab=outTab[,-1]

corrplot(corr=as.matrix(t(outTab)),

title=paste0("\n\n\n\n",scoreType),

col=colorRampPalette(c("blue", "white", "red"))(50))

dev.off()

9.

library(limma)

library(corrplot)

expFile="panGeneExp.txt"

scoreFile="StemnessScores_RNAexp_20170127.2.tsv"

scoreType="RNAss"

setwd("")

exp=read.table(expFile, header=T,sep="\t",row.names=1,check.names=F)

exp=exp[(exp[,"Type"]=="Tumor"),]

STEM=read.table(scoreFile, header=T,sep="\t",row.names=1,check.names=F)

STEM=t(STEM)

outTab=data.frame()

pTab=data.frame()

for(i in levels(factor(exp[,"CancerType"]))){

exp1=exp[(exp[,"CancerType"]==i),]

exp1=as.matrix(exp1[,1:(ncol(exp1)-2)])

row.names(exp1)=gsub(".$","",row.names(exp1))

exp1=avereps(exp1)

sameSample=intersect(row.names(STEM),row.names(exp1))

STEM1=STEM[sameSample,]

exp1=exp1[sameSample,]

x=as.numeric(STEM1[,scoreType])

pVector=data.frame(i)

outVector=data.frame(i)

genes=colnames(exp1)

for(j in genes){

y=as.numeric(exp1[,j])

corT=cor.test(x,y,method="spearman")

cor=corT$estimate

pValue=corT$p.value

pVector=cbind(pVector,pValue)

outVector=cbind(outVector,cor)

}

pTab=rbind(pTab,pVector)

outTab=rbind(outTab,outVector)

}

colNames=c("CancerType",colnames(exp)[1:(ncol(exp)-2)])

colnames(outTab)=colNames

write.table(outTab,file="RNAssCor.cor.txt",sep="\t",row.names=F,quote=F)

colnames(pTab)=colNames

write.table(pTab,file="RNAssCor.pval.txt",sep="\t",row.names=F,quote=F)

pdf("RNAssCor.pdf",height=3,width=7)

par(mar=c(0.2,0.5,0.1,0.5))

row.names(outTab)=outTab[,1]

outTab=outTab[,-1]

corrplot(corr=as.matrix(t(outTab)),

title=paste0("\n\n\n\n",scoreType),

col=colorRampPalette(c("blue", "white", "red"))(50))

dev.off()

10.

library(limma)

library(corrplot)

expFile="panGeneExp.txt"

scoreFile="StemnessScores_DNAmeth_20170210.tsv"

scoreType="DNAss"

setwd("")

exp=read.table(expFile, header=T,sep="\t",row.names=1,check.names=F)

exp=exp[(exp[,"Type"]=="Tumor"),]

STEM=read.table(scoreFile, header=T,sep="\t",row.names=1,check.names=F)

STEM=t(STEM)

outTab=data.frame()

pTab=data.frame()

for(i in levels(factor(exp[,"CancerType"]))){

exp1=exp[(exp[,"CancerType"]==i),]

exp1=as.matrix(exp1[,1:(ncol(exp1)-2)])

row.names(exp1)=gsub(".$","",row.names(exp1))

exp1=avereps(exp1)

sameSample=intersect(row.names(STEM),row.names(exp1))

STEM1=STEM[sameSample,]

exp1=exp1[sameSample,]

x=as.numeric(STEM1[,scoreType])

pVector=data.frame(i)

outVector=data.frame(i)

genes=colnames(exp1)

for(j in genes){

y=as.numeric(exp1[,j])

corT=cor.test(x,y,method="spearman")

cor=corT$estimate

pValue=corT$p.value

pVector=cbind(pVector,pValue)

outVector=cbind(outVector,cor)

}

pTab=rbind(pTab,pVector)

outTab=rbind(outTab,outVector)

}

colNames=c("CancerType",colnames(exp)[1:(ncol(exp)-2)])

colnames(outTab)=colNames

write.table(outTab,file="DNAssCor.cor.txt",sep="\t",row.names=F,quote=F)

colnames(pTab)=colNames

write.table(pTab,file="DNAssCor.pval.txt",sep="\t",row.names=F,quote=F)

pdf("DNAssCor.pdf",height=3,width=7)

par(mar=c(0.2,0.5,0.1,0.5))

row.names(outTab)=outTab[,1]

outTab=outTab[,-1]

corrplot(corr=as.matrix(t(outTab)),

title=paste0("\n\n\n\n",scoreType),

col=colorRampPalette(c("blue", "white", "red"))(50))

dev.off()

11.

library(impute)

library(limma)

library(ggplot2)

library(ggpubr)

drugFile="drug.txt"

expFile="geneExp.txt"

geneFile="gene.txt"

corPlotNum=25

setwd("C:\\Users\\zbq123\\Desktop\\bladder-pan-cancer\\18")

rt=read.table(drugFile,sep="\t",header=T,check.names=F, quote = "")

rt=as.matrix(rt)

rownames(rt)=rt[,1]

drug=rt[,2:ncol(rt)]

dimnames=list(rownames(drug),colnames(drug))

data=matrix(as.numeric(as.matrix(drug)),nrow=nrow(drug),dimnames=dimnames)

mat=impute.knn(data)

drug=mat$data

drug=avereps(drug)

rt=read.table(expFile,sep="\t",header=T,check.names=F)

rt=as.matrix(rt)

rownames(rt)=rt[,1]

exp=rt[,2:ncol(rt)]

dimnames=list(rownames(exp),colnames(exp))

data=matrix(as.numeric(as.matrix(exp)),nrow=nrow(exp),dimnames=dimnames)

exp=avereps(data)

gene=read.table(geneFile,sep="\t",header=F,check.names=F)

genelist=as.vector(gene[,1])

genelist=gsub(" ","",genelist)

genelist=intersect(genelist,row.names(exp))

exp=exp[genelist,]

outTab=data.frame()

for(Gene in row.names(exp)){

x=as.numeric(exp[Gene,])

for(Drug in row.names(drug)){

y=as.numeric(drug[Drug,])

corT=cor.test(x,y,method="pearson")

cor=corT$estimate

pvalue=corT$p.value

if(pvalue<0.05){

outVector=cbind(Gene,Drug,cor,pvalue)

outTab=rbind(outTab,outVector)

}

}

}

outTab=outTab[order(as.numeric(as.vector(outTab$pvalue))),]

write.table(outTab,file="drugCor.txt",sep="\t",row.names=F,quote=F)

plotList=list()

if(nrow(outTab)<corPlotNum){

corPlotNum=nrow(outTab)

}

for(i in 1:corPlotNum){

Gene=outTab[i,1]

Drug=outTab[i,2]

x=as.numeric(exp[Gene,])

y=as.numeric(drug[Drug,])

cor=sprintf("%.03f",as.numeric(outTab[i,3]))

pvalue=0

if(as.numeric(outTab[i,4])<0.001){

pvalue="p<0.001"

}else{

pvalue=paste0("p=",sprintf("%.03f",as.numeric(outTab[i,4])))

}

df1=as.data.frame(cbind(x,y))

p1=ggplot(data = df1, aes(x = x, y = y))+

geom_point(size=1)+

stat_smooth(method="lm",se=FALSE, formula=y~x)+

labs(x="",y="",title = paste0(Gene,", ",Drug),subtitle = paste0("Cor=",cor,", ",pvalue))+

theme(axis.ticks = element_blank(), axis.text.y = element_blank(),axis.text.x = element_blank())+

theme_bw()

plotList[[i]]=p1

}

nrow=ceiling(sqrt(corPlotNum))

ncol=ceiling(corPlotNum/nrow)

pdf(file="drugCor.pdf", width = 13,height =9)

ggarrange(plotlist=plotList,nrow=nrow,ncol=ncol)

dev.off()

12.

library(limma)

library(ggplot2)

library(reshape2)

cancerType="BLCA"

expFile="panGeneExp.txt"

subtypeFile="Subtype_Immune_Model_Based.txt"

setwd("")

exp=read.table(expFile, header=T,sep="\t",row.names=1,check.names=F)

exp=exp[(exp[,"Type"]=="Tumor"),]

exp=exp[(exp[,"CancerType"]==cancerType),]

exp=as.matrix(exp[,1:(ncol(exp)-2)])

row.names(exp)=gsub(".$","",row.names(exp))

exp=avereps(exp)

subtype=read.table(subtypeFile, header=T,sep="\t",row.names=1,check.names=F)

sameSample=intersect(row.names(subtype),row.names(exp))

subtype=subtype[sameSample,]

subtype=gsub(".+Immune |\\)","",subtype)

exp=exp[sameSample,]

exp=cbind(as.data.frame(exp),subtype)

outTab=data.frame()

geneSig=c()

for(gene in colnames(exp)[1:(ncol(exp)-1)]){

rt1=exp[,c(gene,"subtype")]

colnames(rt1)=c("expression","subtype")

ks=kruskal.test(expression ~ subtype, data = rt1)

p=ks$p.value

outTab=rbind(outTab,cbind(gene,pvalue=p))

Sig=ifelse(p<0.001,"***",ifelse(p<0.01,"**",ifelse(p<0.05,"*","")))

geneSig=c(geneSig,Sig)

}

geneSig=c(geneSig,"")

colnames(exp)=paste0(colnames(exp),geneSig)

write.table(outTab,file="immuneType.result.xls",sep="\t",row.names=F,quote=F)

data=melt(exp)

colnames(data)=c("Subtype","Gene","Expression")

p1=ggplot(data,aes(x=Subtype,

y=Expression,

fill=Subtype))+

ggtitle(paste0("Cancer: ",cancerType))+theme(title=element_text(size=10))+

guides(fill=guide_legend(title="Immune Subtype"))+

labs(x = "Immune Subtype", y = "Gene expression")+

geom_boxplot()+ facet_wrap(~Gene,nrow =2)+ theme_bw()

pdf(file="immuneType.pdf",width=8,height=6.5)

print(p1)

dev.off()

13.

library(limma)

library(ggplot2)

library(reshape2)

cancerType="BLCA"

expFile="panGeneExp.txt"

cliFile="clinical.txt"

setwd("")

exp=read.table(expFile, header=T,sep="\t",row.names=1,check.names=F)

exp=exp[(exp[,"Type"]=="Tumor"),]

exp=exp[(exp[,"CancerType"]==cancerType),]

exp=as.matrix(exp[,1:(ncol(exp)-2)])

cli=read.table(cliFile, header=T,sep="\t",row.names=1,check.names=F)

cliName=colnames(cli)[1]

sameSample=intersect(row.names(cli),row.names(exp))

cli=cli[sameSample,]

exp=exp[sameSample,]

exp=cbind(as.data.frame(exp),cli)

outTab=data.frame()

geneSig=c()

for(gene in colnames(exp)[1:(ncol(exp)-1)]){

rt1=exp[,c(gene,"cli")]

colnames(rt1)=c("expression","cli")

ks=kruskal.test(expression ~ cli, data = rt1)

p=ks$p.value

outTab=rbind(outTab,cbind(gene,pvalue=p))

Sig=ifelse(p<0.001,"***",ifelse(p<0.01,"**",ifelse(p<0.05,"*","")))

geneSig=c(geneSig,Sig)

}

geneSig=c(geneSig,"")

colnames(exp)=paste0(colnames(exp),geneSig)

write.table(outTab,file="cliCor.result.xls",sep="\t",row.names=F,quote=F)

data=melt(exp)

colnames(data)=c("cli","Gene","Expression")

p1=ggplot(data,aes(x=cli,

y=Expression,

fill=cli))+

ggtitle(paste0("Cancer: ",cancerType))+theme(title=element_text(size=10))+

guides(fill=guide_legend(title=cliName))+

labs(x = cliName, y = "Gene expression")+

geom_boxplot()+ facet_wrap(~Gene,nrow =2)+ theme_bw()+

theme(axis.text.x = element_text(angle = 45, hjust = 1))

pdf(file="cliCor.pdf",width=8,height=6.5)

print(p1)

dev.off()

14.

library(reshape2)

library(ggplot2)

library(ggpubr)

library(limma)

cancerType="BLCA"

expFile="panGeneExp.txt"

tmeFile="estimateScores.txt"

RNAssFile="StemnessScores_RNAexp_20170127.2.tsv"

DNAssFile="StemnessScores_DNAmeth_20170210.tsv"

setwd("")

exp=read.table(expFile, header=T,sep="\t",row.names=1,check.names=F)

exp=exp[(exp[,"Type"]=="Tumor"),]

exp=exp[(exp[,"CancerType"]==cancerType),]

exp=as.matrix(exp[,1:(ncol(exp)-2)])

row.names(exp)=gsub(".$","",row.names(exp))

exp=avereps(exp)

TME=read.table(tmeFile, header=T,sep="\t",row.names=1,check.names=F)

TME=as.matrix(TME[,1:3])

row.names(TME)=gsub(".$","",row.names(TME))

TME=avereps(TME)

RNAss=read.table(RNAssFile, header=T,sep="\t",row.names=1,check.names=F)

RNAss=t(RNAss[1:2,])

DNAss=read.table(DNAssFile, header=T,sep="\t",row.names=1,check.names=F)

DNAss=t(DNAss[1:2,])

sameSample=Reduce(intersect,list(row.names(TME),row.names(exp),row.names(DNAss),row.names(RNAss)))

exp=exp[sameSample,]

TME=TME[sameSample,]

RNAss=RNAss[sameSample,]

DNAss=DNAss[sameSample,]

data=cbind(RNAss,DNAss,TME,exp)

data=as.data.frame(data[,c(-2,-4)])

scoreData=data[,1:5]

expData=data[,6:ncol(data)]

scoreData=cbind(ID=row.names(scoreData),scoreData)

expData=cbind(ID=row.names(expData),expData)

expMelt = melt(expData,id="ID")

colnames(expMelt) = c('ID','gene','exp')

scoreMelt = melt(scoreData,id="ID")

colnames(scoreMelt) = c('ID','TME','score')

mergedata = merge(expMelt,scoreMelt,by="ID")

pdf("cor.pdf",height=6,width=13)

ggplot(data = mergedata, aes(x = exp, y = score))+

facet_grid(TME~gene,scales = "free")+geom_point(size=0.25)+

stat_smooth(method="lm",se=FALSE,formula=y~x)+

labs(x="Gene expression",y="")+

ggtitle(paste0("Cancer: ",cancerType))+theme(title=element_text(size=10))+

theme(axis.ticks = element_blank(), axis.text.y = element_blank(),axis.text.x = element_blank())+

stat_cor(method = 'spearman', aes(x =exp, y =score),label.sep = ",",cex=2)

dev.off()

15.

library(limma)

gene=" "

setwd("D:\\biowolf\\panCancer\\23.geneCor")

geneRT=read.table("gene.txt",sep="\t",header=F)

files=dir()

files=grep("^symbol.",files,value=T)

outTab=data.frame()

corTab=data.frame()

sameGenes=c()

for(i in files){

CancerType=gsub("^symbol\\.|\\.txt$","",i)

rt=read.table(i, header=T,sep="\t",check.names=F)

rt=as.matrix(rt)

rownames(rt)=rt[,1]

exp=rt[,2:ncol(rt)]

dimnames=list(rownames(exp),colnames(exp))

data=matrix(as.numeric(as.matrix(exp)),nrow=nrow(exp),dimnames=dimnames)

data=avereps(data)

group=sapply(strsplit(colnames(data),"\\-"),"[",4)

group=sapply(strsplit(group,""),"[",1)

group=gsub("2","1",group)

data=data[,group==0]

sameGenes=intersect(as.vector(geneRT[,1]),row.names(data))

data=data[c(sameGenes,gene),]

x=as.numeric(data[gene,])

outVector=data.frame(CancerType)

corVector=data.frame(CancerType)

for(j in sameGenes){

y=as.numeric(data[j,])

if(sd(y)>0.01){

corT=cor.test(x,y)

cor=corT$estimate

pValue=corT$p.value

outVector=cbind(outVector,pValue)

corVector=cbind(corVector,cor)

}

else{

outVector=cbind(outVector,pValue=1)

corVector=cbind(corVector,cor=0)

}

}

outTab=rbind(outTab,outVector)

corTab=rbind(corTab,corVector)

}

colnames(outTab)=c("CancerType",sameGenes)

write.table(outTab,file="geneCor.pvalue.txt",sep="\t",row.names=F,quote=F)

colnames(corTab)=c("CancerType",sameGenes)

write.table(corTab,file="geneCor.cor.txt",sep="\t",row.names=F,quote=F)

16.

library(reshape2)

library(RColorBrewer)

options(stringsAsFactors = F)

setwd("")

gene="MLKL"

up <- read.table("geneCor.pvalue.txt",sep = "\t",check.names = F,header = T,row.names=1)

dn <- read.table("geneCor.cor.txt",sep = "\t",check.names = F,header = T,row.names=1)

dn=t(dn)

up=t(up)

colVector=c("#AB221F","#3878C1","#FFFADD")

gene.level <- as.character(rownames(dn))

cancer.level <- as.character(colnames(dn))

dn.long <- setNames(melt(dn), c('Gene', 'Cancer', 'Frequency'))

dn.long$Categrory <- "DN"

up.long <- setNames(melt(up), c('Gene', 'Cancer', 'Frequency'))

up.long$Categrory <- "UP"

dn.long$range <- cut(dn.long$Frequency,

breaks = seq(floor(min(dn.long$Frequency)),

ceiling(max(dn.long$Frequency)),0.01))

rangeMat1 <- levels(dn.long$range)

rbPal1 <- colorRampPalette(colors = c(colVector[3],"white",colVector[1]))

col.vec1 <- rbPal1(length(rangeMat1)); names(col.vec1) <- rangeMat1

dn.long$color <- col.vec1[as.character(dn.long$range)]

up.long$range <- cut(up.long$Frequency, breaks = seq(floor(min(up.long$Frequency)),ceiling(max(up.long$Frequency)),0.01))

rangeMat2 <- levels(up.long$range)

rbPal2 <- colorRampPalette(colors = c(colVector[3],colVector[2]))

col.vec2 <- rbPal2(length(rangeMat2)); names(col.vec2) <- rangeMat2

up.long$color <- col.vec2[as.character(up.long$range)]

heatmat <- rbind.data.frame(dn.long,up.long)

pdf(paste0("heatmap-",gene,".pdf"),width = 7,height = 6)

layout(mat=matrix(c(1,0,1,2,1,0,1,3,1,0),5,2,byrow=T),widths=c(length(cancer.level),2))

par(bty="n", mgp = c(2,0.5,0), mar = c(5.1, 5.5, 3, 3),tcl=-.25,xpd = T)

x=as.numeric(factor(heatmat$Cancer,levels = cancer.level))

y=as.numeric(factor(heatmat$Gene,levels = gene.level))

plot(1,xlim=c(1,length(unique(x))+1),ylim=c(1,length(unique(y))+1),

xaxs="i", yaxs="i",xaxt="n",yaxt="n",

type="n",bty="n",xlab="",ylab="",

main = "Coexpression between MLKL and Immune Checkpoint",cex.main=2)

for(i in 1:nrow(heatmat)) {

if(heatmat$Categrory[i]=="DN") polygon(x[i]+c(0,1,1),y[i]+c(0,0,1),col=heatmat$color[i])

if(heatmat$Categrory[i]=="UP") {

polygon(x[i]+c(0,1,0),y[i]+c(0,1,1),col=heatmat$color[i])

if(heatmat$Frequency[i]<0.001){

text(x[i]+0.5,y[i]+0.8,"***",cex=0.8)

}else if(heatmat$Frequency[i]<0.01){

text(x[i]+0.5,y[i]+0.8,"**",cex=0.8)

}else if(heatmat$Frequency[i]<0.05){

text(x[i]+0.5,y[i]+0.8,"*",cex=0.8)

}

}

}

axis(1,at = sort(unique(x)) + 0.5,labels = cancer.level,lty = 0,las = 2)

axis(2,at = sort(unique(y)) + 0.5,labels = gene.level,lty = 0,las = 1)

mtext("Cancer types",side = 1,line = 3.5,cex=1.2)

par(mar=c(0,0,0,2),xpd = T,cex.axis=1.6)

barplot(rep(1,length(col.vec2)),border = NA, space = 0,ylab="",xlab="",ylim=c(1,length(col.vec2)),horiz=TRUE,

axes = F, col=col.vec2) # Loss

axis(4,at=c(1,ceiling(length(col.vec2)/2),length(col.vec2)),c(round(min(up),1),'Pvalue',round(max(up),1)),tick=FALSE)

par(mar=c(0,0,0,2),xpd = T,cex.axis=1.6)

barplot(rep(1,length(col.vec1)),border = NA, space = 0,ylab="",xlab="",ylim=c(1,length(col.vec1)),horiz=TRUE,

axes = F, col=col.vec1) # Gain

axis(4,at=c(1,ceiling(length(col.vec1)/2),length(col.vec1)),c(round(min(dn),1),'Cor',round(max(dn),1)),tick=FALSE)

dev.off()

17.

library(limma)

gene="RIPK3"

setwd("")

files=dir()

files=grep("^symbol.",files,value=T)

immunes=dir()

immunes=grep("^ssGSEA.",immunes,value=T)

outTab=data.frame()

corTab=data.frame()

for(i in files){

CancerType=gsub("^symbol\\.|\\.txt$","",i)

rt=read.table(i, header=T,sep="\t",check.names=F)

rt=as.matrix(rt)

rownames(rt)=rt[,1]

data=rt[,2:ncol(rt)]

group=sapply(strsplit(colnames(data),"\\-"),"[",4)

group=sapply(strsplit(group,""),"[",1)

group=gsub("2","1",group)

data=data[,group==0]

data=data[gene,]

data=as.data.frame(data)

data=t(data)

row.names(data)=gene

for(k in immunes)

{ImCancerType=gsub("^ssGSEA.result\\-|\\.txt$","",k)

if(CancerType == ImCancerType){

CELL=read.table(k, header=T,sep="\t",check.names=F,quote="",row.names = 1)

samecell=row.names(CELL)

data2=rbind(data,CELL)

x=as.numeric(data2[gene,])

outVector=data.frame(CancerType)

corVector=data.frame(CancerType)

for(j in samecell){

y=as.numeric(data2[j,])

if(sd(y)>0.01){

corT=cor.test(x,y)

cor=corT$estimate

pValue=corT$p.value

outVector=cbind(outVector,pValue)

corVector=cbind(corVector,cor)

}

else{

outVector=cbind(outVector,pValue=1)

corVector=cbind(corVector,cor=0)

}

}

outTab=rbind(outTab,outVector)

corTab=rbind(corTab,corVector)

}}}

colnames(outTab)=c("CancerType",samecell)

write.table(outTab,file="geneCor.pvalue.txt",sep="\t",row.names=F,quote=F)

colnames(corTab)=c("CancerType",samecell)

write.table(corTab,file="geneCor.cor.txt",sep="\t",row.names=F,quote=F)

18.

library(BLCAsubtyping)

setwd("")

expFile="merge.txt"

exp=read.table(expFile, header=T, sep="\t", check.names=F, row.names=1)

cl <- classify(expMat = exp, classification.systems = c("Baylor", "UNC", "MDA", "CIT", "Lund", "TCGA"))

write.table(cl, file="subtype.txt", sep="\t", row.names=F, quote=F)

19.

setwd("")

library(MCPcounter)

expFile="merge.txt"

exp=read.table(expFile, header=T, sep="\t", check.names=F, row.names=1)

estimate <- MCPcounter.estimate(exp, featuresType = "HUGO_symbols")

write.table(estimate, file="immunescore.txt", sep="\t", row.names=T, quote=F)

20.

library(consensusMIBC)

setwd("")

expFile="merge.txt"

exp=read.table(expFile, header=T, sep="\t", check.names=F, row.names=1)

sample_classes <- getConsensusClass(exp, minCor = .2, gene_id = "hgnc_symbol")

write.table(sample_classes, file="mibc-subtype.txt", sep="\t", row.names=T, quote=F)

21.

library(classifyNMIBC)

setwd("")

expFile="merge.txt"

exp=read.table(expFile, header=T, sep="\t", check.names=F, row.names=1)

sample_classes=classifyNMIBC(exp,minCor = 0.2,gene_id = c("hgnc_symbol")[1])

write.table(sample_classes, file="nmibc-subtype.txt", sep="\t", row.names=T, quote=F)

22.

library(immunedeconv)

library(tidyverse)

setwd("")

exprMatrix <- read.table("merge.txt", header=TRUE, sep="\t", check.names=F, row.names=1, as.is=TRUE)

res <- deconvolute(exprMatrix, method="timer", indications = rep("BLCA", 1841))

write.table(res, "timer.txt", sep="\t", col.names=T, row.names=F, quote=F)

23.

library(xCell)

setwd("")

exprMatrix = read.table("merge.txt",header=TRUE,row.names=1, sep="\t", check.names=F, as.is=TRUE)

xCell=xCellAnalysis(exprMatrix)

write.table(xCell, file="xcellimmunescore.txt", sep="\t", row.names=T, quote=F)

24.

inputFile="cnvMatrix.txt"

setwd("")

rt=read.table(inputFile, header=T, sep="\t", check.names=F, row.names=1)

GAIN=rowSums(rt> 0)

LOSS=rowSums(rt< 0)

GAIN=GAIN/ncol(rt)*100

LOSS=LOSS/ncol(rt)*100

data=cbind(GAIN, LOSS)

data=data[order(data[,"GAIN"],decreasing = T),]

data.max = apply(data, 1, max)

pdf(file="CNVfreq.pdf", width=9, height=6)

cex=1.3

par(cex.lab=cex, cex.axis=cex, font.axis=2, las=1, xpd=T)

bar=barplot(data.max, col="grey80", border=NA,

xlab="", ylab="CNV.frequency(%)", space=1.5,

xaxt="n", ylim=c(0,1.2*max(data.max)))

points(bar,data[,"GAIN"], pch=20, col=2, cex=3)

points(bar,data[,"LOSS"], pch=20, col=3, cex=3)

legend("top", legend=c('GAIN','LOSS'), col=2:3, pch=20, bty="n", cex=2, ncol=2)

par(srt=45)

text(bar, par('usr')[3]-0.2, rownames(data), adj=1)

dev.off()

25.

library("RCircos")

setwd("")

cytoBandIdeogram=read.table("refer.txt", header=T, sep="\t")

chr.exclude <- NULL

cyto.info <- cytoBandIdeogram

tracks.inside <- 5

tracks.outside <- 0

RCircos.Set.Core.Components(cyto.info, chr.exclude, tracks.inside, tracks.outside)

rcircos.params <- RCircos.Get.Plot.Parameters()

rcircos.params$text.size=1

rcircos.params$point.size=5

RCircos.Reset.Plot.Parameters(rcircos.params)

pdf(file="RCircos.pdf", width=8, height=8)

RCircos.Set.Plot.Area()

RCircos.Chromosome.Ideogram.Plot()

RCircos.Scatter.Data=read.table("Rcircos.scatter.txt", header=T, sep="\t", check.names=F)

data.col <- 4

track.num <- 1

side <- "in"

RCircos.Scatter.Plot(RCircos.Scatter.Data, data.col, track.num, side, by.fold=0.1)

RCircos.Gene.Label.Data=read.table("Rcircos.geneLabel.txt", header=T, sep="\t", check.names=F)

name.col <- 4

side <- "in"

track.num <- 2

RCircos.Gene.Connector.Plot(RCircos.Gene.Label.Data, track.num, side)

track.num <- 3

RCircos.Gene.Name.Plot(RCircos.Gene.Label.Data, name.col, track.num, side)

dev.off()

26.

library(limma)

library(sva)

setwd("")

files=c("", "")

geneList=list()

for(i in 1:length(files)){

inputFile=files[i]

rt=read.table(inputFile, header=T, sep="\t",check.names=F)

header=unlist(strsplit(inputFile, "\\.|\\-"))

geneList[[header[1]]]=as.vector(rt[,1])

}

intersectGenes=Reduce(intersect, geneList)

allTab=data.frame()

batchType=c()

for(i in 1:length(files)){

inputFile=files[i]

header=unlist(strsplit(inputFile, "\\.|\\-"))

rt=read.table(inputFile, header=T, sep="\t", check.names=F)

rt=as.matrix(rt)

rownames(rt)=rt[,1]

exp=rt[,2:ncol(rt)]

dimnames=list(rownames(exp),colnames(exp))

data=matrix(as.numeric(as.matrix(exp)),nrow=nrow(exp),dimnames=dimnames)

rt=avereps(data)

colnames(rt)=paste0(header[1], "_", colnames(rt))

if(header[1] == "TCGA"){

group=sapply(strsplit(colnames(rt),"\\-"), "[", 4)

group=sapply(strsplit(group,""), "[", 1)

rt=rt[,group==0]

rt=t(rt)

row.names(rt)=gsub("(.*?)\\-(.*?)\\-(.*?)\\-.*", "\\1\\-\\2\\-\\3", row.names(rt))

rt=avereps(rt)

rt=t(rt)

}

qx=as.numeric(quantile(rt, c(0, 0.25, 0.5, 0.75, 0.99, 1.0), na.rm=T))

LogC=( (qx[5]>100) || ( (qx[6]-qx[1])>50 && qx[2]>0) )

if(LogC){

rt[rt<0]=0

rt=log2(rt+1)}

if(header[1] != "TCGA"){

rt=normalizeBetweenArrays(rt)

}

if(i==1){

allTab=rt[intersectGenes,]

}else{

allTab=cbind(allTab, rt[intersectGenes,])

}

batchType=c(batchType, rep(i,ncol(rt)))

}

outTab=ComBat(allTab, batchType, par.prior=TRUE)

outTab=rbind(geneNames=colnames(outTab), outTab)

write.table(outTab, file="merge.txt", sep="\t", quote=F, col.names=F)

27.

library(limma)

expFile="merge.txt"

geneFile="gene.txt"

setwd("")

rt=read.table(expFile, header=T, sep="\t", check.names=F)

rt=as.matrix(rt)

rownames(rt)=rt[,1]

exp=rt[,2:ncol(rt)]

dimnames=list(rownames(exp),colnames(exp))

data=matrix(as.numeric(as.matrix(exp)),nrow=nrow(exp),dimnames=dimnames)

data=avereps(data)

data=data[rowMeans(data)>0,]

gene=read.table(geneFile, header=T, sep="\t", check.names=F)

sameGene=intersect(as.vector(gene[,1]), rownames(data))

geneExp=data[sameGene,]

out=rbind(ID=colnames(geneExp),geneExp)

write.table(out,file="m6aGeneExp.txt",sep="\t",quote=F,col.names=F)

28.

library(maftools)

setwd("")

geneRT=read.table("gene.txt", header=T, sep="\t", check.names=F, row.names=1)

gene=row.names(geneRT)

pdf(file="oncoplot.pdf", width=7, height=6)

maf=read.maf(maf="input.maf")

oncoplot(maf=maf, genes=gene, draw_titv=T)

dev.off()

29.

library(limma)

library(ggpubr)

expFile="m6aGeneExp.txt"

mutFile="mutMatrix.txt"

mutGene=""

setwd("")

exp=read.table(expFile, header=T, sep="\t", check.names=F, row.names=1)

colnames(exp)=gsub("(.*?)\\_(.*?)", "\\2", colnames(exp))

exp=t(exp)

mut=read.table(mutFile, header=T, sep="\t", check.names=F, row.names=1)

mut=t(mut[mutGene,,drop=F])

colnames(mut)=c("Type")

sameSample=intersect(row.names(mut), row.names(exp))

mut=mut[sameSample,,drop=F]

exp=exp[sameSample,,drop=F]

data=cbind(as.data.frame(exp), as.data.frame(mut))

data$Type=paste0(mutGene, " " , data$Type)

data$Type=factor(data$Type, levels=c(paste0(mutGene, " Wild"), paste0(mutGene, " Mutation")) )

group=levels(factor(data$Type))

comp=combn(group,2)

my_comparisons=list()

for(i in 1:ncol(comp)){my_comparisons[[i]]<-comp[,i]}

for(gene in colnames(data)[1:(ncol(data)-1)]){

data1=data[,c(gene, "Type")]

colnames(data1)=c("expression", "Type")

boxplot=ggboxplot(data1, x="Type", y="expression", fill="Type",

xlab="",

ylab=paste0(gene, " expression"),

legend.title="",

palette=c("#0066FF", "#FF0000") )+

stat_compare_means(comparisons = my_comparisons)

pdf(file=paste0(mutGene, "(mut)_", gene,".pdf"), width=5, height=4.5)

print(boxplot)

dev.off()

}

30.

library(limma)

library(survival)

library(survminer)

expFile=" "

cliFile="time.txt"

setwd("")

rt=read.table(expFile, header=T, sep="\t", check.names=F)

rt=as.matrix(rt)

rownames(rt)=rt[,1]

exp=rt[,2:ncol(rt)]

dimnames=list(rownames(exp), colnames(exp))

data=matrix(as.numeric(as.matrix(exp)), nrow=nrow(exp), dimnames=dimnames)

data=avereps(data)

data=data[rowMeans(data)>0,]

data=t(data)

rownames(data)=gsub("(.*?)\\_(.*?)", "\\2", rownames(data))

cli=read.table(cliFile, header=T, sep="\t", check.names=F, row.names=1)

cli$futime=cli$futime/365

sameSample=intersect(row.names(data), row.names(cli))

data=data[sameSample,]

cli=cli[sameSample,]

rt=cbind(cli, data)

outTab=data.frame()

km=c()

for(i in colnames(rt[,3:ncol(rt)])){

cox <- coxph(Surv(futime, fustat) ~ rt[,i], data = rt)

coxSummary = summary(cox)

coxP=coxSummary$coefficients[,"Pr(>|z|)"]

outTab=rbind(outTab,

cbind(id=i,

HR=coxSummary$conf.int[,"exp(coef)"],

HR.95L=coxSummary$conf.int[,"lower .95"],

HR.95H=coxSummary$conf.int[,"upper .95"],

pvalue=coxSummary$coefficients[,"Pr(>|z|)"])

)

data=rt[,c("futime", "fustat", i)]

colnames(data)=c("futime", "fustat", "gene")

res.cut=surv_cutpoint(data, time = "futime", event = "fustat", variables =c("gene"))

res.cat=surv_categorize(res.cut)

fit=survfit(Surv(futime, fustat) ~gene, data = res.cat)

diff=survdiff(Surv(futime, fustat) ~gene,data =res.cat)

pValue=1-pchisq(diff$chisq, df=1)

km=c(km, pValue)

if(pValue<0.05){

if(pValue<0.001){

pValue="p<0.001"

}else{

pValue=paste0("p=",sprintf("%.03f",pValue))

}

surPlot=ggsurvplot(fit,

data=res.cat,

pval=pValue,

pval.size=6,

legend.title=i,

legend.labs=c("high","low"),

xlab="Time(years)",

ylab="Overall survival",

palette=c("red", "blue"),

break.time.by=1,

conf.int=T,

risk.table=TRUE,

risk.table.title="",

risk.table.height=.25)

pdf(file=paste0("sur.", i, ".pdf"),onefile = FALSE,

width = 6,

height =5)

print(surPlot)

dev.off()

data.survdiff <- survdiff(Surv(futime, fustat) ~gene,data =res.cat)

p.val = 1 - pchisq(data.survdiff$chisq, length(data.survdiff$n) - 1)

HR = (data.survdiff$obs[1]/data.survdiff$exp[1])/(data.survdiff$obs[2]/data.survdiff$exp[2])

up95 = exp(log(HR) + qnorm(0.975)*sqrt(1/data.survdiff$exp[1]+1/data.survdiff$exp[2]))

low95 = exp(log(HR) - qnorm(0.975)*sqrt(1/data.survdiff$exp[1]+1/data.survdiff$exp[2]))

HRdata=data.frame()

HRdata=cbind(HR,low95,up95)

write.table(HRdata,file=paste0("HRdata.",i ,".txt"),quote=F,sep="\t",row.names = F)}

}

outTab=cbind(outTab, km)

write.table(outTab,file="uniCox.txt",sep="\t",row.names=F,quote=F)

31.

library(igraph)

library(psych)

library(reshape2)

library("RColorBrewer")

GeneExpfile <- ""

Genefile <- "gene.txt"

Coxfile <- "uniCox.txt"

setwd("")

gene.group <- read.table(Genefile,header=T,sep="\t")

gene.exp <- read.table(GeneExpfile,header=T,sep="\t",row.names=1)

gene.cox <- read.table(Coxfile,header=T,sep="\t")

colnames(gene.group) <- c('id','group')

genelist <- intersect(gene.group$id, gene.cox$id)

genelist <- intersect(genelist, rownames(gene.exp))

gene.group <- gene.group[match(genelist,gene.group$id),]

gene.group <- gene.group[order(gene.group$group),]

gene.exp <- gene.exp[match(gene.group$id,rownames(gene.exp)),]

gene.cox <- gene.cox[match(gene.group$id,gene.cox$id),]

gene.cor <- corr.test(t(gene.exp))

gene.cor.cor <- gene.cor$r

gene.cor.pvalue <- gene.cor$p

gene.cor.cor[upper.tri(gene.cor.cor)] = NA

gene.cor.pvalue[upper.tri(gene.cor.pvalue)] = NA

gene.cor.cor.melt <- melt(gene.cor.cor) #gene1 \t gene2 \t cor

gene.cor.pvalue.melt <- melt(gene.cor.pvalue)

gene.melt <- data.frame(from = gene.cor.cor.melt$Var2,to=gene.cor.cor.melt$Var1,cor=gene.cor.cor.melt$value,pvalue=gene.cor.pvalue.melt$value)

gene.melt <- gene.melt[gene.melt$from!=gene.melt$to&!is.na(gene.melt$pvalue),,drop=F]

gene.edge <- gene.melt[gene.melt$pvalue<0.0001,,drop=F]

gene.edge$color <- ifelse(gene.edge$cor>0,'pink','#6495ED')

gene.edge$weight <- abs(gene.edge$cor)*6

gene.node <- gene.group

group.color <- colorRampPalette(brewer.pal(9, "Set1"))(length(unique(gene.node$group)))

gene.node$color <- group.color[as.numeric(as.factor(gene.node$group))]

gene.node$shape <- "circle"

gene.node$frame <- ifelse(gene.cox$HR>1,'purple',"green")

gene.node$pvalue <- gene.cox$pvalue

pvalue.breaks <- c(0,0.0001,0.001,0.01,0.05,1)

pvalue.size <- c(16,14,12,10,8)

cutpvalue <- cut(gene.node$pvalue,breaks=pvalue.breaks)

gene.node$size <- pvalue.size[as.numeric(cutpvalue)]

nodefile <- "network.node.txt"

edgefile <- "network.edge.txt"

write.table(gene.node, nodefile, sep="\t", col.names=T, row.names=F, quote=F)

write.table(gene.edge, edgefile, sep="\t", col.names=T, row.names=F, quote=F)

node = read.table(nodefile, header=T, sep="\t", comment.char="")

edge = read.table(edgefile, header=T, sep="\t", comment.char="")

g = graph.data.frame(edge,directed = FALSE)

node = node[match(names(components(g)$membership),node$id),]

if(!is.na(match('color',colnames(node)))) V(g)$color = node$color

if(!is.na(match('size',colnames(node)))) V(g)$size = node$size

if(!is.na(match('shape',colnames(node)))) V(g)$shape = node$shape

if(!is.na(match('frame',colnames(node)))) V(g)$frame = node$frame

pdf(file="network.pdf", width=10, height=8)

par(mar=c(0,0,0,0))

layout(matrix(c(1,1,4,2,3,4),nc=2),height=c(4,4,2),width=c(8,3))

coord = layout_in_circle(g)

degree.x = acos(coord[,1])

degree.y = asin(coord[,2])

degree.alpha = c()

for(i in 1:length(degree.x)){

if(degree.y[i]<0) degree.alpha=c(degree.alpha,2*pi-degree.x[i]) else degree.alpha=c(degree.alpha,degree.x[i])

}

degree.cut.group = (0:8)/4*pi

degree.cut.group[1] = -0.0001

degree.cut = cut(degree.alpha,degree.cut.group)

degree.degree = c(-pi/4,-pi/4,-pi/2,-pi/2,pi/2,pi/2,pi/2,pi/4)

degree = degree.degree[as.numeric(degree.cut)]

values <- lapply(node$id,function(x)c(1,1))

V(g)$pie.color = lapply(1:nrow(node),function(x)c(node$color[x],node$frame[x]))

V(g)$frame = NA

plot(g,layout=layout_in_circle,vertex.shape="pie",vertex.pie=values,

vertex.label.cex=V(g)$lable.cex,edge.width = E(g)$weight,edge.arrow.size=0,

vertex.label.color=V(g)$color,vertex.frame.color=V(g)$frame,edge.color=E(g)$color,

vertex.label.cex=2,vertex.label.font=2,vertex.size=V(g)$size,edge.curved=0.4,

vertex.color=V(g)$color,vertex.label.dist=1,vertex.label.degree=degree)

par(mar=c(0,0,0,0))

plot(1,type="n",xlab="",ylab="",axes=F)

groupinfo = unique(data.frame(group=node$group,color=node$color))

legend("left",legend=groupinfo$group,col=groupinfo$color,pch=16,bty="n",cex=3)

par(mar=c(0,0,0,0))

plot(1,type="n",xlab="",ylab="",axes=F)

legend("left",legend=c('Risk factors','Favorable factors'),col=c('purple','green'),pch=16,bty="n",cex=2.5)

par(mar=c(0,0,0,0))

plot(1,type="n",xlab="",axes=F,ylab="")

legend("top",legend=c('Postive correlation with P<0.0001','Negative correlation with P<0.0001'),lty=1,lwd=4,col=c('pink','#6495ED'),bty="n",cex=2.2)

legend('bottom',legend=c(0.0001,0.001,0.01,0.05,1),pch=16,pt.cex=c(1.6,1.4,1.2,1,0.8)*6,bty="n",ncol=5,cex=2.2,col="black",title="Cox test, pvalue")

dev.off()

32.

library(ConsensusClusterPlus)

expFile=" "

workDir=" "

setwd(workDir)

data=read.table(expFile, header=T, sep="\t", check.names=F, row.names=1)

data=as.matrix(data)

maxK=6

results=ConsensusClusterPlus(data,

maxK=maxK,

reps=100,

pItem=0.8,

pFeature=1,

title=workDir,

clusterAlg="km",

distance="euclidean",

seed=123456,

plot="png")

clusterNum=2

cluster=results[[clusterNum]][["consensusClass"]]

cluster=as.data.frame(cluster)

colnames(cluster)=c("cluster")

letter=c("A","B","C","D","E","F","G")

uniqClu=levels(factor(cluster$cluster))

cluster$cluster=letter[match(cluster$cluster, uniqClu)]

clusterOut=rbind(ID=colnames(cluster), cluster)

write.table(clusterOut, file=" ", sep="\t", quote=F, col.names=F)

33.

library(survival)

library(survminer)

clusterFile=" "

cliFile="time.txt"

setwd("")

cluster=read.table(clusterFile, header=T, sep="\t", check.names=F, row.names=1)

rownames(cluster)=gsub("(.*?)\\_(.*?)", "\\2", rownames(cluster))

cli=read.table(cliFile, header=T, sep="\t", check.names=F, row.names=1)

colnames(cli)=c("futime", "fustat")

cli$futime=cli$futime/365

sameSample=intersect(row.names(cluster), row.names(cli))

rt=cbind(cli[sameSample,,drop=F], cluster[sameSample,,drop=F])

length=length(levels(factor(rt$cluster)))

diff=survdiff(Surv(futime, fustat) ~ cluster, data = rt)

pValue=1-pchisq(diff$chisq, df=length-1)

if(pValue<0.001){

pValue="p<0.001"

}else{

pValue=paste0("p=",sprintf("%.03f",pValue))

}

fit <- survfit(Surv(futime, fustat) ~ cluster, data = rt)

bioCol=c("#0066FF","#FF0000","#FF9900","#6E568C","#7CC767","#223D6C","#D20A13","#FFD121","#088247","#11AA4D")

bioCol=bioCol[1:length]

surPlot=ggsurvplot(fit,

data=rt,

conf.int=F,

pval=pValue,

pval.size=6,

legend.title="Cluster",

legend.labs=levels(factor(rt[,"cluster"])),

legend = c(0.8, 0.8),

font.legend=10,

xlab="Time(years)",

break.time.by = 1,

palette = bioCol,

risk.table=T,

cumevents=F,

risk.table.height=.25)

pdf(file="survival.pdf",onefile = FALSE,width=7,height=5.5)

print(surPlot)

dev.off()

data.survdiff <- survdiff(Surv(futime, fustat) ~ m6Acluster, data = rt)

p.val = 1 - pchisq(data.survdiff$chisq, length(data.survdiff$n) - 1)

HR = (data.survdiff$obs[2]/data.survdiff$exp[2])/(data.survdiff$obs[1]/data.survdiff$exp[1])

up95 = exp(log(HR) + qnorm(0.975)*sqrt(1/data.survdiff$exp[2]+1/data.survdiff$exp[1]))

low95 = exp(log(HR) - qnorm(0.975)*sqrt(1/data.survdiff$exp[2]+1/data.survdiff$exp[1]))

HRdata=data.frame()

HRdata=cbind(HR,low95,up95)

write.table(HRdata,file="HRdata.txt",quote=F,sep="\t",row.names = F)

34.

library(circlize)

library(ComplexHeatmap)

expFile=" "

clusterFile=" "

cliFile="clinical.txt"

setwd("")

exp=read.table(expFile, header=T, sep="\t", check.names=F, row.names=1)

exp=t(exp)

cluster=read.table(clusterFile, header=T, sep="\t", check.names=F, row.names=1)

sameSample=intersect(row.names(exp), row.names(cluster))

exp=exp[sameSample, , drop=F]

cluster=cluster[sameSample, , drop=F]

expCluster=cbind(exp, cluster)

Project=gsub("(.*?)\\_.*", "\\1", rownames(expCluster))

rownames(expCluster)=gsub("(.*?)\\_(.*?)", "\\2", rownames(expCluster))

expCluster=cbind(expCluster, Project)

cli=read.table(cliFile, header=T, sep="\t", check.names=F, row.names=1)

sameSample=intersect(row.names(expCluster), row.names(cli))

expCluster=expCluster[sameSample,,drop=F]

cli=cli[sameSample,,drop=F]

data=cbind(expCluster, cli)

data=data[order(data$Cluster),]

#Type=data[,((ncol(exp)+1):ncol(data))]

data=t(data[,1:ncol(exp)])

#write.table(Type, file="Type.txt", sep="\t", quote=F, col.names = NA)

Type=read.table("Type.txt", header=T, sep="\t", check.names=F, row.names=1)

anno_df = data.frame(Type)

ha = HeatmapAnnotation(df = anno_df,

col = list(Cluster = c("A" = "blue", "B" = "red"), #, space = "RGB"

Lund.subtype = c("Ba/Sq" = "#DC143C", "Ba/Sq-Inf" = "#FFF0F5", "GU" = "#DB7093", "GU-Inf" = "#FF69B4", "Mes-like" = "#FF1493", "Sc/NE-like" = "#C71585", "Uro-Inf" = "#DA70D6", "UroA-Prog" = "#D8BFD8", "UroB" = "#DDA0DD", "UroC" = "#EE82EE"),#, space = "RGB"

Baylor.subtype = c("Basal" = "#FF00FF", "Differentiated" = "#8B008B"),#, space = "RGB"

UNC.subtype = c("Basal" = "#BA55D3", "Luminal" = "#9400D3"),#, space = "RGB"

CIT.subtype = c("MC1" = "#4B0082", "MC2" = "#8A2BE2", "MC3" = "#9370DB", "MC4" = "#7B68EE", "MC5" = "#6A5ACD", "MC6" = "#483D8B", "MC7" = "#E6E6FA"),#, space = "RGB"

MDA.subtype = c("basal" = "#0000FF", "luminal" = "#00008B", "p53-like" = "#4169E1"),#, space = "RGB"

TCGA.subtype = c("Basal_squamous" = "#4682B4", "Luminal" = "#87CEFA", "Luminal_infiltrated" = "#87CEEB", "Luminal_papillary" = "#00BFFF", "Neuronal" = "#B0E0E6"),#, space = "RGB"

MIBC.subtype = c("Ba/Sq" = "#008080", "LumNS" = "#48D1CC", "LumP" = "#20B2AA", "LumU" = "#7FFFAA", "NE-like" = "#00FA9A", "Stroma-rich" = "#00FF7F"),#, space = "RGB"

N = c("N+" = "#32CD32", "N0" = "#00FF00"),#, space = "RGB"

T = c("Ta-T1" = "#FFFACD", "T2" = "#F0E68C", "T3" = "#FFD700", "T4" = "#DAA520", "T2-4" = "#FFA500"),#, space = "RGB"

Gender = c("Female" = "#F4A460", "Male" = "#D2691E"),

Age = c("<=65" = "#FF7F50", ">65" = "#FF4500"),#, space = "RGB"

NMIBC.subtype = c("Class_1" = "#FFDAB9", "Class_2a" = "#F4A460", "Class_2b" = "#D2691E", "Class_3" = "#8B4513"),#, space = "RGB"

Project = c("E-MTAB-4321" = "#8B008B", "GSE128702" = "#BA55D3", "GSE19423" = "#9400D3", "GSE31684" = "#9932CC", "GSE48075" = "#9370DB", "GSE48276" = "#7B68EE", "GSE87304" = "#6A5ACD", "TCGA" = "#483D8B")),#, space = "RGB"

na_col = "white",

annotation_name_side = "left",simple_anno_size = unit(0.4, "cm"),gap = unit(2, "points"),

annotation_legend_param = list(

T = list(title = "T",

at = c("Ta-T1", "T2","T3","T4","T2-4"),

labels = c("Ta-T1", "T2","T3","T4","T2-4")),

N = list(title = "N",

at = c("N0", "N+"),

labels = c("N0", "N+"))

))

pdf("heatmap.pdf", height=9, width=16)

pheatmap(data,

name = "Expression",

scale="row",

na_col = "white",

color_space = "RGB",

#col = col_fun,

color = colorRampPalette(c(rep("blue",3), "white", rep("red",3)))(50),

column_title = "Bladder Cancer Samples(n=1841)",

cluster_cols =F,

column_split = data.frame(Type$Cluster),

column_gap = unit(0, "mm"), border = TRUE,

show_colnames=F,

show_row_dend = FALSE,

row_names_side = "left",

border_color = NA,

top_annotation = ha

)

dev.off()

35.

library(plyr)

library(ggplot2)

library(ggpubr)

rt="input.txt"

trait="Gender"

setwd("")

rt=read.table(rt, header=T, sep="\t", check.names=F, row.names=1)

bioCol=c("Female" = "#F4A460", "Male" = "#D2691E")

bioCol=bioCol[1:length(unique(rt[,trait]))]

rt1=rt[,c(trait, "Cluster")]

colnames(rt1)=c("trait", "Cluster")

df=as.data.frame(table(rt1))

df=ddply(df, .(Cluster), transform, percent = Freq/sum(Freq) * 100)

df=ddply(df, .(Cluster), transform, pos = (cumsum(Freq) - 0.5 * Freq))

df$label=paste0(sprintf("%.0f", df$percent), "%")

df$Cluster=factor(df$Cluster, levels=c("A", "B"))

p=ggplot(df, aes(x = factor(Cluster), y = percent, fill = trait)) +

geom_bar(position = position_stack(), stat = "identity", width = .7) +

scale_fill_manual(values=bioCol)+

xlab("Cluster")+ ylab(trait)+

guides(fill=guide_legend(title=""))+

geom_text(aes(label = Freq), position = position_stack(vjust = 0.5), size = 3) +

#coord_flip()+

theme_bw()

pdf(file=paste0(trait, "-barplot", ".pdf"), width=5, height=8)

print(p)

dev.off()

36.

library(limma)

library(GSEABase)

library(GSVA)

library(pheatmap)

expFile="merge.txt"

clusterFile=" "

gmtFile="c2.cp.kegg.v7.5.1.symbols.txt"

setwd("")

rt=read.table(expFile, header=T, sep="\t", check.names=F)

rt=as.matrix(rt)

rownames(rt)=rt[,1]

exp=rt[,2:ncol(rt)]

dimnames=list(rownames(exp), colnames(exp))

data=matrix(as.numeric(as.matrix(exp)), nrow=nrow(exp), dimnames=dimnames)

data=avereps(data)

geneSets=getGmt(gmtFile, geneIdType=SymbolIdentifier())

gsvaResult=gsva(data,

geneSets,

min.sz=10,

max.sz=500,

verbose=TRUE,

parallel.sz=1)

gsvaOut=rbind(id=colnames(gsvaResult), gsvaResult)

write.table(gsvaOut, file="gsvaOut.txt", sep="\t", quote=F, col.names=F)

cluster=read.table(clusterFile, header=T, sep="\t", check.names=F, row.names=1)

gsvaResult=t(gsvaResult)

sameSample=intersect(row.names(gsvaResult), row.names(cluster))

gsvaResult=gsvaResult[sameSample,,drop=F]

cluster=cluster[sameSample,,drop=F]

gsvaCluster=cbind(gsvaResult, cluster)

Project=gsub("(.*?)\\_.*", "\\1", rownames(gsvaCluster))

gsvaCluster=cbind(gsvaCluster, Project)

adj.P.Val.Filter=0.05

allType=as.vector(gsvaCluster$Cluster)

comp=combn(levels(factor(allType)), 2)

for(i in 1:ncol(comp)){

treat=gsvaCluster[gsvaCluster$Cluster==comp[2,i],]

con=gsvaCluster[gsvaCluster$Cluster==comp[1,i],]

data=rbind(con, treat)

Type=as.vector(data$Cluster)

ann=data[,c(ncol(data), (ncol(data)-1))]

data=t(data[,-c((ncol(data)-1), ncol(data))])

design=model.matrix(~0+factor(Type))

colnames(design)=levels(factor(Type))

fit=lmFit(data, design)

contrast=paste0(comp[2,i], "-", comp[1,i])

cont.matrix=makeContrasts(contrast, levels=design)

fit2=contrasts.fit(fit, cont.matrix)

fit2=eBayes(fit2)

allDiff=topTable(fit2,adjust='fdr',number=200000)

allDiffOut=rbind(id=colnames(allDiff),allDiff)

write.table(allDiffOut, file=paste0(contrast, ".all.txt"), sep="\t", quote=F, col.names=F)

diffSig=allDiff[with(allDiff, (abs(logFC)>0.1 & adj.P.Val < adj.P.Val.Filter )), ]

diffSigOut=rbind(id=colnames(diffSig),diffSig)

write.table(diffSigOut, file=paste0(contrast, ".diff.txt"), sep="\t", quote=F, col.names=F)

bioCol=c("#0066FF","#FF0000","#FF9900","#6E568C","#7CC767","#223D6C","#D20A13","#FFD121","#088247","#11AA4D")

ann_colors=list()

m6aCluCol=bioCol[1:length(levels(factor(allType)))]

names(m6aCluCol)=levels(factor(allType))

ann_colors[["Cluster"]]=m6aCluCol[c(comp[1,i], comp[2,i])]

ProjectCol=c( "#8B008B","#BA55D3", "#9400D3", "#9932CC", "#9370DB", "#7B68EE", "#6A5ACD", "#483D8B")

names(ProjectCol)=levels(factor(gsvaCluster$Project))

ann_colors[["Project"]]=ProjectCol

termNum=20

diffTermName=as.vector(rownames(diffSig))

diffLength=length(diffTermName)

if(diffLength<termNum){termNum=diffLength}

hmGene=diffTermName[1:termNum]

hmExp=data[hmGene,]

pdf(file=paste0(contrast,".heatmap.pdf"),height=6,width=10)

pheatmap(hmExp,

annotation=ann,

annotation_colors = ann_colors,

border_color = "red",

color = colorRampPalette(c(rep("navy",2), "white", rep("firebrick3",2)))(50),

cutree_rows = 2,

treeheight_row = 0,

cluster_cols =F,

show_colnames = F,

gaps_col=as.vector(cumsum(table(Type))),

scale="row",

fontsize = 10,

fontsize_row=7,

fontsize_col=10)

dev.off()

}

37.

library(reshape2)

library(ggpubr)

library(limma)

library(GSEABase)

library(GSVA)

expFile="merge.txt"

gmtFile="immune.gmt"

clusterFile=" "

setwd("")

rt=read.table(expFile, header=T, sep="\t", check.names=F)

rt=as.matrix(rt)

rownames(rt)=rt[,1]

exp=rt[,2:ncol(rt)]

dimnames=list(rownames(exp),colnames(exp))

data=matrix(as.numeric(as.matrix(exp)),nrow=nrow(exp),dimnames=dimnames)

data=avereps(data)

geneSets=getGmt(gmtFile, geneIdType=SymbolIdentifier())

ssgseaScore=gsva(data, geneSets, method='ssgsea', kcdf='Gaussian', abs.ranking=TRUE)

normalize=function(x){

return((x-min(x))/(max(x)-min(x)))}

ssgseaScore=normalize(ssgseaScore)

ssgseaOut=rbind(id=colnames(ssgseaScore), ssgseaScore)

write.table(ssgseaOut,file="ssGSEA.result.txt",sep="\t",quote=F,col.names=F)

cluster=read.table(clusterFile, header=T, sep="\t", check.names=F, row.names=1)

ssgseaScore=t(ssgseaScore)

sameSample=intersect(row.names(ssgseaScore), row.names(cluster))

ssgseaScore=ssgseaScore[sameSample,,drop=F]

cluster=cluster[sameSample,,drop=F]

scoreCluster=cbind(ssgseaScore, cluster)

data=melt(scoreCluster, id.vars=c("Cluster"))

colnames(data)=c("Cluster", "Immune", "Fraction")

bioCol=c("#0066FF","#FF0000","#FF9900","#6E568C","#7CC767","#223D6C","#D20A13","#FFD121","#088247","#11AA4D")

bioCol=bioCol[1:length(levels(factor(data[,"Cluster"])))]

p=ggboxplot(data, x="Immune", y="Fraction", color="Cluster",

ylab="Immune infiltration",

xlab="",

legend.title="m6Acluster",

palette=bioCol)

p=p+rotate_x_text(50)

pdf(file="boxplot.pdf", width=8, height=6.5)

p+stat_compare_means(aes(group=Cluster),symnum.args=list(cutpoints = c(0,0.00001, 0.0001,0.001, 0.01, 0.05, 1), symbols = c("*****","****","***", "**", "*", "ns")),label = "p.signif")

dev.off()

38.

library(limma)

library(ggplot2)

expFile="GeneExp.txt"

clusterFile="Cluster.txt"

setwd("")

rt=read.table(expFile, header=T, sep="\t", check.names=F)

rt=as.matrix(rt)

rownames(rt)=rt[,1]

exp=rt[,2:ncol(rt)]

dimnames=list(rownames(exp),colnames(exp))

data=matrix(as.numeric(as.matrix(exp)),nrow=nrow(exp),dimnames=dimnames)

data=avereps(data)

data=data[rowMeans(data)>0,]

data=t(data)

data.pca=prcomp(data, scale. = TRUE)

pcaPredict=predict(data.pca)

write.table(pcaPredict, file="newTab.xls", quote=F, sep="\t")

cluster=read.table(clusterFile, header=T, sep="\t", check.names=F, row.names=1)

Ncluster=as.vector(cluster[,1])

bioCol=c("#0066FF","#FF0000","#FF9900","#6E568C","#7CC767","#223D6C","#D20A13","#FFD121","#088247","#11AA4D")

CluCol=bioCol[1:length(levels(factor(Ncluster)))]

PCA=data.frame(PC1=pcaPredict[,1], PC2=pcaPredict[,2], Cluster=Ncluster)

PCA.mean=aggregate(PCA[,1:2], list(Cluster=PCA$Cluster), mean)

pdf(file="PCA.pdf", height=5, width=6.5)

ggplot(data = PCA, aes(PC1, PC2)) + geom_point(aes(color = Cluster)) +

scale_colour_manual(name="Cluster", values =CluCol)+

theme_bw()+

theme(plot.margin=unit(rep(1.5,4),'lines'))+

annotate("text",x=PCA.mean$PC1, y=PCA.mean$PC2, label=PCA.mean$Cluster, cex=7)+

theme(panel.grid.major = element_blank(), panel.grid.minor = element_blank())

dev.off()

39.

library(limma)

library(VennDiagram)

expFile="merge.txt"

cluFile="Cluster.txt"

adj.P.Val.Filter=0.001

setwd("")

rt=read.table(expFile, header=T, sep="\t", check.names=F)

rt=as.matrix(rt)

rownames(rt)=rt[,1]

exp=rt[,2:ncol(rt)]

dimnames=list(rownames(exp),colnames(exp))

data=matrix(as.numeric(as.matrix(exp)),nrow=nrow(exp),dimnames=dimnames)

data=avereps(data)

data=data[rowMeans(data)>0,]

cluster=read.table(cluFile, header=T, sep="\t", check.names=F, row.names=1)

sameSample=intersect(colnames(data), row.names(cluster))

data=data[,sameSample]

cluster=cluster[sameSample,]

logFCfilter=1

geneList=list()

Type=as.vector(cluster)

design=model.matrix(~0+factor(Type))

colnames(design)=levels(factor(Type))

comp=combn(levels(factor(Type)), 2)

allDiffGenes=c()

for(i in 1:ncol(comp)){

fit=lmFit(data, design)

contrast=paste0(comp[2,i], "-", comp[1,i])

#print(contrast)

cont.matrix=makeContrasts(contrast, levels=design)

fit2=contrasts.fit(fit, cont.matrix)

fit2=eBayes(fit2)

allDiff=topTable(fit2,adjust='fdr',number=200000)

allDiffOut=rbind(id=colnames(allDiff),allDiff)

write.table(allDiffOut, file=paste0(contrast, ".all.txt"), sep="\t", quote=F, col.names=F)

diffSig=allDiff[with(allDiff, (abs(logFC)>logFCfilter & adj.P.Val < adj.P.Val.Filter )), ]

diffSigOut=rbind(id=colnames(diffSig),diffSig)

write.table(diffSigOut, file=paste0(contrast, ".diff.txt"), sep="\t", quote=F, col.names=F)

geneList[[contrast]]=row.names(diffSig)

}

interGenes=Reduce(intersect,geneList)

write.table(file="interGene.txt",interGenes,sep="\t",quote=F,col.names=F,row.names=F)

interGeneExp=data[interGenes,]

interGeneExp=rbind(id=colnames(interGeneExp), interGeneExp)

write.table(interGeneExp, file="interGeneExp.txt", sep="\t", quote=F, col.names=F)

40.

library(limma)

library(reshape2)

library(ggpubr)

expFile="GeneExp.txt"

geneCluFile="Cluster.txt"

setwd("")

rt=read.table(expFile, header=T, sep="\t", check.names=F)

rt=as.matrix(rt)

rownames(rt)=rt[,1]

exp=rt[,2:ncol(rt)]

dimnames=list(rownames(exp),colnames(exp))

data=matrix(as.numeric(as.matrix(exp)),nrow=nrow(exp),dimnames=dimnames)

data=avereps(data)

data=t(data)

geneClu=read.table(geneCluFile, header=T, sep="\t", check.names=F, row.names=1)

sameSample=intersect(row.names(data), row.names(geneClu))

expClu=cbind(data[sameSample,,drop=F], geneClu[sameSample,,drop=F])

data=melt(expClu, id.vars=c("Cluster"))

colnames(data)=c("Cluster", "Gene", "Expression")

bioCol=c("#0066FF","#FF0000","#FF9900","#6E568C","#7CC767","#223D6C","#D20A13","#FFD121","#088247","#11AA4D")

bioCol=bioCol[1:length(levels(factor(data[,"Cluster"])))]

p=ggviolin(data, x="Gene", y="Expression", fill = "Cluster",color = "#FF9900",

ylab="Gene expression",

xlab="",

legend.title="Cluster",

add = "boxplot", add.params = list(color="white"),

palette = bioCol,

width=1)

p=p+rotate_x_text(45)

p1=p+stat_compare_means(aes(group=Cluster),

symnum.args=list(cutpoints = c(0,0.00001, 0.0001,0.001, 0.01, 0.05, 1), symbols = c("*****","****","***", "**", "*", " ")),

label = "p.signif")

pdf(file="boxplot.pdf", width=9, height=5)

print(p1)

dev.off()

41.

expFile="interGeneExp.txt"

setwd("")

data=read.table(expFile, header=T, sep="\t", check.names=F, row.names=1)

data=t(data)

pca=prcomp(data, scale=TRUE)

value=predict(pca)

Necroscore=value[,1]

Necroscore=as.data.frame(Necroscore)

scoreOut=rbind(id=colnames(Necroscore), Necroscore)

write.table(scoreOut, file="Necroscore.txt", sep="\t", quote=F, col.names=F)

42.

library(survival)

library(survminer)

scoreFile="Necroscore.txt"

cliFile="time.txt"

setwd("")

Necroscore=read.table(scoreFile, header=T, sep="\t", check.names=F, row.names=1)

sampleType=gsub("(.*?)\\_.*", "\\1", row.names(Necroscore))

Necroscore=cbind(Necroscore, sampleType)

rownames(Necroscore)=gsub("(.*?)\\_(.*?)", "\\2", rownames(Necroscore))

cli=read.table(cliFile, header=T, sep="\t", check.names=F, row.names=1)

colnames(cli)=c("futime", "fustat")

cli$futime=cli$futime/365

sameSample=intersect(row.names(Necroscore), row.names(cli))

data=cbind(cli[sameSample,], Necroscore[sameSample,])

res.cut=surv_cutpoint(data, time="futime", event="fustat", variables=c("Necroscore"))

cutoff=as.numeric(res.cut$cutpoint[1])

#cutoff=8.5

print(cutoff)

Type=ifelse(data[,"Necroscore"]<=cutoff, "Low", "High")

data$group=Type

outTab=rbind(id=colnames(data), data)

write.table(outTab, file="Necroscore.group.txt", sep="\t", quote=F, col.names=F)

data$group=factor(data$group, levels=c("Low", "High"))

diff=survdiff(Surv(futime, fustat) ~ group, data = data)

length=length(levels(factor(data[,"group"])))

pValue=1-pchisq(diff$chisq, df=length-1)

if(pValue<0.001){

pValue="p<0.001"

}else{

pValue=paste0("p=",sprintf("%.03f",pValue))

}

fit <- survfit(Surv(futime, fustat) ~ group, data = data)

bioCol=c("#0066FF","#FF0000","#6E568C","#7CC767","#223D6C","#D20A13","#FFD121","#088247","#11AA4D")

bioCol=bioCol[1:length]

surPlot=ggsurvplot(fit,

data=data,

conf.int=F,

pval=pValue,

pval.size=6,

legend.title="Necroscore",

legend.labs=levels(factor(data[,"group"])),

legend = c(0.8, 0.8),

font.legend=12,

xlab="Time(years)",

break.time.by = 1,

palette = bioCol,

#surv.median.line = "hv",

risk.table=T,

cumevents=F,

risk.table.height=.25)

pdf(file="survival.pdf", onefile = FALSE, width=7, height=5.5)

print(surPlot)

dev.off()

data.survdiff <- survdiff(Surv(futime, fustat) ~ group, data = data)

p.val= 1 - pchisq(data.survdiff$chisq, length(data.survdiff$n) - 1)

HR.1= (data.survdiff$obs[2]/data.survdiff$exp[2])/(data.survdiff$obs[1]/data.survdiff$exp[1])

up95.1 = exp(log(HR.1) + qnorm(0.975)*sqrt(1/data.survdiff$exp[2]+1/data.survdiff$exp[1]))

low95.1 = exp(log(HR.1) - qnorm(0.975)*sqrt(1/data.survdiff$exp[2]+1/data.survdiff$exp[1]))

HRdata.1=data.frame()

HRdata.1=cbind(HR.1,low95.1,up95.1,p.val)

write.table(HRdata.1,file="HRdata.txt",quote=F,sep="\t",row.names = F)

43.

library(ggalluvial)

library(ggplot2)

library(dplyr)

setwd("")

m6aClu=read.table("m6aCluster.txt", header=T, sep="\t", check.names=F, row.names=1)

Tide=read.table("tide.txt", header=T, sep="\t", check.names=F, row.names=1)

score=read.table("Necroscore.txt", header=T, sep="\t", check.names=F, row.names=1)

cli=read.table("T.txt", header=T, sep="\t", check.names=F, row.names=1)

subtype=read.table("subtype.txt", header=T, sep="\t", check.names=F, row.names=1)

rownames(Tide)=gsub("(.*?)\\_(.*?)", "\\2", rownames(Tide))

sameSample3=intersect(row.names(m6aClu), row.names(score))

twoCluster=cbind(m6aClu[sameSample3,,drop=F], score[sameSample3,,drop=F])

rownames(twoCluster)=gsub("(.*?)\\_(.*?)", "\\2", rownames(twoCluster))

sameSample1=intersect(row.names(twoCluster), row.names(subtype))

sameSample2=intersect(row.names(Tide),row.names(cli))

sameSample=intersect(sameSample1,sameSample2)

rt=cbind(twoCluster[sameSample,,drop=F],subtype[sameSample,,drop=F],Tide[sameSample,,drop=F],cli[sameSample,,drop=F])

write.table(rt,file="all-input.txt",quote = F,sep="\t",col.names=NA)

colnames(rt)=c("Cluster", "Necroscore","NMIBC-TCGA.subtype", "CTL.flag","T")

#colnames(rt)=c("Cluster", "Necroscore", "CTL.flag")

corLodes=to_lodes_form(rt, axes = 1:ncol(rt), id = "Cohort")

pdf(file="ggalluvial.pdf", width=8, height=7)

mycol=rep(c("#0066FF","#FF9900","#FF0000","#029149","#6E568C","#E0367A","#D8D155","#223D6C","#D20A13","#431A3D","#91612D","#FFD121","#088247","#11AA4D","#58CDD9","#7A142C","#5D90BA","#64495D","#7CC767"),15)

ggplot(corLodes, aes(x = x, stratum = stratum, alluvium = Cohort,fill = stratum, label = stratum)) +

scale_x_discrete(expand = c(0, 0)) +

geom_flow(width = 2/10,aes.flow = "forward") +

geom_stratum(alpha = .9,width = 2/10) +

scale_fill_manual(values = mycol) +

geom_text(stat = "stratum", size = 3,color="black") +

xlab("") + ylab("") + theme_bw() +

theme(axis.line = element_blank(),axis.ticks = element_blank(),axis.text.y = element_blank()) +

theme(panel.grid =element_blank()) +

theme(panel.border = element_blank()) +

ggtitle("") + guides(fill = "none")

dev.off()

44.

library(corrplot)

scoreFile="Necroscore.txt"

immFile="ssGSEA.result.txt"

setwd("")

score=read.table(scoreFile, header=T, sep="\t", check.names=F, row.names=1)

immune=read.table(immFile, header=T, sep="\t", check.names=F, row.names=1)

immune=t(immune)

sameSample=intersect(row.names(score), row.names(immune))

data=cbind(score[sameSample,,drop=F], immune[sameSample,,drop=F])

M=cor(data)

res1=cor.mtest(data, conf.level = 0.95)

write.table(M, file="cor.txt", sep="\t", row.names=T, quote=F)

#res1$p[lower.tri(res1$p, diag = TRUE)] <- ""

write.table(res1$p, "cor-p.txt", quote = F, sep = "\t", row.names = F)

pdf(file="cor.pdf", width=8, height=8)

corrplot(M,

order="original",

method = "circle",

type = "upper",

tl.cex=0.8, pch=T,

p.mat = res1$p,

insig = "label_sig",

pch.cex = 1.6,

sig.level=0.05,

number.cex = 1,

col=colorRampPalette(c("blue", "white", "red"))(50),

tl.col="black")

dev.off()

45.

library(beeswarm)

library(limma)

library(ggpubr)

yMin=-20

yMax=30

CluFile="Cluster.txt"

scoreFile="Necroscore.txt"

setwd("")

Clu=read.table(m6aCluFile, header=T, sep="\t", check.names=F, row.names=1)

score=read.table(scoreFile, header=T, sep="\t", check.names=F, row.names=1)

twoCluster=cbind(Clu, geneClu)

sameSample=intersect(row.names(Clu), row.names(score))

data=cbind(score[sameSample,,drop=F], Clu[sameSample,,drop=F])

data$Cluster=factor(data$Cluster, levels=levels(factor(data$Cluster)))

group=levels(factor(data$Cluster))

comp=combn(group, 2)

my_comparisons=list()

for(i in 1:ncol(comp)){my_comparisons[[i]]<-comp[,i]}

wilcoxTest=wilcox.test(Necroscore ~ Cluster, data = data)

pvalue=wilcoxTest$p.value

if(pvalue<0.00001){

pvalue="<0.00001"

}else{

pvalue=paste0("=",sprintf("%.05f",pvalue))

}

bioCol=c("#0066FF","#FF0000","#FF9900","#6E568C","#7CC767","#223D6C","#D20A13","#FFD121","#088247","#11AA4D")

bioCol=bioCol[1:length(levels(factor(data$Cluster)))]

pdf(file="Cluster.pdf", width=5, height=5)

boxplot(Necroscore ~ Cluster, data = data,names=labels,xlab="Cluster",

ylab = paste("Necroscore",sep=""),

cex.main=1.5, cex.lab=1.3, cex.axis=1.2,ylim=c(yMin,yMax),outline = FALSE)

+beeswarm(Necroscore ~ Cluster, data = data, col = c("blue","red"),lwd=0.1,cex=0.5,

pch = 16, add = TRUE, corral="wrap")

ySeg=yMax*0.94

segments(1,ySeg, 2,ySeg);segments(1,ySeg, 1,ySeg*0.96);segments(2,ySeg, 2,ySeg*0.96)

text(1.5,ySeg*1.05,labels=paste("p",pvalue,sep=""),cex=1.2)

dev.off()

46.

library(survival)

library(survminer)

tmbFile="TMB.txt"

scoreFile="Necroscore.group.txt"

setwd("")

score=read.table(scoreFile, header=T, sep="\t", check.names=F, row.names=1)

tmb=read.table(tmbFile, header=T, sep="\t", check.names=F, row.names=1)

sameSample=intersect(row.names(tmb), row.names(score))

tmb=tmb[sameSample,,drop=F]

score=score[sameSample,,drop=F]

data=cbind(score, tmb)

res.cut=surv_cutpoint(data, time = "futime", event = "fustat", variables =c("TMB"))

cutoff=as.numeric(res.cut$cutpoint[1])

tmbType=ifelse(data[,"TMB"]<=cutoff, "L-TMB", "H-TMB")

scoreType=ifelse(data$group=="Low", "L-Necroscore", "H-Necroscore")

mergeType=paste0(scoreType, "+",tmbType )

bioSurvival=function(surData=null, outFile=null){

diff=survdiff(Surv(futime, fustat) ~ group, data=surData)

length=length(levels(factor(surData[,"group"])))

pValue=1-pchisq(diff$chisq, df=length-1)

if(pValue<0.001){

pValue="p<0.001"

}else{

pValue=paste0("p=",sprintf("%.03f",pValue))

}

fit <- survfit(Surv(futime, fustat) ~ group, data = surData)

width=6.5

height=5.5

if(length(levels(factor(surData[,"group"])))>2){

width=8

height=6.5

}

bioCol=c("#FF0000","#6E568C","#0066FF","#7CC767","#223D6C","#D20A13","#FFD121","#088247","#11AA4D")

bioCol=bioCol[1:length]

surPlot=ggsurvplot(fit,

data=surData,

conf.int=F,

pval=pValue,

pval.size=6,

legend.title="",

legend.labs=levels(factor(surData[,"group"])),

font.legend=10,

legend = c(0.8, 0.8),

xlab="Time(years)",

break.time.by = 1,

palette = bioCol,

#surv.median.line = "hv",

risk.table=T,

cumevents=F,

risk.table.height=.25)

pdf(file=outFile, onefile = FALSE, width=width, height=height)

print(surPlot)

dev.off()

}

data$group=tmbType

bioSurvival(surData=data, outFile="TMB.survival.pdf")

data.survdiff <- survdiff(Surv(futime, fustat) ~ tmbType, data = data)

p.val = 1 - pchisq(data.survdiff$chisq, length(data.survdiff$n) - 1)

HR = (data.survdiff$obs[2]/data.survdiff$exp[2])/(data.survdiff$obs[1]/data.survdiff$exp[1])

up95 = exp(log(HR) + qnorm(0.975)*sqrt(1/data.survdiff$exp[2]+1/data.survdiff$exp[1]))

low95 = exp(log(HR) - qnorm(0.975)*sqrt(1/data.survdiff$exp[2]+1/data.survdiff$exp[1]))

HRdata=data.frame()

HRdata=cbind(HR,low95,up95,p.val)

row.names(HRdata)="L-H"

write.table(HRdata,file="tmb-HRdata.txt",quote=F,sep="\t",row.names = T,col.names=NA)

data$group=mergeType

bioSurvival(surData=data, outFile="TMB-Necroscore.survival.pdf")

data.survdiff <- survdiff(Surv(futime, fustat) ~ mergeType, data = data)

p.val= 1 - pchisq(data.survdiff$chisq, length(data.survdiff$n) - 1)

HR.1= (data.survdiff$obs[1]/data.survdiff$exp[1])/(data.survdiff$obs[3]/data.survdiff$exp[3])

up95.1 = exp(log(HR.1) + qnorm(0.975)*sqrt(1/data.survdiff$exp[1]+1/data.survdiff$exp[3]))

low95.1 = exp(log(HR.1) - qnorm(0.975)*sqrt(1/data.survdiff$exp[1]+1/data.survdiff$exp[3]))

HRdata.1=data.frame()

HRdata.1=cbind(HR.1,low95.1,up95.1,p.val)

HR.2 = (data.survdiff$obs[4]/data.survdiff$exp[4])/(data.survdiff$obs[3]/data.survdiff$exp[3])

up95.2 = exp(log(HR.2) + qnorm(0.975)*sqrt(1/data.survdiff$exp[4]+1/data.survdiff$exp[3]))

low95.2 = exp(log(HR.2) - qnorm(0.975)*sqrt(1/data.survdiff$exp[4]+1/data.survdiff$exp[3]))

HRdata.2=data.frame()

HRdata.2=cbind(HR.2,low95.2,up95.2,p.val)

HR.3 = (data.survdiff$obs[2]/data.survdiff$exp[2])/(data.survdiff$obs[3]/data.survdiff$exp[3])

up95.3 = exp(log(HR.3) + qnorm(0.975)*sqrt(1/data.survdiff$exp[2]+1/data.survdiff$exp[3]))

low95.3 = exp(log(HR.3) - qnorm(0.975)*sqrt(1/data.survdiff$exp[2]+1/data.survdiff$exp[3]))

HRdata.3=data.frame()

HRdata.3=cbind(HR.3,low95.3,up95.3,p.val)

HR.4 = (data.survdiff$obs[2]/data.survdiff$exp[2])/(data.survdiff$obs[1]/data.survdiff$exp[1])

up95.4 = exp(log(HR.4) + qnorm(0.975)*sqrt(1/data.survdiff$exp[2]+1/data.survdiff$exp[1]))

low95.4 = exp(log(HR.4) - qnorm(0.975)*sqrt(1/data.survdiff$exp[2]+1/data.survdiff$exp[1]))

HRdata.4=data.frame()

HRdata.4=cbind(HR.4,low95.4,up95.4,p.val)

HR.5 = (data.survdiff$obs[2]/data.survdiff$exp[2])/(data.survdiff$obs[4]/data.survdiff$exp[4])

up95.5 = exp(log(HR.5) + qnorm(0.975)*sqrt(1/data.survdiff$exp[2]+1/data.survdiff$exp[4]))

low95.5 = exp(log(HR.5) - qnorm(0.975)*sqrt(1/data.survdiff$exp[2]+1/data.survdiff$exp[4]))

HRdata.5=data.frame()

HRdata.5=cbind(HR.5,low95.5,up95.5,p.val)

HR.6 = (data.survdiff$obs[4]/data.survdiff$exp[4])/(data.survdiff$obs[1]/data.survdiff$exp[1])

up95.6 = exp(log(HR.6) + qnorm(0.975)*sqrt(1/data.survdiff$exp[4]+1/data.survdiff$exp[1]))

low95.6 = exp(log(HR.6) - qnorm(0.975)*sqrt(1/data.survdiff$exp[4]+1/data.survdiff$exp[1]))

HRdata.6=data.frame()

HRdata.6=cbind(HR.6,low95.6,up95.6,p.val)

HRdata=rbind(HRdata.1,HRdata.2,HRdata.3,HRdata.4,HRdata.5,HRdata.6)

row.names(HRdata)=c("HH-LH","LL-LH","HL-LH","HL-HH","HL-LL","LL-HH")

write.table(HRdata,file="TMB-Necroscore-HRdata.txt",quote=F,sep="\t",row.names = T,col.names=NA)

47.

library(GOplot)

setwd("")

ego=read.table("Hall-AB.txt", header = T,sep="\t",check.names=F)

go=data.frame(Category = "All",ID = ego$ID,Term = ego$Description, Genes = gsub("/", ", ", ego$core_enrichment), adj_pval = ego$p.adjust)

id.fc <- read.table("tcga.all.xls", header = T,sep="\t",check.names=F)

genelist <- data.frame(ID = id.fc$gene, logFC = id.fc$logFC)

row.names(genelist)=genelist[,1]

circ <- circle_dat(go, genelist)

write.table(circ,file=paste0("circ.txt"),sep="\t",quote=F,col.names = NA)

termNum = 12

geneNum = nrow(genelist)

chord <- chord_dat(circ, genelist[1:geneNum,], go[c(1,2,5,6,7,8,9,11,12,13),3])

pdf(file="circ.pdf",width = 11,height = 11)

GOChord(chord,

space = 0.001,

gene.order = 'logFC',

gene.space = 0.25,

gene.size = 3,

border.size = 0.05,

process.label = 12)

dev.off()

termCol <- c("#223D6C","#D20A13","#FFD121","#088247","#58CDD9","#7A142C","#5D90BA","#431A3D","#91612D","#6E568C","#E0367A","#D8D155","#64495D","#7CC767")

pdf(file="cluster.pdf",width = 10,height = 10)

GOCluster(circ.gsym,

go[c(1,4,5,6,7,8,9,10,11,13,21,24),3],

lfc.space = 0.3,

lfc.width = 1,

term.col = termCol[1:termNum],

term.space = 0.3,

term.width = 1

)

dev.off()

48.

inputFile="merge.txt"

setwd("")

source("ICI12.CIBERSORT.R")

outTab=CIBERSORT("ref.txt", inputFile, perm=100, QN=TRUE)

outTab=outTab[outTab[,"P-value"]<0.05,]

outTab=as.matrix(outTab[,1:(ncol(outTab)-3)])

outTab=rbind(id=colnames(outTab), outTab)

write.table(outTab, file="CIBERSORT-Results.txt", sep="\t", quote=F, col.names=F)

49.

library(limma)

library(estimate)

setwd("")

uniqFile="merge.txt"

inputDs="commonGenes.gct"

outputDs="estimateScore.gct"

filterCommonGenes(input.f=uniqFile, output.f=inputDs, id="GeneSymbol")

estimateScore(input.ds =inputDs ,output.ds=outputDs)

scores=read.table("estimateScore.gct",skip = 2,header = T)

rownames(scores)=scores[,1]

scores=t(scores[,3:ncol(scores)])

rownames(scores)=gsub("\\.","\\-",rownames(scores))

out=rbind(ID=colnames(scores), scores)

write.table(out,file="estimateScores.txt",sep="\t",quote=F,row.names=T)

50.

library(corrplot)

cellFile="epic.txt"

scoreFile=" "

setwd("")

cell=read.table(cellFile, header=T, sep="\t", check.names=F, row.names=1)

cell=t(cell)

score=read.table(scoreFile, header=T, sep="\t", check.names=F, row.names=1)

row.names(score)=gsub("(.*?)\\_(.*?)","\\2",row.names(score))

sameSample=intersect(row.names(cell), row.names(score))

cell=cell[sameSample,]

score=score[sameSample,]

data=t(cbind(cell, score[,1:4]))

data=t(data)

M=cor(data)

write.table(M, "cor.txt", quote = F, sep = "\t", row.names = T)

res1=cor.mtest(data, conf.level = 0.95)

write.table(res1$p, "cor-p.txt", quote = F, sep = "\t", row.names = T)

pdf(file="epic-immCor.pdf", width=8, height=8)

corrplot(M,

order="original",

method = 'ellipse',

type = "upper",

tl.cex=0.8, pch=T,

p.mat = res1$p,

insig = "label_sig",

pch.cex = 1.6,

number.cex = 1,

sig.level=0.05,

col=colorRampPalette(c("blue", "white", "red"))(50),

tl.col="black")

dev.off()

51.

library(ggpubr)

library(limma)

library(ggplot2)

rt="input.txt"

setwd("")

rt=read.table(rt, header=T, sep="\t", check.names=F)

rt$group <- factor(ifelse(rt$Correlation < 0, "low", "high"),

levels = c("low", "high"))

boxplot=ggbarplot(rt, x = "Immune Type", y = "Correlation",

fill = "group", #change fill color by mpg_level

color = "white", # Set bar border colors to white

palette = "jco",# jco journal color palett. see ?ggpar

sort.val = "asc", # Sort the value in ascending order

sort.by.groups = FALSE,# Don't sort inside each group

x.text.angle = 90,# Rotate vertically x axis texts

ylab = "Correlation between Necroscore and Immune Infiltrate",

xlab = FALSE,

legend.title = "Correlation group",

#rotate = TRUE

)

pdf(file="boxplot-de25.pdf",width=16,height=6)

print(boxplot)

dev.off()

52.

library(plyr)

library(ggplot2)

library(ggpubr)

scoreFile="Necroscore.txt"

cliFile="tcga-immune.txt"

trait=" "

setwd("")

score=read.table(scoreFile, header=T, sep="\t", check.names=F, row.names=1)

row.names(score)=gsub("(.*?)\\_(.*?)","\\2",row.names(score))

cli=read.table(cliFile, header=F, sep="\t", check.names=F, row.names=1)

row.names(cli)=gsub("(.*?)\\-(.*?)\\-(.*?)\\-.*", "\\1\\-\\2\\-\\3", row.names(cli))

sameSample=intersect(row.names(score), row.names(cli))

rt=cbind(score[sameSample,,drop=F], cli[sameSample,,drop=F])

rt=as.data.frame(rt)

colnames(rt)=c("Necroscore","InflammatoryInfiltrateResponseTSPresent")

bioCol=c("#0066FF","#FF0000","#FF9900","#6E568C","#7CC767","#223D6C","#D20A13","#FFD121","#088247","#11AA4D")

bioCol=bioCol[1:length(unique(rt[,trait]))]

rt2=rt[,c(trait, "Necroscore")]

colnames(rt2)=c("trait", "Necroscore")

type=levels(factor(rt2[,"trait"]))

comp=combn(type, 2)

my_comparisons=list()

for(i in 1:ncol(comp)){my_comparisons[[i]]<-comp[,i]}

boxplot=ggviolin(rt2, x="trait", y="Necroscore", fill="trait",

xlab="Inflammatory Infiltrate",

ylab="Necroscore",

legend.title="Inflammatory Infiltrate",

palette=bioCol,

notch = TRUE,

#order=c("CR","PR","PD","SD"),

#add = c("jitter","boxplot"),

add = c("jitter","boxplot"),

add.params = list(color ="#FF9900",fill = "white",size=1.2)

)+

stat_compare_means(comparisons=my_comparisons)

pdf(file="2-boxplot.pdf",width=5,height=4.5)

print(boxplot)

dev.off()

53.

library(survival)

library(survminer)

library(timeROC)

library(rms)

library(regplot)

#riskFile="trainRisk.txt"

cliFile="input-nomo.txt"

setwd("")

cli=read.table(cliFile, header=T, sep="\t", check.names=F, row.names=1)

cli=cli[apply(cli,1,function(x)any(is.na(match('unknow',x)))),,drop=F]

rt=cli

str(rt)

rt$futime <- as.numeric(rt$futime)

rt$futime <-rt$futime/365

rt$fustat <- as.numeric(rt$fustat)

res.cox=coxph(Surv(futime, fustat) ~ . , data = rt)

nom1=regplot(res.cox,

plots = c("density", "boxes"),

clickable=F,

title="Survival nomogram",

points=TRUE,

droplines=TRUE,

observation=rt[1,],

rank=NULL,

failtime = c(1,3,5),

prfail = T,

interval = NULL)

nomoRisk=predict(res.cox, data=rt, type="risk")

nomorisk

rt$nomoRisk=nomoRisk

ROC_rt=timeROC(T=rt$futime, delta=rt$fustat,

marker=rt$nomoRisk, cause=1,

weighting='aalen',

times=c(1,2,3,4,5), ROC=TRUE)

pdf(file="ROC-necroscore.pdf", width=5, height=5)

plot(ROC_rt,time=1,col='green',title=FALSE,lwd=2)

plot(ROC_rt,time=2,col='yellow',add=TRUE,title=FALSE,lwd=2)

plot(ROC_rt,time=3,col='blue',add=TRUE,title=FALSE,lwd=2)

plot(ROC_rt,time=4,col='gray',add=TRUE,title=FALSE,lwd=2)

plot(ROC_rt,time=5,col='red',add=TRUE,title=FALSE,lwd=2)

legend('bottomright',

c(paste0('AUC at 1 years: ',sprintf("%.03f",ROC_rt$AUC[1])),

paste0('AUC at 2 years: ',sprintf("%.03f",ROC_rt$AUC[2])),

paste0('AUC at 3 years: ',sprintf("%.03f",ROC_rt$AUC[3])),

paste0('AUC at 4 years: ',sprintf("%.03f",ROC_rt$AUC[4])),

paste0('AUC at 5 years: ',sprintf("%.03f",ROC_rt$AUC[5]))),

col=c("green","yellow","blue","gray","red"),lwd=2,bty = 'n')

dev.off()

pdf(file="calibration.pdf", width=5, height=5)

f <- cph(Surv(futime, fustat) ~ nomoRisk, x=T, y=T, surv=T, data=rt, time.inc=1)

cal <- calibrate(f, cmethod="KM", method="boot", u=1, m=(nrow(rt)/3), B=1000)

plot(cal, xlim=c(0,1), ylim=c(0,1),

xlab="Nomogram-predicted OS (%)", ylab="Observed OS (%)", lwd=1.5, col="green", sub=F)

f <- cph(Surv(futime, fustat) ~ nomoRisk, x=T, y=T, surv=T, data=rt, time.inc=3)

cal <- calibrate(f, cmethod="KM", method="boot", u=3, m=(nrow(rt)/3), B=1000)

plot(cal, xlim=c(0,1), ylim=c(0,1), xlab="", ylab="", lwd=1.5, col="blue", sub=F, add=T)

f <- cph(Surv(futime, fustat) ~ nomoRisk, x=T, y=T, surv=T, data=rt, time.inc=5)

cal <- calibrate(f, cmethod="KM", method="boot", u=5, m=(nrow(rt)/3), B=1000)

plot(cal, xlim=c(0,1), ylim=c(0,1), xlab="", ylab="", lwd=1.5, col="red", sub=F, add=T)

legend('bottomright', c('1-year', '3-year', '5-year'),

col=c("green","blue","red"), lwd=1.5, bty = 'n')

dev.off()

54.

library(survival)

library(survcomp)

library(ggplot2)

library(ggpubr)

inputFile="input.txt"

setwd("")

rt=read.table(inputFile, header=T, sep="\t", check.names=F, row.names=1)

df=data.frame()

for(i in colnames(rt)[3:ncol(rt)]){

cindex=concordance.index(x=rt[,i], surv.time=rt$futime, surv.event=rt$fustat,method="noether")

df=rbind(df, cbind(i,sprintf("%.03f",cindex$c.index)))

}

colnames(df)=c("signature", "cindex")

df[,"cindex"]=as.numeric(df[,"cindex"])

color=rainbow(nrow(df),alpha=0.75)

p=ggbarplot(df, x="signature", y="cindex", fill="signature",

xlab="", ylab="C-index", add = "none",

palette=color,

label=T, legend="")

p=p+rotate_x_text(50)

p=p+ylim(0,round(max(df[,"cindex"])+0.15,1))

pdf(file="C-index.pdf", width=6, height=5)

print(p)

dev.off()

outdata=list()

legendsname=c()

rt$futime=rt$futime/365

for(i in 3:ncol(rt)){

OS=Surv(rt$futime, rt$fustat)

marker=rt[,i]

marker.pp<-seq(from=0, to=1, length=100)

marker.qq<-quantile(marker,marker.pp)

fitdat.df<-data.frame(marker=marker)

newdat.df<-data.frame(marker=marker.qq)

cox.model<-coxph(OS~marker, data=fitdat.df)

rms.calc <-summary(survfit(cox.model, newdata=newdat.df))

rms.mean <-rms.calc$table[,"rmean"]

name=colnames(rt)[i]

HR=sprintf("%.03f", summary(cox.model)$conf.int[,"exp(coef)"])

HR.95L=sprintf("%.03f", summary(cox.model)$conf.int[,"lower .95"])

HR.95H=sprintf("%.03f", summary(cox.model)$conf.int[,"upper .95"])

pvalue=summary(cox.model)$coefficients[,"Pr(>|z|)"]

p=ifelse(pvalue<0.001,"p<0.001",paste0("p=",sprintf("%.03f",pvalue)))

legendsname=c(legendsname,paste0(name,", HR:",HR,"(",HR.95L,"-",HR.95H,"), ",p))

outdata[[name]]= data.frame(marker.pp,rms.mean)

}

alldata=do.call("rbind",outdata)

xlim2=max(alldata$rms.mean)

pdf(file="RMS.pdf", width=6, height=6)

par(las=1)

plot(1,xlim=c(0,1),ylim=c(0,xlim2),type="n",xlab="Percentile of scores",ylab="RMS (years)")

names=names(outdata)

for(i in 1:length(outdata)){

namei=names[i]

outdatai=outdata[[namei]]

points(outdatai$marker.pp,outdatai$rms.mean,col=color[i],pch=20,cex=0.8)

}

legend("bottomleft",legend=legendsname,col=color,pch=20,bty="n",cex=1)

dev.off()
